# Changes in transcriptional orientation are associated with increases in evolutionary rates of enterobacterial genes

**DOI:** 10.1186/1471-2105-12-S9-S19

**Published:** 2011-10-05

**Authors:** Chieh-Hua Lin, Chun-Yi Lian, Chao Agnes Hsiung, Feng-Chi Chen

**Affiliations:** 1 Division of Biostatistics and Bioinformatics, Institute of Population Health Sciences, National Health Research Institutes, 35 Keyen Road, Zhunan Town, Miaoli County, Taiwan, Republic of China; 2 Institute of Bioinformatics and Structural Biology, National Tsing Hua University, No. 101, Section 2, Kuang-Fu Road, Hsinchu, Taiwan, Republic of China

## Abstract

**Background:**

Changes in transcriptional orientation (“CTOs”) occur frequently in prokaryotic genomes. Such changes usually result from genomic inversions, which may cause a conflict between the directions of replication and transcription and an increase in mutation rate. However, CTOs do not always lead to the replication-transcription confrontation. Furthermore, CTOs may cause deleterious disruptions of operon structure and/or gene regulations. The currently existing CTOs may indicate relaxation of selection pressure. Therefore, it is of interest to investigate whether CTOs have an independent effect on the evolutionary rates of the affected genes, and whether these genes are subject to any type of selection pressure in prokaryotes.

**Methods:**

Three closely related enterbacteria, *Escherichia coli*, *Klebsiella pneumoniae* and *Salmonella enterica serovar Typhimurium*, were selected for comparisons of synonymous (*dS*) and nonsynonymous (*dN*) substitution rate between the genes that have experienced changes in transcriptional orientation (changed-orientation genes, “COGs”) and those that do not (same-orientation genes, “SOGs”). The *dN*/*dS* ratio was also derived to evaluate the selection pressure on the analyzed genes. Confounding factors in the estimation of evolutionary rates, such as gene essentiality, gene expression level, replication-transcription confrontation, and decreased *dS* at gene terminals were controlled in the COG-SOG comparisons.

**Results:**

We demonstrate that COGs have significantly higher *dN* and *dS* than SOGs when a series of confounding factors are controlled. However, the *dN*/*dS* ratios are similar between the two gene groups, suggesting that the increase in *dS* can sufficiently explain the increase in *dN* in COGs. Therefore, the increases in evolutionary rates in COGs may be mainly mutation-driven.

**Conclusions:**

Here we show that CTOs can increase the evolutionary rates of the affected genes. This effect is independent of the replication-transcription confrontation, which is suggested to be the major cause of inversion-associated evolutionary rate increases. The real cause of such evolutionary rate increases remains unclear but is worth further explorations.

## Introduction

Genome rearrangements occur frequently in the evolution of prokaryotes. Among the rearrangement events, inversions usually occur symmetrically around the origin (designated as “*Ori*”) or terminus (“*Ter*”) of replication between closely related bacterial genomes [1]. These rearrangement events often lead to changes in transcriptional orientation (designated as “CTOs”) [2] and increases in mutation pressure in the affected genes because of a conflict between the directions of transcription and replication [1,3,4]. However, when the inversion events result from the flipping across *Ori* or *Ter*, replication-transcription conflict may not occur [1]. Furthermore, CTOs may cause changes in homology-based recombination or impediments in DNA replication (by altering, for example, DNA protein binding sites or secondary structure), which may increase mutation rate. Meanwhile, CTOs may lead to disruptions of operon structure and transcriptional regulations, which are potentially deleterious. Therefore, currently existing CTOs may signify relaxation of selection pressure on the affected genes. Nevertheless, it remains unknown whether selection actually plays an important role in maintaining CTOs. In view of the potential influences of CTOs on gene evolution, we are interested in investigating whether CTO *per se* has any effect on the evolutionary rates of prokaryotic genes.

Note that the evolution of prokaryotic genes is driven by two major forces: mutation and natural selection. An increased mutation rate can accelerate short-term nucleotide substitutions, which are then retained or eliminated by natural selection according to their fitness effects. The evolutionary rates of prokaryotic genes mainly reflect the combinatorial effects of these two forces. In general, substitutions that cause protein sequence changes (nonsynonymous substitutions) are subject to stronger selection pressure than those that do not (synonymous substitutions). By comparing nonsynonymous (*dN*) and synonymous substitution rate (*dS*), we may evaluate whether mutation or selection plays a more important role in the evolution of prokaryotic genes.

Here we attempt to examine (1) the effects of CTOs on prokaryotic gene evolution; and (2) the relative contributions of the two abovementioned driving forces to *dS* and *dN* in the genes that have experienced CTOs. To this end, we compared the *dS* and *dN* between the genes that have experienced CTOs (changed-orientation genes, “COGs”) and those that do not (same-orientation genes, “SOGs”) in closed related prokaryotic genomes. In the case of mutation-driven evolution, COGs and SOGs should have significantly different *dS* but approximately the same *dN*/*dS* ratios. Alternatively, if selection has been the major driver of the changes in evolutionary rates, the *dN*/*dS* ratios are expected to differ significantly between the two gene groups. Clarifying the molecular mechanisms by which prokaryotic genomes evolve may help us understand how prokaryotes develop novel functions, which is in turn relevant to ecological and biomedical studies.

Accordingly, we compared the genomes of three closely related enterobacteria [5,6]: *Escherichia coli* (ECO), *Klebsiella pneumonia* (KPN) and *Salmonella enterica* serovar Typhimurium (STM). A series of analyses were performed to control for potential confounding factors, including gene essentiality, expression level, background mutation rate, the pattern of replication-transcription confrontation, and codon usage bias. These potential confounding factors have been reported to be associated with evolutionary rates. For example, highly expressed genes, essential genes, and genes with large codon usage bias tend to evolve slowly [7-9]. Meanwhile, a higher mutation rate was observed in the genes near *Ter*[*10*] and the genes that were subject to orientation conflicts between DNA replication and transcription [3]. Our results suggest that COGs have significantly higher *dN* and *dS* than SOGs. Such increases are independent of the analyzed confounding factors, and are mainly mutation-driven.

## Methods

### Data sources

The complete genomic sequences and gene annotations of *Escherichia coli* K-12 MG 1655 (GenBank accession number U00096), *Klebsiella pneumonia* MGH 78578 (CP000647) and *Salmonella enterica* serovar Typhimurium LT2 (AE006468) were retrieved from the GenBank [11]. These species were selected because their genomes had been completely sequenced and carefully annotated, and because genome-scale gene expression data were available for these species. The orthologous gene pairs were identified according to reciprocal BLASTP best matches with the following parameters: (1) E value < 1.0 x 10^-5^ ; and (2) > 50% amino acid sequence identity. Only the orthologous genes that were found in all of the three compared species were retained. A total of 2,574 one-to-one orthologous gene groups were therefore obtained for subsequent analyses. Since one of our analyses measured the evolutionary rates separately for the terminal and central regions for individual genes, the analyzed genes must be sufficiently long to reduce variations in evolutionary rate estimates. A previous study suggested that the first 50 and the last 20 codons usually evolved more slowly than the rest [12,13]. Therefore, we took twice the length of 70 (2*(50+20) = 140) to include a central region with a minimal length of 70 codons. Accordingly, genes that are shorter than 420 bp (140*3) were discarded. Note that this practice will lead to differences in the number of analyzed gene pairs between different pair-wise species comparisons. The numbers of analyzed gene pairs are listed in Additional file 1. Since the reciprocal-BLASTP approach may not be an optimal solution for finding orthologous genes, we also retrieved orthologous genes for the analyzed species from the OMA database for comparison [14]. In fact, over 98% of the orthologous gene pairs retrieved from OMA were identical to those identified by using reciprocal BLASTP matches (see Additional file 2). We actually observed similar results when these 98% of orthologous genes were analyzed (i.e. COGs have significantly higher *dS* and *dN* but similar *dN*/*dS* when compared with SOGs; all *p*<*0.05*, see Additional file 3).

The locations of *Ori* and *Ter* were determined by using the program Oriloc [15].

### Estimation of evolutionary rates and statistical analyses

The amino acid sequences of orthologous gene pairs were aligned by using ClustalW 2.0 [16] and back-translated to nucleotide sequences. *dS* and *dN* were estimated by using the CODEML module of PAML 4.1 [17]. Only orthologous gene pairs with a *dS* value of < 3 were considered [18]. Note that this will also lead to differences in the number of analyzed genes in different pair-wise species comparisons. In the end, 1,784, 1,809 and 2,069 orthologous gene pairs were obtained, respectively, for the ECO-KPN, STM-KPN, and ECO-STM comparison (Additional file 1). Since *dS* > 3 may indicate an extremely high mutation rate and loss of function, we also investigated whether COGs tend to have *dS* > 3. Indeed, the proportion of COGs with *dS* > 3 is higher than that of all of the analyzed genes (see Additional file 4). This observation is in fact consistent with our results that COGs tend to evolve faster than SOGs.

Spearman’s rank correlations and partial correlations between evolutionary rates and the rate-determining factors were performed by using the R program (http://www.r-project.org). The statistical significance of the evolutionary rate differences was evaluated by using the Wilcoxon Rank Sum test throughout the study.

### Analysis of gene expression data

The genome-scale gene expression data of ECO (GSE15534) and STM (GSE11486) were downloaded from the Gene Expression Omnibus (GEO) database [19]. The gene expression data of KPN were generated by using a custom-made NimbleGen tiling array (provided by Dr. Bernhard O. Palsson at University of California, San Diego). In all of the three data sets, the gene expression levels were measured separately at the log and stationary phase. The signal intensity of gene expression was log2-transformed and normalized by using quantile normalization. For cross-species comparisons, the gene expression levels for each species were standardized to a median value of 0 and a variance of 1.

### Functional enrichment analysis

Gene Ontology (GO) enrichment analysis was performed by using the DAVID Bioinformatics Resources [20,21]. Since the genes of ECO are more extensively studied than those of the other two species, only the COGs in ECO-STM and ECO-KPN comparisons were included in this analysis. The gene clustering and GO term enrichment were assessed with reference to the enrichment score and the *p*-values of the modified Fisher’s Exact test.

## Results and discussion

### COGs have higher *dN* and *dS* but similar *dN*/*dS* ratio when compared with SOGs

**Figure 1:**
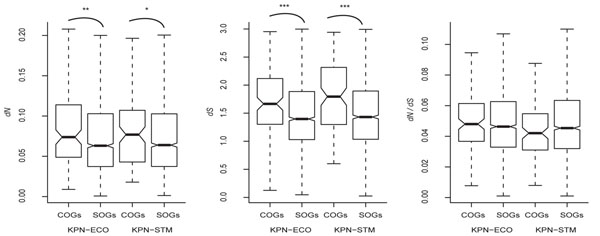
**The evolutionary rates of COGs and SOGs in the ECO-KPN and STM-KPN comparisons.** *: *p*-value < 0.05; **: *p*-value < 0.01; ***: *p*-value < 0.0001

In all the pair-wise species comparisons (ECO-KPN, STM-KPN, and ECO-STM), the COGs have significantly higher *dS* and *dN* than SOGs (all *p*<*0.05*). However, the *dN*/*dS* ratio does not show significant difference between COGs and SOGs in any of the pair-wise comparisons (Figure 1). We also used a three-way (ECO-STM-KPN) comparison to infer lineage-specific COGs, and “completely conserved SOGs” (i.e. genes that never changed transcriptional orientation among the three compared species). As shown in Additional file 5, lineage-specific (ECO-only and STM-only) COGs have significantly higher *dS* and similar *dN*/*dS* ratios when compared with completely conserved SOGs. These observations suggest that the increases in evolutionary rates in COGs are mainly mutation-driven. However, a number of confounding factors need to be excluded to further confirm these results. The confounding factors to be controlled include gene essentiality, gene expression level, background mutation rate, the transcription-replication confrontation, and codon usage bias.

### Confounding factor 1: gene essentiality

Essential genes are known to be more evolutionarily conserved than nonessential genes [8]. It is therefore necessary to clarify whether COGs tend to be non-essential, so that they evolve faster than SOGs. We thus retrieved the gene essentiality information of *E.coli*[*22*], and divided the orthologous genes into essential and nonessential genes in the ECO-KPN and ECO-STM comparisons. Our results indicate that *dN* and *dS* are significantly higher for COGs than for SOGs in nonessential genes (Table 1). We observed similar trends in essential genes, although the differences in *dN* and *dS* are statistically insignificant possibly due to the small sample sizes and the reduced variations in evolutionary rates in this gene group. Meanwhile, the *dN*/*dS* ratios are similar between COGs and SOGs, consistent with our previous observations. Therefore, the difference in the level of gene essentiality does not seem to affect our result. This is expected if the COG-SOG differences in evolutionary rates are mainly mutation-driven.

**Table 1:** The median evolutionary rates of COGs and SOGs when different confounding factors are controlled.

confounding factors	comparison	#gene (COG/SOG)	*dN*			*dS*			*dN*/*dS*		
			
			COGs	SOGs	p-value	COGs	SOGs	p-value	COGs	SOGs	p-value
nonessential gene											
	ECO-KPN	121/1426	0.076	0.067	*	1.6811	1.466	***	0.0481	0.0463	-
	ECO-STM	240/1582	0.057	0.045	***	1.5921	1.1926	***	0.0395	0.0381	-
essential gene											
	ECO-KPN	8/224	0.045	0.041	-	1.374	0.978	-	0.042	0.046	-
	ECO-STM	17/225	0.025	0.025	-	0.785	0.825	-	0.029	0.032	-
non-highly expressed gene											
	ECO-KPN (log phase)	106/1240	0.082	0.074	-	1.721	1.561	**	0.049	0,047	-
	ECO-KPN (stationary phase)	103/1342	0.077	0.066	-	1.686	1.457	***	0.048	0.046	-
	STM-KPN (log phase)	88/1416	0.077	0.068	-	1.845	1,507	***	0.042	0.045	-
	STM-KPN (stationary phase)	80/1475	0.077	0.065	-	1.77	1.458	***	0.042	0.046	-
highly expressed											
	ECO-KPN (log phase)	23/415	0.045	0.037	-	1.352	0.896	**	0.042	0.044	-
	ECO-KPN (stationary phase)	26/313	0.064	0.047	-	1.459	1.142	*	0.047	0.045	-
	STM-KPN (log phase)	18/287	0.06	0.037	-	1.384	0.895	*	0.046	0.046	-
	STM-KPN (stationary phase)	26/228	0.075	0.053	*	1.845	1.273	***	0.042	0.044	-

* : p < 0.05: **: p < 0.01: ***: p < 0.001; -: insignificant

### Confounding factor 2: gene expression level

Highly expressed genes are under strong selection pressure to maintain functional stability, and thus usually evolve more slowly than lowly expressed genes [23,24]. The question now is whether SOGs tend to be highly expressed, so that they evolve more slowly than COGs. We thus classified the analyzed genes into highly (top 20%) and non-highly (other 80%) expressed genes. In the ECO-KPN and STM-KPN comparisons, the *dS* of COGs is significantly larger than SOGs in both highly and non-highly expressed genes for both growth phases (stationary and log phase, Table 1). *dN* values also show similar trends. However, the *dN*/*dS* ratios are approximately the same between COGs and SOGs (Table 1). Notably, it has been previously reported that mutation rate may increase with gene expression level [25-27]. Therefore, it is of interest to investigate whether the increases in evolutionary rate in COGs are associated with increased expression levels in these genes. However, our results indicate that this is not the case. Highly expressed COGs actually have lower *dN* and *dS* than non-highly expressed COGs (Additional file 6). Therefore, the increases in evolutionary rates (particularly *dS*) in COGs do not result from increased expression levels in these genes.

### Confounding factor 3: background mutation rate

**Figure 2:**
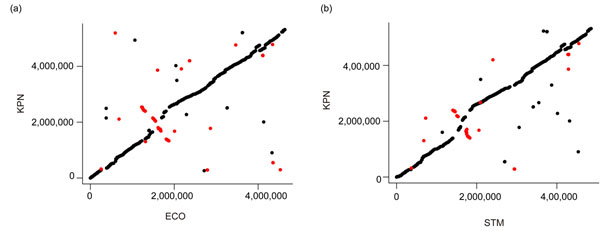
**Dot plot for the analyzed orthologous genes in the (a) ECO-KPN; and (b) STM-KPN comparison.** The Y and X axis represents, respectively, the chromosomal positions of the KPN genes (in base pair) and those of the orthologous genes in ECO (left panel) or STM (right panel). The black and red dots represent SOGs and COGs, respectively. Most of the COGs are located close to *Ter*. The positions of *Ter* are 1,892,000, 1,549,000 and 1,635,000 bp for KPN, ECO and STM, respectively.

Although gene inversions usually occur in the vicinity of both *Ori* and *Ter*[*1*,*2*], we actually found that most COGs analyzed in this study were located near *Ter* (Figure 2). It is known that the genes located near *Ter* have an increased mutation rate [28]. We then ask whether the uneven chromosomal distribution of COGs has contributed to the COG-SOG differences in evolutionary rates. Therefore, we performed partial correlation analyses to examine the relationship between evolutionary rates and gene type (COG or SOG). Our results indicate that *dS* is significantly correlated with the COG/SOG gene type even when the distance effect is controlled (Spearman’s *ρ* > 0.05 and *p* < 0.05) (Additional file 7). The positive correlation indicates that CTOs have a location-independent effect on the increase of *dS*. Meanwhile, the distance-controlled correlations are statistically insignificant between *dN* (and also *dN*/*dS*) and the COG/SOG gene type. Together, these results indicate that CTOs actually have a location-independent effect on *dS*. Meanwhile, the increase in *dN* in COGs is likely affected by the chromosomal locations of these genes. However, since the COGs that are close to *Ter* far outnumber those that are not, more evidence is needed to clarify the correlation between *dN* and the COG/SOG gene type in the context of background mutation rate.

### Confounding factor 4: replication–transcription confrontation

The head-on collision between the directions of transcription and replication has been reported to increase the mutation rate of the affected genes [3,10,29]. When a gene is involved in an inversion event, its transcriptional orientation is likely to change, causing replication-transcription confrontation. We thus investigated whether COGs have replication-transcription confrontation more often than SOGs. Interestingly, most of the COGs maintain their status in terms of replication-transcription confrontation. For example, using KPN as an outgroup, we found that 157 of 162 COGs in the ECO-STM comparison maintain the status of head-on collision or co-orientation between replication and transcription after the occurrences of the inversion events. Meanwhile, the ratio of head-on-collision to co-orientation genes in COGs and SOGs are 1.1 and 0.64, respectively in the ECO–STM comparison. Therefore, COGs are in fact more likely to be head-on-collision genes than SOGs. To further clarify whether the difference in the degree of head-on collision is a major determinant of the COG-SOG differences in evolutionary rate, we compared the evolutionary rates of COGs and SOGs by controlling the replication-transcription confrontation pattern. In the ECO-STM comparison, the rates of *dN*, *dS* and *dN*/*dS* of head-on-collision COGs do not differ significantly from co-orientation COGs (Additional File 8). By contrast, head-on-collision SOGs have significantly higher evolutionary rates than co-orientation SOGs. These results suggest that the higher evolutionary rates of COGs do not result from a higher proportion of head-on-collision genes. Rather, CTO by itself may be the major reason for the increased evolutionary rate.

### Confounding factor 5: decreased *dS* at gene terminals (codon usage bias)

**Figure 3:**
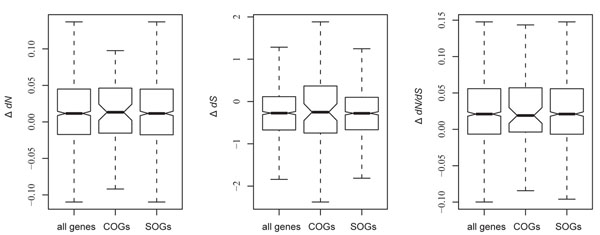
**Difference in evolutionary rates between the terminal and middle region of ECO-KPN orthologous genes.** The Y axis shows the difference (terminal minus middle) in *dN*, *dS*, and *dN*/*dS* ratio, respectively, for the left, middle, and right panel. The differences in *dN*, *dS* and *dN*/*dS* ratio are statistically insignificant between any pair-wise gene group comparisons in each panel.

It has been reported that the synonymous substitution rate decreases near the first 50-100 codons [12] and the last 20 codons of a gene [13], possibly because codon usage is less biased at these regions. We then ask whether SOGs tend to have lowered *dS* at both ends as compared with COGs, so that the overall *dS* is decreased. Accordingly, we divided each of the analyzed genes into terminal (first 50 and last 20 codons) and middle region (the rest of the codons). We then randomly sampled 70 codons from the middle region for comparison. The differences in evolutionary rates (Δ*dS*, Δ*dN*, and Δ*dN*/*dS*) between the terminal codons and the randomly sampled codons were calculated and compared between COGs and SOGs. Our results show that Δ*dS*, Δ*dN*, and Δ*dN*/*dS* are similar between COGs and SOGs (Figure 3 for ECO-KPN comparison and Additional file 9 for STM-KPN comparison; all of the pair-wise differences are statistically insignificant). Therefore, the difference in codon usage bias in evolutionary rate does not appear to affect our overall results.

### Functional enrichment analysis of COGs

We next ask whether the rapidly evolving COGs are enriched in certain functional categories. Additional file 10 indicates that the protein products of COGs tend to be located on membranes and cell walls, and be involved in a variety of metabolic reactions. This observation is biologically sensible because (1) proteins that are located on the cell surface tend to evolve faster; and (2) different bacterial species have very different metabolic capacities. However, the relationship between CTOs and the functional preferences of these genes remains unknown.

## Conclusions

To our knowledge, this is the first study to demonstrate that changes in transcriptional orientation may increase the evolutionary rates (*dN* and *dS*) of prokaryotic genes. We show that the evolutionary effects of CTOs are independent of gene essentiality, gene expression level, replication-transcriptional confrontation, or the decrease in *dS* at gene terminals. However, the increase in *dN* may be partially related to gene locations. Furthermore, our results suggest that the increases in evolutionary rates in COGs are mainly mutation-driven, as the *dN*/*dS* ratios are similar between COGs and SOGs. The real cause of the increases in evolutionary rate in COGs (particularly *dS*) remains unclear but is worth further explorations. It is speculated that CTOs may somehow result in impediments in DNA replication (by altering, for example, DNA protein binding sites or secondary structure), which may in turn lead to the recruitment of error-prone DNA repair polymerases and the increase in mutation rate [30,31].

## Competing interests

The authors declare that they have no competing interests.

## Authors' contributions

CHL conceived the study and carried out the data analyses. CHL, FCC and CAH designed the conceptual framework of the study, interpreted the results, and drafted the manuscript. CYL pre-processed gene expression data and assisted in the statistical analyses.

## Supplementary Material

Additional file 1 The numbers and detailed information of the orthologous gene pairs analyzed in this study. Click here for file

Additional file 2 The numbers of ECO-KPN, ECO-STM, and STM-KPN orthologous gene pairs identified by reciprocal BLASTP and the OMA database Click here for file

Additional file 3 The evolutionary rates of COGs and SOGs in the ECO-KPN and STM-KPN comparisons. The genes included in this analysis were identified by both reciprocal BLASTP and OMA database as orthologous genes. *: *p*-value < 0.05; **: *p*-value < 0.01; ***: *p*-value < 0.0001 Click here for file

Additional file 4 The numbers of COGs with *dS* > 3 in the ECO-KPN, ECO-STM, and STM-KPN comparisons. Click here for file

Additional file 5 The evolutionary rates in (a) ECO-KPN comparison; and (b) STM-KPN comparison. Here we compare the evolutionary rates of the genes that never changed transcriptional orientation in ECO, STM, and KPN, (“ESK”), the genes that changed orientation in either the ECO-STM lineage or in the KPN lineage (“ES/K”), and the genes that changed orientation in only one species (“ECO-only” or “STM-only”). Click here for file

Additional file 6 Comparison of the evolutionary rates of highly and non-highly expressed COGs. (a) ECO-KPN comparison at the log phase; (b) ECO-KPN comparison at the stationary phase; (c) STM-KPN comparison at the log phase; (d) STM-KPN comparison at the stationary phase. *: *p*-value < 0.05; **: *p*-value < 0.01 Click here for file

Additional file 7 Partial correlations between evolutionary rates and the COG/SOG gene type while the physical distance from *Ter* is controlled. Click here for file

Additional file 8 The evolutionary rates of COGs and SOGs that are subject to head-on collision or co-orientation between DNA replication and transcription in the ECO-STM comparison. * represents *p* value < 0.05. Click here for file

Additional file 9 Comparison of Δ*dN*, Δ*dS* and Δ*dN*/*dS* between COGs and SOGs in the STM-KPN comparison. Δ*dN*, Δ*dS* and Δ*dN*/*dS* were calculated by subtracting the evolutionary rates of the middle region from those of the terminal regions of each gene. Note that none of the pair-wise comparisons in any of the three panels is statistically significant. Click here for file

Additional file 10 Functional enrichment analyses for COGs in the (a) ECO-KPN and (b) ECO-STM comparisons. Note that the functional assignments were based on the gene annotations for ECO. Click here for file
