# Supplementary material for: Changes in transcriptional orientation are associated with increases in evolutionary rates of enterobacterial genes
Source: BMC Bioinformatics. 2011 Oct 5;12(Suppl 9):S19. doi: 10.1186/1471-2105-12-S9-S19 (PMC3283321; doi:10.1186/1471-2105-12-S9-S19)
Supplement: Additional file 1 — The numbers and detailed information of the orthologous gene pairs analyzed in this study. [file 1471-2105-12-S9-S19-S1.pdf]

The numbers of orthologous gene pairs at each filtering step

Filter steps	(1) one-to-one orthologs	(2) control for length (> 420 Bp)	(3) control for *dS* (*dS* < 3)			
			all	COG	SOG	% COG
ECO-KPN	2574	2169	1784	129	1655	7.23%
ECO-STM	2574	2172	2069	257	1812	12.42%
STM-KPN	2574	2167	1809	106	1703	5.86%

ECO: *Escherichia coli* ; STM: *Salmonella typhimurium* ; KPN: *Klebsiella pneumonia*

Orthologous gene pair in ECO-KPN comparison

ECO	KPN	Type
b0002	KPN_00002	SOG
b0003	KPN_00003	SOG
b0004	KPN_00004	SOG
b0006	KPN_00006	SOG
b0008	KPN_00008	SOG
b0009	KPN_00009	SOG
b0010	KPN_00011	SOG
b0011	KPN_00012	SOG
b0014	KPN_00014	SOG
b0015	KPN_00015	SOG
b0020	KPN_00017	SOG
b0025	KPN_00020	SOG
b0026	KPN_00021	SOG
b0027	KPN_00022	SOG
b0028	KPN_00023	SOG
b0029	KPN_00024	SOG
b0030	KPN_00025	SOG
b0031	KPN_00039	SOG
b0032	KPN_00040	SOG
b0033	KPN_00041	SOG
b0046	KPN_00043	SOG
b0047	KPN_00044	SOG
b0048	KPN_00045	SOG
b0049	KPN_00046	SOG
b0051	KPN_00048	SOG
b0052	KPN_00049	SOG
b0053	KPN_00050	SOG
b0054	KPN_00051	SOG
b0058	KPN_00057	SOG
b0059	KPN_00058	SOG
b0060	KPN_00059	SOG
b0061	KPN_00060	SOG
b0062	KPN_00061	SOG
b0063	KPN_00062	SOG
b0064	KPN_00063	SOG
b0065	KPN_00064	SOG
b0066	KPN_00065	SOG
b0067	KPN_00066	SOG
b0068	KPN_00067	SOG
b0069	KPN_00068	SOG
b0071	KPN_00077	SOG
b0072	KPN_00078	SOG
b0073	KPN_00079	SOG

Orthologous gene pair in ECO-STM comparison

ECO	STM	Type
b0002	STM0002	SOG
b0003	STM0003	SOG
b0004	STM0004	SOG
b0006	STM0005	SOG
b0007	STM0006	SOG
b0008	STM0007	SOG
b0009	STM0008	SOG
b0010	STM0009	SOG
b0011	STM0010	SOG
b0014	STM0012	SOG
b0015	STM0013	SOG
b0019	STM0039	SOG
b0020	STM0040	SOG
b0025	STM0045	SOG
b0026	STM0046	SOG
b0027	STM0047	SOG
b0028	STM0048	SOG
b0029	STM0049	SOG
b0030	STM0051	SOG
b0031	STM0064	SOG
b0032	STM0066	SOG
b0033	STM0067	SOG
b0046	STM0085	SOG
b0047	STM0086	SOG
b0048	STM0087	SOG
b0049	STM0088	SOG
b0051	STM0090	SOG
b0052	STM0091	SOG
b0053	STM0092	SOG
b0054	STM0093	SOG
b0055	STM0094	SOG
b0058	STM0095	SOG
b0059	STM0096	SOG
b0060	STM0097	SOG
b0061	STM0101	SOG
b0062	STM0102	SOG
b0063	STM0103	SOG
b0064	STM0104	SOG
b0065	STM0105	SOG
b0066	STM0106	SOG
b0067	STM0107	SOG
b0068	STM0108	SOG
b0069	STM0109	SOG

Orthologous gene pair in STM-KPN comparison

STM	KPN	Type
STM0002	KPN_00002	SOG
STM0003	KPN_00003	SOG
STM0004	KPN_00004	SOG
STM0005	KPN_00006	SOG
STM0007	KPN_00008	SOG
STM0008	KPN_00009	SOG
STM0009	KPN_00011	SOG
STM0012	KPN_00014	SOG
STM0013	KPN_00015	SOG
STM0040	KPN_00017	SOG
STM0045	KPN_00020	SOG
STM0046	KPN_00021	SOG
STM0047	KPN_00022	SOG
STM0048	KPN_00023	SOG
STM0049	KPN_00024	SOG
STM0064	KPN_00039	SOG
STM0066	KPN_00040	SOG
STM0067	KPN_00041	SOG
STM0085	KPN_00043	SOG
STM0086	KPN_00044	SOG
STM0087	KPN_00045	SOG
STM0088	KPN_00046	SOG
STM0090	KPN_00048	SOG
STM0091	KPN_00049	SOG
STM0092	KPN_00050	SOG
STM0093	KPN_00051	SOG
STM0094	KPN_00052	SOG
STM0095	KPN_00057	SOG
STM0096	KPN_00058	SOG
STM0097	KPN_00059	SOG
STM0101	KPN_00060	SOG
STM0102	KPN_00061	SOG
STM0103	KPN_00062	SOG
STM0104	KPN_00063	SOG
STM0105	KPN_00064	SOG
STM0106	KPN_00065	SOG
STM0107	KPN_00066	SOG
STM0108	KPN_00067	SOG
STM0109	KPN_00068	SOG
STM0111	KPN_00078	SOG
STM0112	KPN_00079	SOG
STM0113	KPN_00080	SOG
STM0115	KPN_00081	SOG

b0074	KPN_00080	SOG
b0076	KPN_00081	SOG
b0077	KPN_00082	SOG
b0078	KPN_00083	SOG
b0080	KPN_00084	SOG
b0081	KPN_00085	SOG
b0082	KPN_00086	SOG
b0084	KPN_00088	SOG
b0085	KPN_00089	SOG
b0086	KPN_00090	SOG
b0087	KPN_00091	SOG
b0088	KPN_00092	SOG
b0089	KPN_00093	SOG
b0090	KPN_00094	SOG
b0091	KPN_00095	SOG
b0092	KPN_00096	SOG
b0093	KPN_00097	SOG
b0094	KPN_00098	SOG
b0095	KPN_00099	SOG
b0096	KPN_00100	SOG
b0097	KPN_00101	SOG
b0098	KPN_00102	SOG
b0102	KPN_00105	SOG
b0103	KPN_00106	SOG
b0104	KPN_00107	SOG
b0109	KPN_00111	SOG
b0110	KPN_00112	SOG
b0111	KPN_00113	SOG
b0112	KPN_00116	SOG
b0113	KPN_00117	SOG
b0114	KPN_00118	SOG
b0115	KPN_00119	SOG
b0116	KPN_00120	SOG
b0118	KPN_00124	SOG
b0120	KPN_00126	SOG
b0121	KPN_00127	SOG
b0123	KPN_00131	SOG
b0124	KPN_00132	SOG
b0125	KPN_00133	SOG
b0126	KPN_00134	SOG
b0127	KPN_00135	SOG
b0128	KPN_00136	SOG
b0130	KPN_00138	SOG
b0133	KPN_00140	SOG
b0134	KPN_00141	SOG
b0142	KPN_00142	SOG

b0071	STM0110	SOG
b0072	STM0111	SOG
b0073	STM0112	SOG
b0074	STM0113	SOG
b0076	STM0115	SOG
b0077	STM0116	SOG
b0078	STM0117	SOG
b0080	STM0118	SOG
b0081	STM0119	SOG
b0082	STM0120	SOG
b0084	STM0122	SOG
b0085	STM0123	SOG
b0086	STM0124	SOG
b0087	STM0125	SOG
b0088	STM0126	SOG
b0089	STM0127	SOG
b0090	STM0128	SOG
b0091	STM0129	SOG
b0092	STM0130	SOG
b0093	STM0131	SOG
b0094	STM0132	SOG
b0095	STM0133	SOG
b0096	STM0134	SOG
b0097	STM0135	SOG
b0098	STM0136	SOG
b0102	STM0139	SOG
b0103	STM0140	SOG
b0104	STM0141	SOG
b0106	STM0142	SOG
b0107	STM0143	SOG
b0108	STM0144	SOG
b0109	STM0145	SOG
b0110	STM0146	SOG
b0111	STM0147	SOG
b0112	STM0150	SOG
b0113	STM0151	SOG
b0114	STM0152	SOG
b0115	STM0153	SOG
b0116	STM0154	SOG
b0117	STM0157	SOG
b0118	STM0158	SOG
b0120	STM0165	SOG
b0121	STM0166	SOG
b0123	STM0168	SOG
b0124	STM0169	SOG
b0125	STM0170	SOG

STM0116	KPN_00082	SOG
STM0117	KPN_00083	SOG
STM0118	KPN_00084	SOG
STM0119	KPN_00085	SOG
STM0120	KPN_00086	SOG
STM0122	KPN_00088	SOG
STM0123	KPN_00089	SOG
STM0124	KPN_00090	SOG
STM0125	KPN_00091	SOG
STM0126	KPN_00092	SOG
STM0127	KPN_00093	SOG
STM0128	KPN_00094	SOG
STM0129	KPN_00095	SOG
STM0130	KPN_00096	SOG
STM0131	KPN_00097	SOG
STM0132	KPN_00098	SOG
STM0133	KPN_00099	SOG
STM0134	KPN_00100	SOG
STM0135	KPN_00101	SOG
STM0136	KPN_00102	SOG
STM0139	KPN_00105	SOG
STM0140	KPN_00106	SOG
STM0141	KPN_00107	SOG
STM0144	KPN_00110	SOG
STM0145	KPN_00111	SOG
STM0146	KPN_00112	SOG
STM0147	KPN_00113	SOG
STM0150	KPN_00116	SOG
STM0151	KPN_00117	SOG
STM0152	KPN_00118	SOG
STM0153	KPN_00119	SOG
STM0154	KPN_00120	SOG
STM0158	KPN_00124	SOG
STM0165	KPN_00126	SOG
STM0166	KPN_00127	SOG
STM0169	KPN_00132	SOG
STM0170	KPN_00133	SOG
STM0171	KPN_00134	SOG
STM0172	KPN_00135	SOG
STM0173	KPN_00136	SOG
STM0178	KPN_00137	SOG
STM0179	KPN_00138	SOG
STM0181	KPN_00140	SOG
STM0182	KPN_00141	SOG
STM0184	KPN_00143	SOG
STM0186	KPN_00145	SOG

b0143	KPN_00143	SOG
b0144	KPN_00144	SOG
b0145	KPN_00145	SOG
b0146	KPN_00146	SOG
b0148	KPN_00148	SOG
b0149	KPN_00164	SOG
b0150	KPN_00165	SOG
b0151	KPN_00166	SOG
b0152	KPN_00167	SOG
b0153	KPN_00168	SOG
b0154	KPN_00169	SOG
b0155	KPN_00170	SOG
b0157	KPN_00172	SOG
b0158	KPN_00173	SOG
b0159	KPN_00174	SOG
b0160	KPN_00175	SOG
b0161	KPN_00176	SOG
b0162	KPN_00177	SOG
b0166	KPN_00179	SOG
b0167	KPN_00180	SOG
b0168	KPN_00181	SOG
b0169	KPN_00182	SOG
b0170	KPN_00183	SOG
b0171	KPN_00184	SOG
b0172	KPN_00185	SOG
b0174	KPN_00187	SOG
b0175	KPN_00188	SOG
b0176	KPN_00189	SOG
b0177	KPN_00190	SOG
b0178	KPN_00191	SOG
b0179	KPN_00192	SOG
b0180	KPN_00193	SOG
b0181	KPN_00194	SOG
b0182	KPN_00195	SOG
b0183	KPN_00196	SOG
b0184	KPN_00197	SOG
b0185	KPN_00198	SOG
b0186	KPN_00199	SOG
b0190	KPN_00204	SOG
b0194	KPN_00208	SOG
b0195	KPN_00209	SOG
b0197	KPN_00211	SOG
b0198	KPN_00212	SOG
b0199	KPN_00213	SOG
b0200	KPN_00214	SOG
b0207	KPN_00221	SOG

b0126	STM0171	SOG
b0127	STM0172	SOG
b0128	STM0173	SOG
b0129	STM0178	SOG
b0130	STM0179	SOG
b0133	STM0181	SOG
b0134	STM0182	SOG
b0142	STM0183	SOG
b0143	STM0184	SOG
b0145	STM0186	SOG
b0146	STM0187	SOG
b0147	STM0188	SOG
b0148	STM0189	SOG
b0149	STM0190	SOG
b0150	STM0191	SOG
b0151	STM0192	SOG
b0152	STM0193	SOG
b0153	STM0194	SOG
b0154	STM0202	SOG
b0155	STM0203	SOG
b0157	STM0205	SOG
b0158	STM0206	SOG
b0159	STM0207	SOG
b0160	STM0208	SOG
b0161	STM0209	SOG
b0162	STM0210	SOG
b0166	STM0213	SOG
b0167	STM0214	SOG
b0168	STM0215	SOG
b0169	STM0216	SOG
b0170	STM0217	SOG
b0171	STM0218	SOG
b0172	STM0219	SOG
b0173	STM0220	SOG
b0174	STM0221	SOG
b0175	STM0222	SOG
b0176	STM0223	SOG
b0177	STM0224	SOG
b0178	STM0225	SOG
b0179	STM0226	SOG
b0180	STM0227	SOG
b0181	STM0228	SOG
b0182	STM0229	SOG
b0183	STM0230	SOG
b0184	STM0231	SOG
b0185	STM0232	SOG

STM0187	KPN_00146	SOG
STM0190	KPN_00164	SOG
STM0191	KPN_00165	SOG
STM0192	KPN_00166	SOG
STM0194	KPN_00168	SOG
STM0202	KPN_00169	SOG
STM0203	KPN_00170	SOG
STM0205	KPN_00172	SOG
STM0206	KPN_00173	SOG
STM0207	KPN_00174	SOG
STM0208	KPN_00175	SOG
STM0209	KPN_00176	SOG
STM0210	KPN_00177	SOG
STM0213	KPN_00179	SOG
STM0214	KPN_00180	SOG
STM0215	KPN_00181	SOG
STM0216	KPN_00182	SOG
STM0217	KPN_00183	SOG
STM0218	KPN_00184	SOG
STM0219	KPN_00185	SOG
STM0221	KPN_00187	SOG
STM0222	KPN_00188	SOG
STM0223	KPN_00189	SOG
STM0224	KPN_00190	SOG
STM0225	KPN_00191	SOG
STM0226	KPN_00192	SOG
STM0227	KPN_00193	SOG
STM0228	KPN_00194	SOG
STM0229	KPN_00195	SOG
STM0230	KPN_00196	SOG
STM0231	KPN_00197	SOG
STM0232	KPN_00198	SOG
STM0234	KPN_00199	SOG
STM0239	KPN_00204	SOG
STM0242	KPN_00208	SOG
STM0243	KPN_00209	SOG
STM0245	KPN_00211	SOG
STM0246	KPN_00212	SOG
STM0247	KPN_00213	SOG
STM0248	KPN_00214	SOG
STM0255	KPN_00221	SOG
STM0256	KPN_00222	SOG
STM0258	KPN_00224	SOG
STM0260	KPN_00226	SOG
STM0261	KPN_00227	SOG
STM0262	KPN_00228	SOG

b0208	KPN_00222	SOG
b0209	KPN_00224	SOG
b0210	KPN_00225	SOG
b0211	KPN_00226	SOG
b0212	KPN_00227	SOG
b0213	KPN_00228	SOG
b0214	KPN_00229	SOG
b0215	KPN_00230	SOG
b2577	KPN_00232	SOG
b0219	KPN_00234	SOG
b0221	KPN_00235	SOG
b0222	KPN_00236	SOG
b0223	KPN_00237	SOG
b0224	KPN_00238	SOG
b0231	KPN_00247	SOG
b0237	KPN_00249	SOG
b0238	KPN_00250	SOG
b0239	KPN_00251	SOG
b2663	KPN_00254	COG
b2662	KPN_00255	COG
b2661	KPN_00256	COG
b2660	KPN_00257	COG
b2659	KPN_00258	COG
b4306	KPN_00260	COG
b0243	KPN_00280	COG
b0242	KPN_00281	COG
b0241	KPN_00282	COG
b0369	KPN_00316	SOG
b0376	KPN_00317	SOG
b0377	KPN_00319	SOG
b0378	KPN_00320	SOG
b0381	KPN_00323	SOG
b0386	KPN_00329	SOG
b0387	KPN_00330	SOG
b0388	KPN_00332	SOG
b0393	KPN_00336	SOG
b0394	KPN_00337	SOG
b0398	KPN_00339	SOG
b0399	KPN_00340	SOG
b0400	KPN_00341	SOG
b0401	KPN_00342	SOG
b0402	KPN_00343	SOG
b0403	KPN_00344	SOG
b0405	KPN_00356	SOG
b0406	KPN_00357	SOG
b0408	KPN_00359	SOG

b0186	STM0234	SOG
b0188	STM0236	SOG
b0190	STM0239	SOG
b0191	STM0240	SOG
b0194	STM0242	SOG
b0195	STM0243	SOG
b0197	STM0245	SOG
b0198	STM0246	SOG
b0199	STM0247	SOG
b0200	STM0248	SOG
b0207	STM0255	SOG
b0208	STM0256	SOG
b0209	STM0258	SOG
b0210	STM0259	SOG
b0211	STM0260	SOG
b0212	STM0261	SOG
b0213	STM0262	SOG
b0214	STM0263	SOG
b0215	STM0264	SOG
b0219	STM0308	SOG
b0221	STM0309	SOG
b0222	STM0310	SOG
b0223	STM0311	SOG
b0224	STM0312	SOG
b0231	STM0313	SOG
b0237	STM0316	SOG
b0238	STM0317	SOG
b0239	STM0318	SOG
b0242	STM0321	SOG
b0243	STM0322	SOG
b0337	STM3334	SOG
b0354	STM4192	SOG
b0356	STM1627	SOG
b0369	STM0372	SOG
b0376	STM0375	SOG
b0377	STM0376	SOG
b0378	STM0377	SOG
b0381	STM0380	SOG
b0386	STM0386	SOG
b0387	STM0387	SOG
b0388	STM0388	SOG
b0390	STM0390	SOG
b0393	STM0392	SOG
b0394	STM0393	SOG
b0396	STM0394	SOG
b0397	STM0395	SOG

STM0263	KPN_00229	SOG
STM0264	KPN_00230	SOG
STM0308	KPN_00234	SOG
STM0309	KPN_00235	SOG
STM0310	KPN_00236	SOG
STM0311	KPN_00237	SOG
STM0312	KPN_00238	SOG
STM0313	KPN_00247	SOG
STM0316	KPN_00249	SOG
STM0317	KPN_00250	SOG
STM0318	KPN_00251	SOG
STM2794	KPN_00253	COG
STM2793	KPN_00254	COG
STM2792	KPN_00255	COG
STM2791	KPN_00256	COG
STM2790	KPN_00257	COG
STM2789	KPN_00258	COG
STM0322	KPN_00280	COG
STM0321	KPN_00281	COG
STM0320	KPN_00282	COG
STM0372	KPN_00316	SOG
STM0375	KPN_00317	SOG
STM0376	KPN_00319	SOG
STM0377	KPN_00320	SOG
STM0380	KPN_00323	SOG
STM0386	KPN_00329	SOG
STM0387	KPN_00330	SOG
STM0388	KPN_00332	SOG
STM0392	KPN_00336	SOG
STM0393	KPN_00337	SOG
STM0397	KPN_00340	SOG
STM0398	KPN_00341	SOG
STM0399	KPN_00342	SOG
STM0400	KPN_00343	SOG
STM0401	KPN_00344	SOG
STM0404	KPN_00356	SOG
STM0405	KPN_00357	SOG
STM0407	KPN_00359	SOG
STM0408	KPN_00360	SOG
STM0413	KPN_00363	SOG
STM0414	KPN_00364	SOG
STM0415	KPN_00365	SOG
STM0416	KPN_00366	SOG
STM0417	KPN_00367	SOG
STM0419	KPN_00369	SOG
STM0420	KPN_00370	SOG

b0409	KPN_00360	SOG
b0411	KPN_00363	SOG
b0413	KPN_00365	SOG
b0414	KPN_00366	SOG
b0415	KPN_00367	SOG
b0417	KPN_00369	SOG
b0418	KPN_00370	SOG
b0420	KPN_00372	SOG
b0421	KPN_00373	SOG
b0423	KPN_00375	SOG
b0424	KPN_00377	SOG
b0425	KPN_00378	SOG
b0426	KPN_00379	SOG
b0427	KPN_00380	SOG
b0428	KPN_00390	SOG
b0430	KPN_00392	SOG
b0431	KPN_00393	SOG
b0432	KPN_00394	SOG
b0433	KPN_00395	SOG
b0434	KPN_00396	SOG
b0436	KPN_00398	SOG
b0437	KPN_00399	SOG
b0438	KPN_00400	SOG
b0439	KPN_00401	SOG
b0441	KPN_00403	SOG
b0444	KPN_00406	SOG
b0446	KPN_00408	SOG
b0448	KPN_00411	SOG
b0449	KPN_00412	SOG
b0451	KPN_00414	SOG
b0452	KPN_00415	SOG
b0458	KPN_00439	SOG
b0462	KPN_00443	SOG
b0463	KPN_00444	SOG
b0464	KPN_00445	SOG
b0465	KPN_00446	SOG
b0469	KPN_00451	SOG
b0470	KPN_00452	SOG
b0472	KPN_00454	SOG
b0473	KPN_00455	SOG
b0474	KPN_00456	SOG
b0477	KPN_00458	SOG
b0478	KPN_00459	SOG
b0479	KPN_00460	SOG
b0480	KPN_00461	SOG
b0481	KPN_00462	SOG

b0398	STM0396	SOG
b0399	STM0397	SOG
b0400	STM0398	SOG
b0401	STM0399	SOG
b0402	STM0400	SOG
b0403	STM0401	SOG
b0404	STM0403	SOG
b0405	STM0404	SOG
b0406	STM0405	SOG
b0408	STM0407	SOG
b0409	STM0408	SOG
b0411	STM0413	SOG
b0412	STM0414	SOG
b0413	STM0415	SOG
b0414	STM0416	SOG
b0415	STM0417	SOG
b0417	STM0419	SOG
b0418	STM0420	SOG
b0419	STM0421	SOG
b0420	STM0422	SOG
b0421	STM0423	SOG
b0423	STM0425	SOG
b0424	STM0433	SOG
b0425	STM0434	SOG
b0426	STM0435	SOG
b0427	STM0436.S	SOG
b0428	STM0439	SOG
b0430	STM0441	SOG
b0431	STM0442	SOG
b0432	STM0443	SOG
b0433	STM0444	SOG
b0434	STM0445	SOG
b0436	STM0447	SOG
b0437	STM0448	SOG
b0438	STM0449	SOG
b0439	STM0450	SOG
b0441	STM0452	SOG
b0444	STM0455	SOG
b0445	STM0456	SOG
b0446	STM0457	SOG
b0447	STM0459	SOG
b0448	STM0460	SOG
b0449	STM0461	SOG
b0451	STM0463	SOG
b0452	STM0464	SOG
b0453	STM0465	SOG

STM0421	KPN_00371	SOG
STM0422	KPN_00372	SOG
STM0423	KPN_00373	SOG
STM0425	KPN_00375	SOG
STM0433	KPN_00377	SOG
STM0434	KPN_00378	SOG
STM0435	KPN_00379	SOG
STM0436.S	KPN_00380	SOG
STM0439	KPN_00390	SOG
STM0441	KPN_00392	SOG
STM0442	KPN_00393	SOG
STM0443	KPN_00394	SOG
STM0444	KPN_00395	SOG
STM0445	KPN_00396	SOG
STM0447	KPN_00398	SOG
STM0448	KPN_00399	SOG
STM0449	KPN_00400	SOG
STM0450	KPN_00401	SOG
STM0452	KPN_00403	SOG
STM0455	KPN_00406	SOG
STM0456	KPN_00407	SOG
STM0457	KPN_00408	SOG
STM0459	KPN_00410	SOG
STM0460	KPN_00411	SOG
STM0461	KPN_00412	SOG
STM0463	KPN_00414	SOG
STM0464	KPN_00415	SOG
STM0465	KPN_00416	SOG
STM0471	KPN_00439	SOG
STM0475	KPN_00443	SOG
STM0476	KPN_00444	SOG
STM0477	KPN_00445	SOG
STM0478	KPN_00446	SOG
STM0481	KPN_00449	SOG
STM0483	KPN_00451	SOG
STM0484	KPN_00452	SOG
STM0486	KPN_00454	SOG
STM0487.S	KPN_00455	SOG
STM0488	KPN_00456	SOG
STM0491	KPN_00458	SOG
STM0492	KPN_00459	SOG
STM0493	KPN_00460	SOG
STM0494	KPN_00461	SOG
STM0495	KPN_00462	SOG
STM0498	KPN_00464	SOG
STM0501	KPN_00467	SOG

b0484	KPN_00464	SOG
b0489	KPN_00467	SOG
b0491	KPN_00469	SOG
b0492	KPN_00470	SOG
b0493	KPN_00471	SOG
b0494	KPN_00472	SOG
b0495	KPN_00473	SOG
b0496	KPN_00474	SOG
b0503	KPN_00476	SOG
b0522	KPN_00477	SOG
b0523	KPN_00478	SOG
b0524	KPN_00480	SOG
b0525	KPN_00481	SOG
b0526	KPN_00482	SOG
b0529	KPN_00484	SOG
b4132	KPN_00499	COG
b4131	KPN_00500	COG
b4130	KPN_00501	COG
b0576	KPN_00537	SOG
b0577	KPN_00543	SOG
b0578	KPN_00553	SOG
b0584	KPN_00602	SOG
b0588	KPN_00606	SOG
b0589	KPN_00607	SOG
b0590	KPN_00608	SOG
b0592	KPN_00610	SOG
b0593	KPN_00611	SOG
b0594	KPN_00612	SOG
b0595	KPN_00613	SOG
b0596	KPN_00614	SOG
b0598	KPN_00616	SOG
b0600	KPN_00645	SOG
b0601	KPN_00646	SOG
b0605	KPN_00650	SOG
b0606	KPN_00651	SOG
b0621	KPN_00654	SOG
b0628	KPN_00660	SOG
b0630	KPN_00662	SOG
b0632	KPN_00664	SOG
b0633	KPN_00665	SOG
b0634	KPN_00666	SOG
b0635	KPN_00667	SOG
b0636	KPN_00668	SOG
b0638	KPN_00670	SOG
b0639	KPN_00671	SOG
b0640	KPN_00672	SOG

b0458	STM0471	SOG
b0459	STM0472	SOG
b0462	STM0475	SOG
b0463	STM0476	SOG
b0464	STM0477	SOG
b0465	STM0478	SOG
b0467	STM0481	SOG
b0469	STM0483	SOG
b0470	STM0484	SOG
b0472	STM0486	SOG
b0473	STM0487.S	SOG
b0474	STM0488	SOG
b0477	STM0491	SOG
b0478	STM0492	SOG
b0479	STM0493	SOG
b0480	STM0494	SOG
b0481	STM0495	SOG
b0482	STM0496	SOG
b0484	STM0498	SOG
b0488	STM0500	SOG
b0489	STM0501	SOG
b0490	STM0502	SOG
b0491	STM0503	SOG
b0492	STM0504	SOG
b0493	STM0505	SOG
b0494	STM0506	SOG
b0495	STM0507	SOG
b0496	STM0508	SOG
b0503	STM0513	SOG
b0522	STM0533	SOG
b0523	STM0534	SOG
b0524	STM0535	SOG
b0525	STM0536	SOG
b0526	STM0537	SOG
b0529	STM0542	SOG
b0576	STM0568	SOG
b0577	STM0569	SOG
b0578	STM0578	SOG
b0581	STM0583	SOG
b0584	STM0585	SOG
b0585	STM0586	SOG
b0586	STM0588	SOG
b0588	STM0590	SOG
b0589	STM0591	SOG
b0590	STM0592	SOG
b0591	STM0593	SOG

STM0502	KPN_00468	SOG
STM0503	KPN_00469	SOG
STM0504	KPN_00470	SOG
STM0505	KPN_00471	SOG
STM0506	KPN_00472	SOG
STM0507	KPN_00473	SOG
STM0508	KPN_00474	SOG
STM0513	KPN_00476	SOG
STM0533	KPN_00477	SOG
STM0534	KPN_00478	SOG
STM0535	KPN_00480	SOG
STM0536	KPN_00481	SOG
STM0537	KPN_00482	SOG
STM0542	KPN_00484	SOG
STM2557	KPN_00498	SOG
STM2558	KPN_00499	SOG
STM2559	KPN_00500	SOG
STM2560	KPN_00501	SOG
STM0568	KPN_00537	SOG
STM0569	KPN_00543	SOG
STM0578	KPN_00553	SOG
STM0585	KPN_00602	SOG
STM0590	KPN_00606	SOG
STM0591	KPN_00607	SOG
STM0592	KPN_00608	SOG
STM0593	KPN_00609	SOG
STM0594	KPN_00610	SOG
STM0596	KPN_00612	SOG
STM0597	KPN_00613	SOG
STM0598	KPN_00614	SOG
STM0600	KPN_00616	SOG
STM0605	KPN_00647	SOG
STM0608	KPN_00650	SOG
STM0609	KPN_00651	SOG
STM0627	KPN_00654	SOG
STM0633	KPN_00660	SOG
STM0634	KPN_00661	SOG
STM0635.S	KPN_00662	SOG
STM0637	KPN_00664	SOG
STM0638	KPN_00665	SOG
STM0639	KPN_00666	SOG
STM0640	KPN_00667	SOG
STM0641	KPN_00668	SOG
STM0645	KPN_00671	SOG
STM0646	KPN_00672	SOG
STM0647	KPN_00673	SOG

b0641	KPN_00673	SOG
b0642	KPN_00674	SOG
b0652	KPN_00678	SOG
b0653	KPN_00679	SOG
b0654	KPN_00680	SOG
b0655	KPN_00681	SOG
b0657	KPN_00682	SOG
b0658	KPN_00683	SOG
b0659	KPN_00684	SOG
b0660	KPN_00685	SOG
b0661	KPN_00686	SOG
b0662	KPN_00687	SOG
b0674	KPN_00695	SOG
b0675	KPN_00696	SOG
b0676	KPN_00697	SOG
b0677	KPN_00698	SOG
b0678	KPN_00699	SOG
b0679	KPN_00700	SOG
b0680	KPN_00701	SOG
b0681	KPN_00703	SOG
b0683	KPN_00706	SOG
b0684	KPN_00707	SOG
b0686	KPN_00709	SOG
b0687	KPN_00710	SOG
b0688	KPN_00711	SOG
b0694	KPN_00713	SOG
b0695	KPN_00714	SOG
b0697	KPN_00716	SOG
b0708	KPN_00719	SOG
b0709	KPN_00720	SOG
b0710	KPN_00721	SOG
b0711	KPN_00722	SOG
b0712	KPN_00723	SOG
b0713	KPN_00724	SOG
b0714	KPN_00726	SOG
b0720	KPN_00727	SOG
b0723	KPN_00730	SOG
b0724	KPN_00731	SOG
b0726	KPN_00732	SOG
b0727	KPN_00733	SOG
b0728	KPN_00734	SOG
b0729	KPN_00735	SOG
b0733	KPN_00736	SOG
b0734	KPN_00737	SOG
b0737	KPN_00741	SOG
b0738	KPN_00742	SOG

b0592	STM0594	SOG
b0593	STM0595	SOG
b0594	STM0596	SOG
b0595	STM0597	SOG
b0596	STM0598	SOG
b0598	STM0600	SOG
b0599	STM0602	SOG
b0600	STM0603	SOG
b0602	STM0605	SOG
b0605	STM0608	SOG
b0606	STM0609	SOG
b0607	STM0614	SOG
b0608	STM0615	SOG
b0621	STM0627	SOG
b0622	STM0628	SOG
b0628	STM0633	SOG
b0630	STM0635.S	SOG
b0632	STM0637	SOG
b0633	STM0638	SOG
b0634	STM0639	SOG
b0635	STM0640	SOG
b0636	STM0641	SOG
b0639	STM0645	SOG
b0640	STM0646	SOG
b0641	STM0647	SOG
b0642	STM0648	SOG
b0643	STM0653	SOG
b0651	STM0661	SOG
b0652	STM0662	SOG
b0653	STM0663	SOG
b0654	STM0664	SOG
b0655	STM0665	SOG
b0657	STM0666	SOG
b0658	STM0667	SOG
b0659	STM0668	SOG
b0660	STM0669	SOG
b0661	STM0670	SOG
b0662	STM0671	SOG
b0674	STM0680	SOG
b0675	STM0681	SOG
b0676	STM0682	SOG
b0677	STM0683	SOG
b0678	STM0684	SOG
b0679	STM0685	SOG
b0680	STM0686	SOG
b0681	STM0687	SOG

STM0648	KPN_00674	SOG
STM0662	KPN_00678	SOG
STM0663	KPN_00679	SOG
STM0664	KPN_00680	SOG
STM0665	KPN_00681	SOG
STM0666	KPN_00682	SOG
STM0667	KPN_00683	SOG
STM0668	KPN_00684	SOG
STM0669	KPN_00685	SOG
STM0670	KPN_00686	SOG
STM0680	KPN_00695	SOG
STM0681	KPN_00696	SOG
STM0682	KPN_00697	SOG
STM0683	KPN_00698	SOG
STM0684	KPN_00699	SOG
STM0685	KPN_00700	SOG
STM0686	KPN_00701	SOG
STM0687	KPN_00703	SOG
STM0693	KPN_00706	SOG
STM0694	KPN_00707	SOG
STM0696	KPN_00709	SOG
STM0697	KPN_00710	SOG
STM0698	KPN_00711	SOG
STM0702	KPN_00713	SOG
STM0703	KPN_00714	SOG
STM0705	KPN_00716	SOG
STM0709	KPN_00719	SOG
STM0710	KPN_00720	SOG
STM0711	KPN_00721	SOG
STM0712	KPN_00722	SOG
STM0713	KPN_00723	SOG
STM0714	KPN_00724	SOG
STM0728	KPN_00726	SOG
STM0730	KPN_00727	SOG
STM0734	KPN_00730	SOG
STM0735	KPN_00731	SOG
STM0736	KPN_00732	SOG
STM0737	KPN_00733	SOG
STM0738	KPN_00734	SOG
STM0739	KPN_00735	SOG
STM0740	KPN_00736	SOG
STM0741	KPN_00737	SOG
STM0745	KPN_00741	SOG
STM0746	KPN_00742	SOG
STM0748	KPN_00744	SOG
STM0749	KPN_00745	SOG

b0740	KPN_00744	SOG
b0741	KPN_00745	SOG
b0742	KPN_00746	SOG
b0750	KPN_00754	SOG
b0751	KPN_00755	SOG
b0754	KPN_00758	SOG
b0755	KPN_00768	SOG
b0756	KPN_00770	SOG
b0757	KPN_00771	SOG
b0758	KPN_00772	SOG
b0759	KPN_00773	SOG
b0763	KPN_00777	SOG
b0764	KPN_00778	SOG
b0765	KPN_00779	SOG
b0766	KPN_00780	SOG
b0767	KPN_00781	SOG
b0772	KPN_00791	SOG
b0773	KPN_00798	SOG
b0774	KPN_00799	SOG
b0775	KPN_00800	SOG
b0779	KPN_00804	SOG
b4113	KPN_00807	SOG
b4114	KPN_00808	SOG
b0781	KPN_00810	SOG
b0782	KPN_00811	SOG
b0783	KPN_00812	SOG
b0785	KPN_00814	SOG
b0786	KPN_00815	SOG
b0789	KPN_00817	SOG
b0790	KPN_00818	SOG
b0792	KPN_00820	SOG
b0793	KPN_00821	SOG
b0794	KPN_00822	SOG
b0795	KPN_00823	SOG
b0796	KPN_00824	SOG
b0797	KPN_00830	SOG
b0799	KPN_00831	SOG
b0807	KPN_00836	SOG
b0808	KPN_00837	SOG
b0809	KPN_00838	SOG
b0810	KPN_00839	SOG
b0811	KPN_00840	SOG
b0812	KPN_00841	SOG
b0814	KPN_00843	SOG
b0815	KPN_00844	SOG
b0817	KPN_00845	SOG

b0683	STM0693	SOG
b0684	STM0694	SOG
b0686	STM0696	SOG
b0687	STM0697	SOG
b0688	STM0698	SOG
b0694	STM0702	SOG
b0695	STM0703	SOG
b0696	STM0704	SOG
b0697	STM0705	SOG
b0708	STM0709	SOG
b0709	STM0710	SOG
b0710	STM0711	SOG
b0711	STM0712	SOG
b0712	STM0713	SOG
b0713	STM0714	SOG
b0714	STM0728	SOG
b0715	STM0729	SOG
b0720	STM0730	SOG
b0723	STM0734	SOG
b0724	STM0735	SOG
b0726	STM0736	SOG
b0727	STM0737	SOG
b0728	STM0738	SOG
b0729	STM0739	SOG
b0733	STM0740	SOG
b0734	STM0741	SOG
b0737	STM0745	SOG
b0738	STM0746	SOG
b0740	STM0748	SOG
b0741	STM0749	SOG
b0742	STM0750	SOG
b0750	STM0756	SOG
b0751	STM0757	SOG
b0752	STM0758	SOG
b0754	STM0760	SOG
b0755	STM0772	SOG
b0756	STM0773	SOG
b0757	STM0774	SOG
b0758	STM0775	SOG
b0759	STM0776	SOG
b0760	STM0778	SOG
b0761	STM0779	SOG
b0763	STM0781	SOG
b0764	STM0782	SOG
b0765	STM0783	SOG
b0766	STM0784	SOG

STM0750	KPN_00746	SOG
STM0756	KPN_00754	SOG
STM0757	KPN_00755	SOG
STM0760	KPN_00758	SOG
STM0772	KPN_00768	SOG
STM0773	KPN_00770	SOG
STM0774	KPN_00771	SOG
STM0775	KPN_00772	SOG
STM0776	KPN_00773	SOG
STM0779	KPN_00775	SOG
STM0781	KPN_00777	SOG
STM0782	KPN_00778	SOG
STM0783	KPN_00779	SOG
STM0784	KPN_00780	SOG
STM0785	KPN_00781	SOG
STM0786	KPN_00791	SOG
STM0792	KPN_00798	SOG
STM0793	KPN_00799	SOG
STM0794	KPN_00800	SOG
STM0795	KPN_00801	SOG
STM0797	KPN_00803	SOG
STM0798	KPN_00804	SOG
STM4292	KPN_00807	SOG
STM4293	KPN_00808	SOG
STM0801	KPN_00809	SOG
STM0802	KPN_00810	SOG
STM0803	KPN_00811	SOG
STM0804	KPN_00812	SOG
STM0806	KPN_00814	SOG
STM0807	KPN_00815	SOG
STM0812	KPN_00817	SOG
STM0813	KPN_00818	SOG
STM0815	KPN_00820	SOG
STM0816	KPN_00821	SOG
STM0817	KPN_00822	SOG
STM0818	KPN_00823	SOG
STM0819	KPN_00824	SOG
STM0820	KPN_00830	SOG
STM0821	KPN_00831	SOG
STM0822	KPN_00832	SOG
STM0826	KPN_00836	SOG
STM0827	KPN_00837	SOG
STM0828	KPN_00838	SOG
STM0829	KPN_00839	SOG
STM0830	KPN_00840	SOG
STM0831	KPN_00841	SOG

b0818	KPN_00846	SOG
b0819	KPN_00849	SOG
b0820	KPN_00851	SOG
b0821	KPN_00855	SOG
b0823	KPN_00857	SOG
b0824	KPN_00858	SOG
b0826	KPN_00859	SOG
b0827	KPN_00860	SOG
b0830	KPN_00863	SOG
b0831	KPN_00864	SOG
b0832	KPN_00865	SOG
b0835	KPN_00866	SOG
b0838	KPN_00870	SOG
b0839	KPN_00871	SOG
b0840	KPN_00872	SOG
b0841	KPN_00873	SOG
b0842	KPN_00874	SOG
b0847	KPN_00878	SOG
b0851	KPN_00882	SOG
b0852	KPN_00883	SOG
b0854	KPN_00885	SOG
b0855	KPN_00886	SOG
b0856	KPN_00887	SOG
b0857	KPN_00888	SOG
b0858	KPN_00889	SOG
b0860	KPN_00893	SOG
b0861	KPN_00894	SOG
b0862	KPN_00895	SOG
b0863	KPN_00896	SOG
b0864	KPN_00897	SOG
b0865	KPN_00898	SOG
b0867	KPN_00900	SOG
b0868	KPN_00901	SOG
b0869	KPN_00902	SOG
b0870	KPN_00903	SOG
b0871	KPN_00904	SOG
b0872	KPN_00906	SOG
b0873	KPN_00907	SOG
b0874	KPN_00908	SOG
b0876	KPN_00910	SOG
b0879	KPN_00912	SOG
b0882	KPN_00915	SOG
b0885	KPN_00917	SOG
b0886	KPN_00918	SOG
b0887	KPN_00919	SOG
b0888	KPN_00920	SOG

b0767	STM0785	SOG
b0772	STM0786	SOG
b0773	STM0792	SOG
b0774	STM0793	SOG
b0775	STM0794	SOG
b0776	STM0795	SOG
b0777	STM0796	SOG
b0778	STM0797	SOG
b0779	STM0798	SOG
b0780	STM0801	SOG
b0781	STM0802	SOG
b0782	STM0803	SOG
b0783	STM0804	SOG
b0785	STM0806	SOG
b0786	STM0807	SOG
b0788	STM0811	SOG
b0789	STM0812	SOG
b0790	STM0813	SOG
b0792	STM0815	SOG
b0793	STM0816	SOG
b0794	STM0817	SOG
b0795	STM0818	SOG
b0796	STM0819	SOG
b0797	STM0820	SOG
b0799	STM0821	SOG
b0800	STM0822	SOG
b0807	STM0826	SOG
b0808	STM0827	SOG
b0809	STM0828	SOG
b0810	STM0829	SOG
b0811	STM0830	SOG
b0812	STM0831	SOG
b0813	STM0832	SOG
b0814	STM0833	SOG
b0815	STM0834	SOG
b0818	STM0836	SOG
b0819	STM0837	SOG
b0820	STM0838	SOG
b0821	STM0841	SOG
b0822	STM0842	COG
b0823	STM0843	SOG
b0824	STM0844	SOG
b0826	STM0845	SOG
b0827	STM0846	SOG
b0828	STM0847	SOG
b0829	STM0848	SOG

STM0833	KPN_00843	SOG
STM0834	KPN_00844	SOG
STM0837	KPN_00849	SOG
STM0838	KPN_00851	SOG
STM0841	KPN_00855	SOG
STM0843	KPN_00857	SOG
STM0844	KPN_00858	SOG
STM0845	KPN_00859	SOG
STM0846	KPN_00860	SOG
STM0848	KPN_00862	SOG
STM0849	KPN_00863	SOG
STM0850	KPN_00864	SOG
STM0851	KPN_00865	SOG
STM0852	KPN_00866	SOG
STM0863	KPN_00871	SOG
STM0864	KPN_00872	SOG
STM0865	KPN_00873	SOG
STM0866	KPN_00874	SOG
STM0870	KPN_00878	SOG
STM0874	KPN_00882	SOG
STM0875	KPN_00883	SOG
STM0876	KPN_00884	SOG
STM0877	KPN_00885	SOG
STM0878	KPN_00886	SOG
STM0879	KPN_00887	SOG
STM0880	KPN_00888	SOG
STM0881	KPN_00889	SOG
STM0882	KPN_00890	SOG
STM0887	KPN_00893	SOG
STM0888	KPN_00894	SOG
STM0889	KPN_00895	SOG
STM0890	KPN_00896	SOG
STM0891	KPN_00897	SOG
STM0892	KPN_00898	SOG
STM0931	KPN_00900	SOG
STM0932	KPN_00901	SOG
STM0933	KPN_00902	SOG
STM0934	KPN_00903	SOG
STM0935	KPN_00904	SOG
STM0936	KPN_00906	SOG
STM0937	KPN_00907	SOG
STM0938	KPN_00908	SOG
STM0939	KPN_00910	SOG
STM0942	KPN_00912	SOG
STM0945	KPN_00915	SOG
STM0955	KPN_00917	SOG

b0890	KPN_00922	SOG
b0891	KPN_00923	SOG
b0892	KPN_00924	SOG
b0893	KPN_00925	SOG
b0894	KPN_00926	SOG
b0898	KPN_00929	SOG
b0902	KPN_00930	SOG
b0903	KPN_00931	SOG
b0904	KPN_00932	SOG
b0905	KPN_00933	SOG
b0906	KPN_00934	SOG
b0907	KPN_00935	SOG
b0908	KPN_00936	SOG
b0910	KPN_00937	SOG
b0911	KPN_00938	SOG
b0914	KPN_00941	SOG
b0918	KPN_00945	SOG
b0919	KPN_00946	SOG
b0920	KPN_00947	SOG
b0921	KPN_00948	SOG
b0922	KPN_00949	SOG
b0923	KPN_00950	SOG
b0924	KPN_00951	SOG
b0925	KPN_00952	SOG
b0926	KPN_00953	SOG
b0927	KPN_00954	SOG
b0928	KPN_00955	SOG
b0930	KPN_00961	SOG
b0931	KPN_00967	SOG
b0932	KPN_00968	SOG
b0945	KPN_00974	SOG
b0946	KPN_00975	SOG
b0948	KPN_00977	SOG
b0949	KPN_00978	SOG
b0950	KPN_00979	SOG
b0951	KPN_00980	SOG
b0954	KPN_00983	SOG
b0955	KPN_00984	SOG
b0956	KPN_00985	SOG
b0957	KPN_00986	SOG
b0958	KPN_00987	SOG
b0960	KPN_00989	SOG
b0962	KPN_00991	SOG
b0963	KPN_00992	SOG
b0967	KPN_01001	SOG
b0970	KPN_01006	SOG

b0830	STM0849	SOG
b0831	STM0850	SOG
b0832	STM0851	SOG
b0835	STM0852	SOG
b0838	STM0862	SOG
b0839	STM0863	SOG
b0840	STM0864	SOG
b0841	STM0865	SOG
b0842	STM0866	SOG
b0845	STM0868	SOG
b0847	STM0870	SOG
b0851	STM0874	SOG
b0852	STM0875	SOG
b0853	STM0876	SOG
b0854	STM0877	SOG
b0855	STM0878	SOG
b0856	STM0879	SOG
b0857	STM0880	SOG
b0858	STM0881	SOG
b0859	STM0882	SOG
b0860	STM0887	SOG
b0861	STM0888	SOG
b0862	STM0889	SOG
b0863	STM0890	SOG
b0864	STM0891	SOG
b0867	STM0931	SOG
b0868	STM0932	SOG
b0869	STM0933	SOG
b0870	STM0934	SOG
b0871	STM0935	SOG
b0872	STM0936	SOG
b0873	STM0937	SOG
b0874	STM0938	SOG
b0876	STM0939	SOG
b0879	STM0942	SOG
b0882	STM0945	SOG
b0885	STM0955	SOG
b0886	STM0956	SOG
b0887	STM0957	SOG
b0888	STM0958	SOG
b0890	STM0960	SOG
b0891	STM0961	SOG
b0892	STM0962	SOG
b0893	STM0963	SOG
b0894	STM0964	SOG
b0896	STM0966	SOG

STM0956	KPN_00918	SOG
STM0957	KPN_00919	SOG
STM0958	KPN_00920	SOG
STM0960	KPN_00922	SOG
STM0961	KPN_00923	SOG
STM0962	KPN_00924	SOG
STM0963	KPN_00925	SOG
STM0964	KPN_00926	SOG
STM0966	KPN_00928	SOG
STM0968	KPN_00929	SOG
STM0970	KPN_00930	SOG
STM0973	KPN_00931	SOG
STM0974	KPN_00932	SOG
STM0975	KPN_00933	SOG
STM0976	KPN_00934	SOG
STM0977	KPN_00935	SOG
STM0978	KPN_00936	SOG
STM0980	KPN_00937	SOG
STM0981	KPN_00938	SOG
STM0984	KPN_00941	SOG
STM0986	KPN_00943	SOG
STM0988	KPN_00945	SOG
STM0989	KPN_00946	SOG
STM0990	KPN_00947	SOG
STM0991	KPN_00948	SOG
STM0992	KPN_00949	SOG
STM0993	KPN_00950	SOG
STM0994	KPN_00951	SOG
STM0995	KPN_00952	SOG
STM0996	KPN_00953	SOG
STM0997	KPN_00954	SOG
STM0998	KPN_00955	SOG
STM1000	KPN_00961	SOG
STM1004	KPN_00967	SOG
STM1057	KPN_00968	SOG
STM1058	KPN_00974	SOG
STM1059	KPN_00975	SOG
STM1060	KPN_00976	SOG
STM1061	KPN_00977	SOG
STM1062	KPN_00978	SOG
STM1063	KPN_00979	SOG
STM1064	KPN_00980	SOG
STM1065	KPN_00981	SOG
STM1067	KPN_00983	SOG
STM1068	KPN_00984	SOG
STM1069	KPN_00985	SOG

b1002	KPN_01027	SOG
b1004	KPN_01029	SOG
b1013	KPN_01039	SOG
b1015	KPN_01042	SOG
b1035	KPN_01058	SOG
b1048	KPN_01063	SOG
b1049	KPN_01064	SOG
b1054	KPN_01068	SOG
b1055	KPN_01069	SOG
b1059	KPN_01071	SOG
b1062	KPN_01074	SOG
b1063	KPN_01075	SOG
b1065	KPN_01076	SOG
b1067	KPN_01078	SOG
b1069	KPN_01080	SOG
b1084	KPN_01083	SOG
b1086	KPN_01084	SOG
b1088	KPN_01086	SOG
b1090	KPN_01088	SOG
b1091	KPN_01089	SOG
b1092	KPN_01090	SOG
b1093	KPN_01091	SOG
b1095	KPN_01093	SOG
b1096	KPN_01094	SOG
b1097	KPN_01095	SOG
b1099	KPN_01097	SOG
b1100	KPN_01098	SOG
b1101	KPN_01099	SOG
b1105	KPN_01102	SOG
b1107	KPN_01104	SOG
b1108	KPN_01105	SOG
b1109	KPN_01106	SOG
b1110	KPN_01107	SOG
b1116	KPN_01113	SOG
b1117	KPN_01114	SOG
b1118	KPN_01115	SOG
b1119	KPN_01116	SOG
b1123	KPN_01129	SOG
b1124	KPN_01130	SOG
b1125	KPN_01131	SOG
b1126	KPN_01132	SOG
b1127	KPN_01133	SOG
b1128	KPN_01134	SOG
b1130	KPN_01136	SOG
b1131	KPN_01139	SOG
b1132	KPN_01140	SOG

b0898	STM0968	SOG
b0902	STM0970	SOG
b0903	STM0973	SOG
b0904	STM0974	SOG
b0905	STM0975	SOG
b0906	STM0976	SOG
b0907	STM0977	SOG
b0908	STM0978	SOG
b0910	STM0980	SOG
b0911	STM0981	SOG
b0914	STM0984	SOG
b0915	STM0985	SOG
b0916	STM0986	SOG
b0918	STM0988	SOG
b0919	STM0989	SOG
b0920	STM0990	SOG
b0921	STM0991	SOG
b0922	STM0992	SOG
b0923	STM0993	SOG
b0924	STM0994	SOG
b0925	STM0995	SOG
b0926	STM0996	SOG
b0927	STM0997	SOG
b0928	STM0998	SOG
b0930	STM1000	SOG
b0931	STM1004	SOG
b0932	STM1057	SOG
b0945	STM1058	SOG
b0946	STM1059	SOG
b0947	STM1060	SOG
b0948	STM1061	SOG
b0949	STM1062	SOG
b0950	STM1063	SOG
b0951	STM1064	SOG
b0952	STM1065	SOG
b0954	STM1067	SOG
b0955	STM1068	SOG
b0956	STM1069	SOG
b0957	STM1070	SOG
b0958	STM1071	SOG
b0959	STM1072	SOG
b0960	STM1073	SOG
b0961	STM1074	SOG
b0962	STM1075	SOG
b0963	STM1076	SOG
b0967	STM1080	SOG

STM1070	KPN_00986	SOG
STM1071	KPN_00987	SOG
STM1073	KPN_00989	SOG
STM1074	KPN_00990	SOG
STM1076	KPN_00992	SOG
STM1080	KPN_01001	SOG
STM1085	KPN_01006	SOG
STM1119	KPN_01029	SOG
STM1122	KPN_01039	SOG
STM1125	KPN_01042	SOG
STM1150	KPN_01063	SOG
STM1151	KPN_01064	SOG
STM1154	KPN_01067	SOG
STM1155	KPN_01068	SOG
STM1156	KPN_01069	SOG
STM1163	KPN_01074	SOG
STM1164	KPN_01075	SOG
STM1166	KPN_01076	SOG
STM1167	KPN_01077	SOG
STM1168	KPN_01078	SOG
STM1169	KPN_01079	SOG
STM1170	KPN_01080	SOG
STM1185	KPN_01083	SOG
STM1187	KPN_01084	SOG
STM1189	KPN_01085	SOG
STM1190	KPN_01086	SOG
STM1192	KPN_01088	SOG
STM1193	KPN_01089	SOG
STM1194	KPN_01090	SOG
STM1195	KPN_01091	SOG
STM1197	KPN_01093	SOG
STM1198	KPN_01094	SOG
STM1199	KPN_01095	SOG
STM1200	KPN_01096	SOG
STM1202	KPN_01098	SOG
STM1203	KPN_01099	SOG
STM1207	KPN_01102	SOG
STM1208	KPN_01103	SOG
STM1209	KPN_01104	SOG
STM1210	KPN_01105	SOG
STM1211	KPN_01106	SOG
STM1212	KPN_01107	SOG
STM1213	KPN_01109	SOG
STM1217.S	KPN_01113	SOG
STM1218	KPN_01114	SOG
STM1219	KPN_01115	SOG

b1133	KPN_01141	SOG
b1134	KPN_01142	SOG
b1135	KPN_01143	SOG
b1136	KPN_01144	SOG
b1257	KPN_01151	COG
b1789	KPN_01178	COG
b1184	KPN_01184	SOG
b1784	KPN_01192	COG
b1783	KPN_01193	COG
b1782	KPN_01194	COG
b1780	KPN_01196	COG
b1779	KPN_01197	COG
b1767	KPN_01203	COG
b1766	KPN_01204	COG
b1765	KPN_01205	COG
b1764	KPN_01207	COG
b1763	KPN_01208	COG
b1761	KPN_01210	COG
b1749	KPN_01220	COG
b1748	KPN_01221	COG
b1746	KPN_01223	COG
b1743	KPN_01226	COG
b1740	KPN_01228	COG
b1737	KPN_01231	COG
b1735	KPN_01233	COG
b1734	KPN_01234	COG
b1733	KPN_01235	COG
b1732	KPN_01236	COG
b1729	KPN_01238	COG
b1728	KPN_01239	COG
b1727	KPN_01241	COG
b1256	KPN_01248	SOG
b1260	KPN_01253	SOG
b1261	KPN_01254	SOG
b1262	KPN_01255	SOG
b1263	KPN_01256	SOG
b1264	KPN_01257	SOG
b1266	KPN_01258	SOG
b1267	KPN_01259	SOG
b1269	KPN_01265	SOG
b1270	KPN_01266	SOG
b1271	KPN_01267	SOG
b1272	KPN_01268	SOG
b1274	KPN_01270	SOG
b1275	KPN_01271	SOG
b1276	KPN_01272	SOG

b0970	STM1085	SOG
b1000	STM1112	SOG
b1002	STM1117	SOG
b1004	STM1119	SOG
b1013	STM1122	SOG
b1015	STM1125	SOG
b1034	STM1136	SOG
b1035	STM1137	SOG
b1036	STM1138	SOG
b1045	STM1147	SOG
b1047	STM1149	SOG
b1048	STM1150	SOG
b1049	STM1151	SOG
b1053	STM1154	SOG
b1054	STM1155	SOG
b1055	STM1156	SOG
b1059	STM1160	SOG
b1062	STM1163	SOG
b1063	STM1164	SOG
b1065	STM1166	SOG
b1066	STM1167	SOG
b1067	STM1168	SOG
b1068	STM1169	SOG
b1069	STM1170	SOG
b1084	STM1185	SOG
b1086	STM1187	SOG
b1087	STM1189	SOG
b1088	STM1190	SOG
b1090	STM1192	SOG
b1091	STM1193	SOG
b1092	STM1194	SOG
b1093	STM1195	SOG
b1095	STM1197	SOG
b1096	STM1198	SOG
b1097	STM1199	SOG
b1098	STM1200	SOG
b1099	STM1201	SOG
b1100	STM1202	SOG
b1101	STM1203	SOG
b1105	STM1207	SOG
b1106	STM1208	SOG
b1107	STM1209	SOG
b1108	STM1210	SOG
b1109	STM1211	SOG
b1110	STM1212	SOG
b1111	STM1213	SOG

STM1220	KPN_01116	SOG
STM1222	KPN_01129	SOG
STM1223	KPN_01130	SOG
STM1225	KPN_01131	SOG
STM1226	KPN_01132	SOG
STM1227	KPN_01133	SOG
STM1229	KPN_01134	SOG
STM1230	KPN_01135	SOG
STM1231	KPN_01136	SOG
STM1232	KPN_01139	SOG
STM1233	KPN_01140	SOG
STM1234.S	KPN_01141	SOG
STM1235	KPN_01142	SOG
STM1237	KPN_01143	SOG
STM1238	KPN_01144	SOG
STM0615	KPN_01148	COG
STM1284	KPN_01192	SOG
STM1285	KPN_01193	SOG
STM1286	KPN_01194	SOG
STM1289	KPN_01196	SOG
STM1290	KPN_01197	SOG
STM1293	KPN_01202	SOG
STM1294	KPN_01203	SOG
STM1295	KPN_01204	SOG
STM1297	KPN_01207	SOG
STM1298	KPN_01208	SOG
STM1299	KPN_01210	SOG
STM1302	KPN_01220	SOG
STM1303	KPN_01221	SOG
STM1308	KPN_01226	SOG
STM1310	KPN_01228	SOG
STM1313	KPN_01231	SOG
STM1315	KPN_01233	SOG
STM1316	KPN_01234	SOG
STM1317	KPN_01235	SOG
STM1318	KPN_01236	SOG
STM1320	KPN_01238	SOG
STM1321	KPN_01239	SOG
STM1323	KPN_01242	SOG
STM1735	KPN_01245	COG
STM1734	KPN_01246	COG
STM1726	KPN_01254	COG
STM1724	KPN_01256	COG
STM1723	KPN_01257	COG
STM1721	KPN_01258	COG
STM1720	KPN_01259	COG

b1277	KPN_01273	SOG
b1278	KPN_01274	SOG
b1280	KPN_01276	SOG
b1286	KPN_01282	SOG
b1287	KPN_01283	SOG
b1288	KPN_01284	SOG
b1290	KPN_01287	SOG
b1291	KPN_01288	SOG
b1292	KPN_01289	SOG
b1293	KPN_01290	SOG
b1294	KPN_01291	SOG
b1303	KPN_01296	SOG
b1304	KPN_01297	SOG
b1321	KPN_01301	SOG
b1322	KPN_01302	SOG
b1323	KPN_01305	SOG
b1329	KPN_01310	SOG
b1342	KPN_01373	SOG
b1343	KPN_01374	SOG
b1344	KPN_01375	SOG
b1378	KPN_01446	SOG
b1380	KPN_01449	SOG
b1412	KPN_01483	SOG
b1413	KPN_01484	SOG
b1929	KPN_01506	COG
b1612	KPN_01516	COG
b1608	KPN_01521	COG
b1605	KPN_01524	COG
b1604	KPN_01525	COG
b1603	KPN_01526	COG
b1602	KPN_01527	COG
b1333	KPN_01529	SOG
b1334	KPN_01530	SOG
b1335	KPN_01531	SOG
b1340	KPN_01536	SOG
b1598	KPN_01567	COG
b1594	KPN_01574	COG
b1593	KPN_01575	COG
b1587	KPN_01580	COG
b1584	KPN_01583	COG
b1540	KPN_01589	COG
b1539	KPN_01590	COG
b2738	KPN_01603	COG
b1530	KPN_01625	COG
b1529	KPN_01627	COG
b3942	KPN_01819	SOG

b1113	STM1215	SOG
b1116	STM1217.S	SOG
b1117	STM1218	SOG
b1118	STM1219	SOG
b1119	STM1220	SOG
b1123	STM1222	SOG
b1124	STM1223	SOG
b1125	STM1225	SOG
b1126	STM1226	SOG
b1127	STM1227	SOG
b1128	STM1229	SOG
b1129	STM1230	SOG
b1130	STM1231	SOG
b1131	STM1232	SOG
b1132	STM1233	SOG
b1133	STM1234.S	SOG
b1134	STM1235	SOG
b1135	STM1237	SOG
b1136	STM1238	SOG
b1175	STM1815	COG
b1176	STM1814	COG
b1180	STM1812	COG
b1181	STM1811	COG
b1185	STM1807	COG
b1186	STM1806	COG
b1187	STM1805	COG
b1188	STM1804.S	COG
b1189	STM1803	COG
b1190	STM1802	COG
b1191	STM1801	COG
b1192	STM1800	COG
b1193	STM1799	COG
b1197	STM1796	COG
b1203	STM1784	COG
b1204	STM1783.S	COG
b1206	STM1781	COG
b1207	STM1780	COG
b1208	STM1779	COG
b1209	STM1778	COG
b1210	STM1777	COG
b1214	STM1773	COG
b1215	STM1772	COG
b1216	STM1771	COG
b1220	STM1768	COG
b1221	STM1767	COG
b1222	STM1766	COG

STM1719	KPN_01265	COG
STM1718	KPN_01266	COG
STM1717	KPN_01267	COG
STM1716	KPN_01268	COG
STM1714	KPN_01270	COG
STM1713	KPN_01271	COG
STM1712	KPN_01272	COG
STM1711	KPN_01273	COG
STM1708	KPN_01276	COG
STM1704	KPN_01280	COG
STM1702	KPN_01282	COG
STM1700	KPN_01284	COG
STM1695	KPN_01288	COG
STM1694	KPN_01289	COG
STM1693	KPN_01290	COG
STM1692	KPN_01291	COG
STM1691	KPN_01296	COG
STM1690	KPN_01297	COG
STM1685	KPN_01301	COG
STM1683	KPN_01305	COG
STM1680	KPN_01309	COG
STM1679	KPN_01310	COG
STM1656	KPN_01373	COG
STM1654	KPN_01375	COG
STM1053	KPN_01443	SOG
STM1652	KPN_01444	COG
STM1651	KPN_01446	COG
STM1647	KPN_01449	COG
STM1642	KPN_01483	COG
STM1641	KPN_01484	COG
STM1965	KPN_01506	COG
STM1468	KPN_01516	SOG
STM1469	KPN_01517	SOG
STM1470	KPN_01518	SOG
STM1477	KPN_01524	SOG
STM1478	KPN_01525	SOG
STM1479	KPN_01526	SOG
STM1480	KPN_01527	SOG
STM1660.S	KPN_01530	COG
STM1659	KPN_01531	COG
STM1658	KPN_01536	COG
STM1484	KPN_01567	SOG
STM1486	KPN_01572	SOG
STM1488	KPN_01574	SOG
STM1489	KPN_01575	SOG
STM1496	KPN_01578	SOG

b1479	KPN_01847	COG
b1478	KPN_01853	COG
b1476	KPN_01865	COG
b1475	KPN_01866	COG
b1474	KPN_01867	COG
b1468	KPN_01880	COG
b1467	KPN_01881	COG
b1466	KPN_01882	COG
b1463	KPN_01884	COG
b1453	KPN_01906	COG
b1452	KPN_01908	COG
b0651	KPN_01912	COG
b1449	KPN_01913	COG
b1448	KPN_01915	COG
b1439	KPN_01929	COG
b1435	KPN_01934	COG
b1434	KPN_01935	COG
b1424	KPN_01944	COG
b1423	KPN_01947	COG
b1422	KPN_01950	COG
b0356	KPN_01952	SOG
b1624	KPN_01959	SOG
b1627	KPN_01964	SOG
b1628	KPN_01965	SOG
b1629	KPN_01966	SOG
b1630	KPN_01967	SOG
b1631	KPN_01968	SOG
b1632	KPN_01969	SOG
b1633	KPN_01970	SOG
b1634	KPN_01972	SOG
b1636	KPN_01974	SOG
b1637	KPN_01975	SOG
b1638	KPN_01976	SOG
b1640	KPN_01978	SOG
b1641	KPN_01979	SOG
b1642	KPN_01981	SOG
b1644	KPN_01983	SOG
b1647	KPN_01986	SOG
b1649	KPN_01988	SOG
b1650	KPN_01989	SOG
b1652	KPN_01991	SOG
b1655	KPN_01993	SOG
b1656	KPN_01994	SOG
b1658	KPN_01996	SOG
b1659	KPN_01997	SOG
b1660	KPN_01998	SOG

b1223	STM1765	COG
b1224	STM1764	COG
b1225	STM1763	COG
b1226	STM1762	COG
b1227	STM1761	COG
b1232	STM1756	COG
b1233	STM1755	COG
b1234	STM1754	COG
b1235	STM1753	COG
b1236	STM1752	COG
b1238	STM1750	COG
b1241	STM1749	COG
b1242	STM1748	COG
b1243	STM1746.S	COG
b1244	STM1745	COG
b1245	STM1744	COG
b1246	STM1743	COG
b1247	STM1742	COG
b1249	STM1739	COG
b1252	STM1737	COG
b1254	STM1735	COG
b1255	STM1734	COG
b1256	STM1732	COG
b1260	STM1727	COG
b1261	STM1726	COG
b1262	STM1725	COG
b1263	STM1724	COG
b1264	STM1723	COG
b1266	STM1721	COG
b1267	STM1720	COG
b1269	STM1719	COG
b1270	STM1718	COG
b1271	STM1717	COG
b1272	STM1716	COG
b1274	STM1714	COG
b1275	STM1713	COG
b1276	STM1712	COG
b1277	STM1711	COG
b1278	STM1710	COG
b1280	STM1708	COG
b1281	STM1707	COG
b1284	STM1704	COG
b1286	STM1702	COG
b1288	STM1700	COG
b1290	STM1696	COG
b1291	STM1695	COG

STM1499	KPN_01580	SOG
STM1502	KPN_01583	SOG
STM1508	KPN_01587	SOG
STM1510	KPN_01589	SOG
STM1511	KPN_01590	SOG
STM2916	KPN_01603	SOG
STM1520	KPN_01625	SOG
STM4106	KPN_01819	SOG
STM1566	KPN_01847	SOG
STM1568	KPN_01865	SOG
STM1569	KPN_01866	SOG
STM1570	KPN_01867	SOG
STM1577	KPN_01880	SOG
STM1578	KPN_01881	SOG
STM1579	KPN_01882	SOG
STM1580	KPN_01883	SOG
STM1584	KPN_01906	SOG
STM1586	KPN_01908	SOG
STM0661	KPN_01912	COG
STM1589	KPN_01913	SOG
STM1590	KPN_01915	SOG
STM1591	KPN_01916	SOG
STM1598	KPN_01929	SOG
STM1604	KPN_01934	SOG
STM1607	KPN_01939	SOG
STM1622	KPN_01944	SOG
STM1624	KPN_01947	SOG
STM1625	KPN_01950	SOG
STM1459	KPN_01964	COG
STM1457	KPN_01966	COG
STM1456	KPN_01967	COG
STM1454	KPN_01969	COG
STM1453	KPN_01970	COG
STM1452	KPN_01972	COG
STM1451	KPN_01973	COG
STM1450	KPN_01974	COG
STM1449	KPN_01975	COG
STM1448	KPN_01976	COG
STM1446	KPN_01978	COG
STM1445	KPN_01979	COG
STM1444	KPN_01981	COG
STM1442	KPN_01983	COG
STM1441	KPN_01984	COG
STM1439	KPN_01986	COG
STM1437	KPN_01988	COG
STM1436	KPN_01989	COG

b1661	KPN_01999	SOG
b1663	KPN_02001	SOG
b2194	KPN_02074	SOG
b2195	KPN_02075	SOG
b1676	KPN_02133	SOG
b1681	KPN_02138	SOG
b1682	KPN_02139	SOG
b1683	KPN_02140	SOG
b1687	KPN_02158	SOG
b1702	KPN_02160	SOG
b1703	KPN_02161	SOG
b1704	KPN_02162	SOG
b1706	KPN_02164	SOG
b1713	KPN_02175	SOG
b1714	KPN_02176	SOG
b1719	KPN_02180	SOG
b1722	KPN_02182	SOG
b1252	KPN_02186	COG
b1249	KPN_02190	COG
b1247	KPN_02193	COG
b1246	KPN_02194	COG
b1245	KPN_02195	COG
b1244	KPN_02196	COG
b1243	KPN_02197	COG
b1241	KPN_02199	COG
b1236	KPN_02202	COG
b1235	KPN_02203	COG
b1232	KPN_02206	COG
b1227	KPN_02211	COG
b1226	KPN_02212	COG
b1225	KPN_02213	COG
b1224	KPN_02214	COG
b1223	KPN_02215	COG
b1221	KPN_02217	COG
b1216	KPN_02229	COG
b1215	KPN_02230	COG
b1214	KPN_02231	COG
b1210	KPN_02235	COG
b1209	KPN_02236	COG
b1208	KPN_02237	COG
b1207	KPN_02238	COG
b1206	KPN_02239	COG
b1204	KPN_02242	COG
b1203	KPN_02243	COG
b0354	KPN_02277	SOG
b3114	KPN_02292	SOG

b1292	STM1694	COG
b1293	STM1693	COG
b1294	STM1692	COG
b1303	STM1691	COG
b1304	STM1690	COG
b1321	STM1685	COG
b1322	STM1684	COG
b1323	STM1683	COG
b1325	STM1681	COG
b1326	STM1680	COG
b1329	STM1679	COG
b1333	STM1661	COG
b1334	STM1660.S	COG
b1335	STM1659	COG
b1340	STM1658	COG
b1342	STM1656	COG
b1343	STM1655	COG
b1344	STM1654	COG
b1360	STM2625	COG
b1378	STM1651	COG
b1380	STM1647	COG
b1381	STM1646	COG
b1412	STM1642	COG
b1413	STM1641	COG
b1414	STM1640	COG
b1423	STM1624	COG
b1424	STM1622	COG
b1428	STM1610	COG
b1429	STM1609	COG
b1430	STM1608	COG
b1431	STM1607	COG
b1435	STM1604	COG
b1439	STM1598	COG
b1448	STM1590	COG
b1449	STM1589	COG
b1451	STM1587	COG
b1452	STM1586	COG
b1453	STM1584	COG
b1463	STM1582	COG
b1465	STM1580	COG
b1466	STM1579	COG
b1467	STM1578	COG
b1468	STM1577	COG
b1473	STM1571	COG
b1475	STM1569	COG
b1476	STM1568	COG

STM1434	KPN_01991	COG
STM1431	KPN_01994	COG
STM1430	KPN_01996	COG
STM1429	KPN_01997	COG
STM1427	KPN_01999	COG
STM1426	KPN_02000	COG
STM1425	KPN_02001	COG
STM3814	KPN_02076	SOG
STM1378	KPN_02133	COG
STM1372	KPN_02138	COG
STM1371	KPN_02139	COG
STM1370	KPN_02140	COG
STM1365	KPN_02158	COG
STM1364	KPN_02159	COG
STM1349	KPN_02160	COG
STM1348	KPN_02161	COG
STM1347	KPN_02162	COG
STM1345	KPN_02164	COG
STM1343	KPN_02165	COG
STM1341	KPN_02167	COG
STM1340	KPN_02169	COG
STM1338	KPN_02175	COG
STM1337	KPN_02176	COG
STM1333	KPN_02180	COG
STM1324	KPN_02185	COG
STM1737	KPN_02186	SOG
STM1739	KPN_02190	SOG
STM1742	KPN_02193	SOG
STM1743	KPN_02194	SOG
STM1744	KPN_02195	SOG
STM1745	KPN_02196	SOG
STM1746.S	KPN_02197	SOG
STM1748	KPN_02198	SOG
STM1749	KPN_02199	SOG
STM1750	KPN_02200	SOG
STM1752	KPN_02202	SOG
STM1753	KPN_02203	SOG
STM1756	KPN_02206	SOG
STM1761	KPN_02211	SOG
STM1762	KPN_02212	SOG
STM1763	KPN_02213	SOG
STM1764	KPN_02214	SOG
STM1765	KPN_02215	SOG
STM1767	KPN_02217	SOG
STM1771	KPN_02229	SOG
STM1772	KPN_02230	SOG

b3116	KPN_02294	SOG
b1193	KPN_02305	COG
b1191	KPN_02307	COG
b1189	KPN_02309	COG
b1188	KPN_02310	COG
b1187	KPN_02311	COG
b1186	KPN_02312	COG
b1185	KPN_02313	COG
b1181	KPN_02315	COG
b1180	KPN_02316	COG
b1176	KPN_02318	COG
b1175	KPN_02319	COG
b1804	KPN_02321	SOG
b1805	KPN_02322	SOG
b1806	KPN_02323	SOG
b1807	KPN_02324	SOG
b1808	KPN_02325	SOG
b1812	KPN_02328	SOG
b1814	KPN_02330	SOG
b1816	KPN_02332	SOG
b1817	KPN_02333	SOG
b1818	KPN_02334	SOG
b1819	KPN_02335	SOG
b1820	KPN_02336	SOG
b1821	KPN_02337	SOG
b1827	KPN_02343	SOG
b1829	KPN_02345	SOG
b1830	KPN_02346	SOG
b1831	KPN_02347	SOG
b1832	KPN_02348	SOG
b1833	KPN_02349	SOG
b1834	KPN_02350	SOG
b1844	KPN_02360	SOG
b1845	KPN_02361	SOG
b1850	KPN_02365	SOG
b1851	KPN_02366	SOG
b1852	KPN_02367	SOG
b1853	KPN_02368	SOG
b1854	KPN_02369	SOG
b1855	KPN_02370	SOG
b1856	KPN_02371	SOG
b1857	KPN_02372	SOG
b1858	KPN_02373	SOG
b1860	KPN_02375	SOG
b1861	KPN_02376	SOG
b1863	KPN_02377	SOG

b1479	STM1566	COG
b1511	STM4072	SOG
b1513	STM4074	SOG
b1514	STM4075	SOG
b1516	STM4077	SOG
b1517	STM4078	SOG
b1520	STM1527	COG
b1524	STM1525	COG
b1526	STM1523	COG
b1528	STM1522	COG
b1529	STM1521	COG
b1530	STM1520	COG
b1534	STM1516	COG
b1538	STM1512	COG
b1539	STM1511	COG
b1540	STM1510	COG
b1542	STM1508	COG
b1584	STM1502	COG
b1587	STM1499	COG
b1590	STM1496	COG
b1591	STM1495	COG
b1592	STM1490	COG
b1593	STM1489	COG
b1594	STM1488	COG
b1595	STM1487	COG
b1596	STM1486	COG
b1598	STM1484	COG
b1601	STM1481	COG
b1602	STM1480	COG
b1603	STM1479	COG
b1604	STM1478	COG
b1605	STM1477	COG
b1608	STM1475	COG
b1609	STM1471	COG
b1610	STM1470	COG
b1611	STM1469	COG
b1612	STM1468	COG
b1613	STM1467	COG
b1614	STM1466	COG
b1623	STM1463	COG
b1624	STM1462.S	COG
b1626	STM1460	COG
b1627	STM1459	COG
b1628	STM1458	COG
b1629	STM1457	COG
b1630	STM1456	COG

STM1773	KPN_02231	SOG
STM1775	KPN_02233	SOG
STM1777	KPN_02235	SOG
STM1778	KPN_02236	SOG
STM1779	KPN_02237	SOG
STM1780	KPN_02238	SOG
STM1781	KPN_02239	SOG
STM1783.S	KPN_02242	SOG
STM1784	KPN_02243	SOG
STM3241	KPN_02292	SOG
STM3243	KPN_02294	SOG
STM3244	KPN_02295	SOG
STM3245	KPN_02296	SOG
STM1799	KPN_02305	SOG
STM1801	KPN_02307	SOG
STM1802	KPN_02308	SOG
STM1803	KPN_02309	SOG
STM1804.S	KPN_02310	SOG
STM1805	KPN_02311	SOG
STM1806	KPN_02312	SOG
STM1807	KPN_02313	SOG
STM1811	KPN_02315	SOG
STM1812	KPN_02316	SOG
STM1814	KPN_02318	SOG
STM1815	KPN_02319	SOG
STM1817	KPN_02321	SOG
STM1818	KPN_02322	SOG
STM1819	KPN_02323	SOG
STM1820	KPN_02324	SOG
STM1821	KPN_02325	SOG
STM1824	KPN_02328	SOG
STM1825	KPN_02329	SOG
STM1826	KPN_02330	SOG
STM1828	KPN_02332	SOG
STM1830	KPN_02333	SOG
STM1831	KPN_02334	SOG
STM1832	KPN_02335	SOG
STM1833	KPN_02336	SOG
STM1834	KPN_02337	SOG
STM1842	KPN_02343	SOG
STM1844	KPN_02345	SOG
STM1845	KPN_02346	SOG
STM1846	KPN_02347	SOG
STM1847	KPN_02348	SOG
STM1849	KPN_02350	SOG
STM1878	KPN_02360	SOG

b1864	KPN_02378	SOG
b1865	KPN_02379	SOG
b1866	KPN_02380	SOG
b1868	KPN_02382	SOG
b1870	KPN_02384	SOG
b1871	KPN_02385	SOG
b1874	KPN_02386	SOG
b1875	KPN_02387	SOG
b1876	KPN_02388	SOG
b1895	KPN_02391	SOG
b1897	KPN_02393	SOG
b1905	KPN_02402	SOG
b1912	KPN_02410	SOG
b1913	KPN_02411	SOG
b1914	KPN_02412	SOG
b1917	KPN_02415	SOG
b1918	KPN_02416	SOG
b1919	KPN_02417	SOG
b1920	KPN_02418	SOG
b1927	KPN_02419	SOG
b1958	KPN_02425	SOG
b1960	KPN_02427	SOG
b1961	KPN_02428	SOG
b1990	KPN_02435	SOG
b1982	KPN_02445	SOG
b1985	KPN_02447	SOG
b1992	KPN_02464	SOG
b2008	KPN_02466	SOG
b2011	KPN_02469	SOG
b2014	KPN_02472	SOG
b2016	KPN_02474	SOG
b2019	KPN_02475	SOG
b2020	KPN_02476	SOG
b2021	KPN_02477	SOG
b2022	KPN_02478	SOG
b2023	KPN_02479	SOG
b2024	KPN_02480	SOG
b2025	KPN_02481	SOG
b2026	KPN_02482	SOG
b2029	KPN_02499	SOG
b2042	KPN_02515	SOG
b2063	KPN_02516	SOG
b2065	KPN_02518	SOG
b2066	KPN_02519	SOG
b2069	KPN_02521	SOG
b2074	KPN_02526	SOG

b1632	STM1454	COG
b1633	STM1453	COG
b1634	STM1452	COG
b1635	STM1451	COG
b1636	STM1450	COG
b1637	STM1449	COG
b1638	STM1448	COG
b1640	STM1446	COG
b1641	STM1445	COG
b1642	STM1444	COG
b1645	STM1441	COG
b1647	STM1439	COG
b1649	STM1437	COG
b1650	STM1436	COG
b1652	STM1434	COG
b1655	STM1432	COG
b1656	STM1431	COG
b1658	STM1430	COG
b1659	STM1429	COG
b1660	STM1428	COG
b1661	STM1427	COG
b1662	STM1426	COG
b1663	STM1425	COG
b1676	STM1378	COG
b1678	STM1375	COG
b1680	STM1373	COG
b1681	STM1372	COG
b1682	STM1371	COG
b1683	STM1370	COG
b1687	STM1365	COG
b1688	STM1364	COG
b1690	STM1361	COG
b1692	STM1359	COG
b1693	STM1358	COG
b1702	STM1349	COG
b1703	STM1348	COG
b1704	STM1347	COG
b1706	STM1345	COG
b1709	STM1342	COG
b1710	STM1341	COG
b1711	STM1340	COG
b1713	STM1338	COG
b1714	STM1337	COG
b1718	STM1334.c	COG
b1719	STM1333	COG
b1722	STM1327	COG

STM1879	KPN_02361	SOG
STM1880	KPN_02362	SOG
STM1884	KPN_02365	SOG
STM1885	KPN_02366	SOG
STM1886	KPN_02367	SOG
STM1887	KPN_02368	SOG
STM1888	KPN_02369	SOG
STM1889	KPN_02370	SOG
STM1890	KPN_02371	SOG
STM1891	KPN_02372	SOG
STM1893	KPN_02374	SOG
STM1894	KPN_02375	SOG
STM1895	KPN_02376	SOG
STM1898	KPN_02377	SOG
STM1899	KPN_02378	SOG
STM1900	KPN_02379	SOG
STM1901	KPN_02380	SOG
STM1903	KPN_02382	SOG
STM1905	KPN_02384	SOG
STM1906	KPN_02385	SOG
STM1907	KPN_02386	SOG
STM1908	KPN_02387	SOG
STM1909	KPN_02388	SOG
STM1927	KPN_02391	SOG
STM1929	KPN_02393	SOG
STM1932	KPN_02397	SOG
STM1935	KPN_02402	SOG
STM1938	KPN_02405	SOG
STM1945	KPN_02410	SOG
STM1946	KPN_02411	SOG
STM1947	KPN_02412	SOG
STM1951	KPN_02415	SOG
STM1952	KPN_02416	SOG
STM1953	KPN_02417	SOG
STM1954	KPN_02418	SOG
STM1963	KPN_02419	SOG
STM1964	KPN_02420	SOG
STM1987	KPN_02424	SOG
STM1989	KPN_02425	SOG
STM1992	KPN_02428	SOG
STM1993	KPN_02429	SOG
STM2015	KPN_02435	SOG
STM3376	KPN_02441	SOG
STM2009	KPN_02445	COG
STM2017	KPN_02464	SOG
STM2060	KPN_02466	SOG

b2075	KPN_02527	SOG
b2076	KPN_02528	SOG
b2077	KPN_02529	SOG
b2079	KPN_02531	SOG
b2081	KPN_02532	SOG
b2086	KPN_02533	SOG
b2097	KPN_02549	SOG
b2103	KPN_02550	SOG
b2104	KPN_02551	SOG
b2113	KPN_02559	SOG
b2114	KPN_02560	SOG
b2124	KPN_02561	SOG
b2125	KPN_02562	SOG
b2126	KPN_02563	SOG
b2129	KPN_02565	SOG
b2131	KPN_02567	SOG
b2132	KPN_02568	SOG
b2133	KPN_02569	SOG
b2134	KPN_02573	SOG
b2140	KPN_02581	SOG
b2142	KPN_02583	SOG
b2144	KPN_02585	SOG
b2148	KPN_02586	SOG
b2149	KPN_02587	SOG
b2150	KPN_02588	SOG
b2151	KPN_02589	SOG
b2152	KPN_02590	SOG
b2153	KPN_02591	SOG
b2154	KPN_02592	SOG
b2155	KPN_02593	SOG
b2156	KPN_02594	SOG
b2157	KPN_02595	SOG
b2158	KPN_02596	SOG
b2159	KPN_02597	SOG
b2167	KPN_02598	SOG
b2168	KPN_02599	SOG
b2169	KPN_02600	SOG
b2170	KPN_02601	SOG
b2171	KPN_02603	SOG
b2173	KPN_02606	SOG
b2175	KPN_02608	SOG
b2178	KPN_02611	SOG
b2180	KPN_02613	SOG
b2182	KPN_02615	SOG
b2183	KPN_02616	SOG
b2184	KPN_02617	SOG

b1723	STM1326	COG
b1725	STM1324	COG
b1726	STM1323	COG
b1727	STM1322	COG
b1728	STM1321	COG
b1729	STM1320	COG
b1732	STM1318	COG
b1733	STM1317	COG
b1734	STM1316	COG
b1735	STM1315	COG
b1737	STM1313	COG
b1740	STM1310	COG
b1741	STM1309	COG
b1743	STM1308	COG
b1744	STM1307	COG
b1745	STM1306	COG
b1747	STM1304	COG
b1748	STM1303	COG
b1749	STM1302	COG
b1761	STM1299	COG
b1763	STM1298	COG
b1764	STM1297	COG
b1765	STM1296	COG
b1766	STM1295	COG
b1767	STM1294	COG
b1779	STM1290	COG
b1780	STM1289	COG
b1782	STM1286	COG
b1783	STM1285	COG
b1784	STM1284	COG
b1787	STM1282	COG
b1790	STM1279	COG
b1791	STM1278	COG
b1804	STM1817	SOG
b1805	STM1818	SOG
b1806	STM1819	SOG
b1807	STM1820	SOG
b1808	STM1821	SOG
b1812	STM1824	SOG
b1813	STM1825	SOG
b1814	STM1826	SOG
b1815	STM1827.S	SOG
b1816	STM1828	SOG
b1817	STM1830	SOG
b1818	STM1831	SOG
b1819	STM1832	SOG

STM2062	KPN_02468	SOG
STM2067	KPN_02469	SOG
STM2068	KPN_02472	SOG
STM2069	KPN_02473	SOG
STM2070	KPN_02474	SOG
STM2071	KPN_02475	SOG
STM2073	KPN_02477	SOG
STM2074	KPN_02478	SOG
STM2075	KPN_02479	SOG
STM2076	KPN_02480	SOG
STM2077	KPN_02481	SOG
STM2078	KPN_02482	SOG
STM2081	KPN_02499	SOG
STM2098	KPN_02515	SOG
STM2119	KPN_02516	SOG
STM2121	KPN_02518	SOG
STM2122	KPN_02519	SOG
STM2125	KPN_02521	SOG
STM2126	KPN_02526	SOG
STM2128	KPN_02528	SOG
STM2131	KPN_02531	SOG
STM2136	KPN_02532	SOG
STM2140	KPN_02533	SOG
STM2141	KPN_02549	SOG
STM2146	KPN_02550	SOG
STM2154	KPN_02559	SOG
STM2155	KPN_02560	SOG
STM2157	KPN_02561	SOG
STM2158	KPN_02562	SOG
STM2159	KPN_02563	SOG
STM2163	KPN_02565	SOG
STM2165	KPN_02567	SOG
STM2166	KPN_02568	SOG
STM2167	KPN_02569	SOG
STM2168	KPN_02573	SOG
STM2169	KPN_02574	SOG
STM2174	KPN_02581	SOG
STM2182	KPN_02583	SOG
STM2183	KPN_02584	SOG
STM2184	KPN_02585	SOG
STM2188	KPN_02586	SOG
STM2189	KPN_02587	SOG
STM2190	KPN_02588	SOG
STM2191	KPN_02589	SOG
STM2192	KPN_02590	SOG
STM2193	KPN_02591	SOG

b2186	KPN_02623	SOG
b2188	KPN_02625	SOG
b2209	KPN_02628	SOG
b2211	KPN_02632	SOG
b2214	KPN_02635	SOG
b2215	KPN_02636	SOG
b2216	KPN_02637	SOG
b2217	KPN_02638	SOG
b2218	KPN_02639	SOG
b2231	KPN_02640	SOG
b2232	KPN_02641	SOG
b2234	KPN_02642	SOG
b2235	KPN_02643	SOG
b2239	KPN_02645	SOG
b2240	KPN_02646	SOG
b2241	KPN_02647	SOG
b2243	KPN_02649	SOG
b2246	KPN_02651	SOG
b2247	KPN_02652	SOG
b2248	KPN_02653	SOG
b2249	KPN_02654	SOG
b2250	KPN_02655	SOG
b2260	KPN_02658	SOG
b2261	KPN_02659	SOG
b2262	KPN_02660	SOG
b2264	KPN_02662	SOG
b2276	KPN_02666	SOG
b2277	KPN_02667	SOG
b2278	KPN_02668	SOG
b2280	KPN_02670	SOG
b2281	KPN_02671	SOG
b2282	KPN_02672	SOG
b2283	KPN_02673	SOG
b2284	KPN_02674	SOG
b2285	KPN_02675	SOG
b2286	KPN_02676	SOG
b2287	KPN_02677	SOG
b2290	KPN_02680	SOG
b2291	KPN_02681	SOG
b2292	KPN_02682	SOG
b2295	KPN_02686	SOG
b2296	KPN_02687	SOG
b2297	KPN_02688	SOG
b2299	KPN_02689	SOG
b2306	KPN_02696	SOG
b2308	KPN_02698	SOG

b1820	STM1833	SOG
b1821	STM1834	SOG
b1822	STM1835	SOG
b1827	STM1842	SOG
b1828	STM1843	SOG
b1829	STM1844	SOG
b1830	STM1845	SOG
b1831	STM1846	SOG
b1832	STM1847	SOG
b1833	STM1848	SOG
b1834	STM1849	SOG
b1835	STM1850	SOG
b1844	STM1878	SOG
b1845	STM1879	SOG
b1846	STM1880	SOG
b1849	STM1883	SOG
b1850	STM1884	SOG
b1851	STM1885	SOG
b1852	STM1886	SOG
b1853	STM1887	SOG
b1854	STM1888	SOG
b1855	STM1889	SOG
b1856	STM1890	SOG
b1857	STM1891	SOG
b1858	STM1892.S	SOG
b1859	STM1893	SOG
b1860	STM1894	SOG
b1861	STM1895	SOG
b1863	STM1898	SOG
b1864	STM1899	SOG
b1865	STM1900	SOG
b1866	STM1901	SOG
b1868	STM1903	SOG
b1870	STM1905	SOG
b1871	STM1906	SOG
b1874	STM1907	SOG
b1875	STM1908	SOG
b1876	STM1909	SOG
b1895	STM1927	SOG
b1896	STM1928	SOG
b1897	STM1929	SOG
b1902	STM1932	SOG
b1905	STM1935	SOG
b1907	STM1937	SOG
b1908	STM1938	SOG
b1912	STM1945	SOG

STM2194	KPN_02592	SOG
STM2199	KPN_02593	SOG
STM2200	KPN_02594	SOG
STM2201	KPN_02595	SOG
STM2202	KPN_02596	SOG
STM2203	KPN_02597	SOG
STM2204	KPN_02598	SOG
STM2205	KPN_02599	SOG
STM2206	KPN_02600	SOG
STM2207	KPN_02601	SOG
STM2211.S	KPN_02603	SOG
STM2214	KPN_02608	SOG
STM2216	KPN_02610	SOG
STM2217	KPN_02611	SOG
STM2218	KPN_02612	SOG
STM2222	KPN_02616	SOG
STM2223	KPN_02617	SOG
STM2226	KPN_02623	SOG
STM2228	KPN_02625	SOG
STM2262	KPN_02628	SOG
STM2263	KPN_02632	SOG
STM2264	KPN_02633	SOG
STM2267	KPN_02636	SOG
STM2269	KPN_02637	SOG
STM2270	KPN_02638	SOG
STM2271	KPN_02639	SOG
STM2272	KPN_02640	SOG
STM2276	KPN_02641	SOG
STM2277	KPN_02642	SOG
STM2278	KPN_02643	SOG
STM2282	KPN_02645	SOG
STM2283	KPN_02646	SOG
STM2286	KPN_02649	SOG
STM2290	KPN_02651	SOG
STM2291	KPN_02652	SOG
STM2292	KPN_02653	SOG
STM2293	KPN_02654	SOG
STM2294	KPN_02655	SOG
STM2306	KPN_02659	SOG
STM2307	KPN_02660	SOG
STM2309	KPN_02662	SOG
STM2312	KPN_02664	SOG
STM2313	KPN_02665	SOG
STM2316.S	KPN_02666	SOG
STM2317	KPN_02667	SOG
STM2318	KPN_02668	SOG

b2309	KPN_02699	SOG
b2310	KPN_02700	SOG
b2311	KPN_02701	SOG
b2312	KPN_02702	SOG
b2313	KPN_02703	SOG
b2314	KPN_02704	SOG
b2315	KPN_02705	SOG
b2316	KPN_02706	SOG
b2318	KPN_02708	SOG
b2319	KPN_02709	SOG
b2320	KPN_02710	SOG
b2321	KPN_02711	SOG
b2323	KPN_02713	SOG
b2326	KPN_02716	SOG
b2327	KPN_02717	SOG
b2328	KPN_02718	SOG
b2329	KPN_02719	SOG
b2331	KPN_02721	SOG
b2340	KPN_02722	SOG
b2341	KPN_02723	SOG
b2342	KPN_02724	SOG
b2344	KPN_02726	SOG
b2346	KPN_02727	SOG
b2347	KPN_02728	SOG
b2379	KPN_02735	SOG
b2388	KPN_02738	SOG
b2392	KPN_02743	SOG
b2393	KPN_02744	SOG
b2400	KPN_02750	SOG
b2411	KPN_02758	SOG
b2412	KPN_02759	SOG
b2413	KPN_02760	SOG
b2414	KPN_02761	SOG
b2416	KPN_02763	SOG
b2417	KPN_02764	SOG
b2421	KPN_02769	SOG
b2422	KPN_02770	SOG
b2423	KPN_02771	SOG
b2424	KPN_02772	SOG
b2425	KPN_02773	SOG
b2431	KPN_02774	SOG
b2432	KPN_02775	SOG
b2434	KPN_02777	SOG
b2435	KPN_02778	SOG
b2436	KPN_02779	SOG
b2437	KPN_02780	SOG

b1913	STM1946	SOG
b1914	STM1947	SOG
b1916	STM1950	SOG
b1917	STM1951	SOG
b1918	STM1952	SOG
b1919	STM1953	SOG
b1920	STM1954	SOG
b1927	STM1963	SOG
b1929	STM1965	SOG
b1931	STM1053	COG
b1951	STM1982	SOG
b1958	STM1989	SOG
b1959	STM1990	SOG
b1960	STM1991	SOG
b1961	STM1992	SOG
b1971	STM3377	SOG
b1972	STM3378	SOG
b1982	STM2009	COG
b1985	STM2013	SOG
b1990	STM2015	SOG
b1992	STM2017	SOG
b1993	STM2018	SOG
b2010	STM2062	SOG
b2011	STM2067	SOG
b2014	STM2068	SOG
b2015	STM2069	SOG
b2016	STM2070	SOG
b2019	STM2071	SOG
b2020	STM2072	SOG
b2021	STM2073	SOG
b2022	STM2074	SOG
b2023	STM2075	SOG
b2024	STM2076	SOG
b2025	STM2077	SOG
b2026	STM2078	SOG
b2029	STM2081	SOG
b2042	STM2098	SOG
b2061	STM2117	SOG
b2063	STM2119	SOG
b2064	STM2120	SOG
b2065	STM2121	SOG
b2066	STM2122	SOG
b2068	STM2124	SOG
b2069	STM2125	SOG
b2074	STM2126	SOG
b2075	STM2127	SOG

STM2320	KPN_02670	SOG
STM2321	KPN_02671	SOG
STM2322	KPN_02672	SOG
STM2323.S	KPN_02673	SOG
STM2324	KPN_02674	SOG
STM2325	KPN_02675	SOG
STM2326	KPN_02676	SOG
STM2327	KPN_02677	SOG
STM2331	KPN_02680	SOG
STM2332	KPN_02681	SOG
STM2333	KPN_02682	SOG
STM2336	KPN_02686	SOG
STM2337	KPN_02687	SOG
STM2338	KPN_02688	SOG
STM2346	KPN_02689	SOG
STM2347	KPN_02690	SOG
STM2348	KPN_02691	SOG
STM2349	KPN_02692	SOG
STM2351	KPN_02696	SOG
STM2353	KPN_02698	SOG
STM2354	KPN_02699	SOG
STM2355	KPN_02700	SOG
STM2362	KPN_02702	SOG
STM2363	KPN_02703	SOG
STM2364	KPN_02704	SOG
STM2365	KPN_02705	SOG
STM2366	KPN_02706	SOG
STM2368	KPN_02708	SOG
STM2369	KPN_02709	SOG
STM2370	KPN_02710	SOG
STM2372	KPN_02712	SOG
STM2378	KPN_02713	SOG
STM2379	KPN_02714	SOG
STM2381	KPN_02716	SOG
STM2383	KPN_02718	SOG
STM2384	KPN_02719	SOG
STM2386	KPN_02721	SOG
STM2387	KPN_02722	SOG
STM2388	KPN_02723	SOG
STM2389	KPN_02724	SOG
STM2391	KPN_02726	SOG
STM2392	KPN_02727	SOG
STM2393	KPN_02728	SOG
STM2402	KPN_02735	SOG
STM2403	KPN_02738	SOG
STM2408	KPN_02743	SOG

b2438	KPN_02781	SOG
b2439	KPN_02782	SOG
b2440	KPN_02783	SOG
b2441	KPN_02784	SOG
b2451	KPN_02785	SOG
b2452	KPN_02786	SOG
b2453	KPN_02787	SOG
b2454	KPN_02788	SOG
b2455	KPN_02789	SOG
b2458	KPN_02792	SOG
b2459	KPN_02793	SOG
b2460	KPN_02794	SOG
b2461	KPN_02795	SOG
b2463	KPN_02797	SOG
b2464	KPN_02798	SOG
b2465	KPN_02799	SOG
b2466	KPN_02800	SOG
b2467	KPN_02801	SOG
b2468	KPN_02802	SOG
b2470	KPN_02803	SOG
b2472	KPN_02805	SOG
b2476	KPN_02810	SOG
b2477	KPN_02811	SOG
b2478	KPN_02812	SOG
b2479	KPN_02813	SOG
b2480	KPN_02814	SOG
b2493	KPN_02815	SOG
b2494	KPN_02816	SOG
b2496	KPN_02819	SOG
b2497	KPN_02820	SOG
b2498	KPN_02821	SOG
b2499	KPN_02824	SOG
b2501	KPN_02826	SOG
b2502	KPN_02827	SOG
b2503	KPN_02828	SOG
b2507	KPN_02833	SOG
b2508	KPN_02834	SOG
b2509	KPN_02835	SOG
b2511	KPN_02841	SOG
b2512	KPN_02842	SOG
b2513	KPN_02843	SOG
b2514	KPN_02844	SOG
b2515	KPN_02845	SOG
b2516	KPN_02846	SOG
b2517	KPN_02847	SOG
b2518	KPN_02848	SOG

b2076	STM2128	SOG
b2077	STM2129	SOG
b2078	STM2130	SOG
b2079	STM2131	SOG
b2081	STM2136	SOG
b2091	STM3261	COG
b2092	STM3260	COG
b2095	STM3257	COG
b2097	STM2141	SOG
b2103	STM2146	SOG
b2106	STM3024	SOG
b2113	STM2154	SOG
b2114	STM2155	SOG
b2124	STM2157	SOG
b2125	STM2158	SOG
b2126	STM2159	SOG
b2128	STM2162	SOG
b2129	STM2163	SOG
b2130	STM2164	SOG
b2131	STM2165	SOG
b2132	STM2166	SOG
b2133	STM2167	SOG
b2134	STM2168	SOG
b2135	STM2169	SOG
b2136	STM2170	SOG
b2140	STM2174	SOG
b2142	STM2182	SOG
b2143	STM2183	SOG
b2144	STM2184	SOG
b2148	STM2188	SOG
b2149	STM2189	SOG
b2150	STM2190	SOG
b2151	STM2191	SOG
b2152	STM2192	SOG
b2153	STM2193	SOG
b2154	STM2194	SOG
b2155	STM2199	SOG
b2156	STM2200	SOG
b2157	STM2201	SOG
b2158	STM2202	SOG
b2159	STM2203	SOG
b2167	STM2204	SOG
b2168	STM2205	SOG
b2169	STM2206	SOG
b2170	STM2207	SOG
b2171	STM2211.S	SOG

STM2409	KPN_02744	SOG
STM2415	KPN_02750	SOG
STM2427	KPN_02758	SOG
STM2428	KPN_02759	SOG
STM2429	KPN_02760	SOG
STM2430	KPN_02761	SOG
STM2432	KPN_02763	SOG
STM2433	KPN_02764	SOG
STM2440	KPN_02769	SOG
STM2441	KPN_02770	SOG
STM2442	KPN_02771	SOG
STM2443	KPN_02772	SOG
STM2444	KPN_02773	SOG
STM2446	KPN_02774	SOG
STM2447	KPN_02775	SOG
STM2448	KPN_02776	SOG
STM2449.S	KPN_02777	SOG
STM2450	KPN_02778	SOG
STM2451	KPN_02779	SOG
STM2454	KPN_02780	SOG
STM2455	KPN_02781	SOG
STM2456	KPN_02782	SOG
STM2457	KPN_02783	SOG
STM2458	KPN_02784	SOG
STM2459	KPN_02785	SOG
STM2460	KPN_02786	SOG
STM2461	KPN_02787	SOG
STM2462	KPN_02788	SOG
STM2463	KPN_02789	SOG
STM2466	KPN_02792	SOG
STM2467	KPN_02793	SOG
STM2468	KPN_02794	SOG
STM2469	KPN_02795	SOG
STM2472	KPN_02797	SOG
STM2473	KPN_02798	SOG
STM2474	KPN_02799	SOG
STM2479	KPN_02802	SOG
STM2481	KPN_02803	SOG
STM2483	KPN_02805	SOG
STM2486	KPN_02809	SOG
STM2487	KPN_02810	SOG
STM2488	KPN_02811	SOG
STM2489	KPN_02812	SOG
STM2490	KPN_02813	SOG
STM2491	KPN_02814	SOG
STM2493	KPN_02815	SOG

b2520	KPN_02850	SOG
b2521	KPN_02851	SOG
b2523	KPN_02855	SOG
b2526	KPN_02858	SOG
b2527	KPN_02859	SOG
b2530	KPN_02862	SOG
b2531	KPN_02863	SOG
b2532	KPN_02864	SOG
b2533	KPN_02865	SOG
b2536	KPN_02870	SOG
b2551	KPN_02876	SOG
b2552	KPN_02877	SOG
b2554	KPN_02879	SOG
b2557	KPN_02882	SOG
b2560	KPN_02885	SOG
b2561	KPN_02886	SOG
b2564	KPN_02889	SOG
b2565	KPN_02890	SOG
b2566	KPN_02891	SOG
b2567	KPN_02892	SOG
b2568	KPN_02893	SOG
b2569	KPN_02894	SOG
b2570	KPN_02895	SOG
b2571	KPN_02896	SOG
b2572	KPN_02897	SOG
b2573	KPN_02898	SOG
b2574	KPN_02899	SOG
b2575	KPN_02900	SOG
b2576	KPN_02901	SOG
b2580	KPN_02903	SOG
b2581	KPN_02904	SOG
b2583	KPN_02906	SOG
b2584	KPN_02907	SOG
b2585	KPN_02908	SOG
b2592	KPN_02915	SOG
b2593	KPN_02916	SOG
b2594	KPN_02917	SOG
b2595	KPN_02918	SOG
b2599	KPN_02920	SOG
b2600	KPN_02922	SOG
b2601	KPN_02923	SOG
b2603	KPN_02924	SOG
b2607	KPN_02930	SOG
b2608	KPN_02931	SOG
b2610	KPN_02933	SOG
b2611	KPN_02934	SOG

b2173	STM2212	SOG
b2175	STM2214	SOG
b2176	STM2215	SOG
b2177	STM2216	SOG
b2178	STM2217	SOG
b2179	STM2218	SOG
b2180	STM2219	SOG
b2182	STM2221	SOG
b2183	STM2222	SOG
b2184	STM2223	SOG
b2186	STM2226	SOG
b2188	STM2228	SOG
b2194	STM3812	SOG
b2195	STM3813	SOG
b2196	STM3814	SOG
b2197	STM3815	SOG
b2199	STM3817	SOG
b2200	STM3818	SOG
b2209	STM2262	SOG
b2211	STM2263	SOG
b2212	STM2264	SOG
b2213	STM2265	SOG
b2214	STM2266	SOG
b2215	STM2267	SOG
b2216	STM2269	SOG
b2217	STM2270	SOG
b2218	STM2271	SOG
b2231	STM2272	SOG
b2232	STM2276	SOG
b2234	STM2277	SOG
b2235	STM2278	SOG
b2239	STM2282	SOG
b2240	STM2283	SOG
b2241	STM2284	SOG
b2243	STM2286	SOG
b2245	STM2289	SOG
b2246	STM2290	SOG
b2247	STM2291	SOG
b2248	STM2292	SOG
b2249	STM2293	SOG
b2250	STM2294	SOG
b2251	STM2295	SOG
b2254	STM2298	SOG
b2255	STM2299	SOG
b2256	STM2300	SOG
b2257	STM2301	SOG

STM2494	KPN_02816	SOG
STM2496	KPN_02819	SOG
STM2497	KPN_02820	SOG
STM2498	KPN_02821	SOG
STM2499.S	KPN_02824	SOG
STM2500	KPN_02825	SOG
STM2501	KPN_02826	SOG
STM2502	KPN_02827	SOG
STM2503	KPN_02828	SOG
STM2510	KPN_02833	SOG
STM2511	KPN_02834	SOG
STM2512	KPN_02835	SOG
STM2519	KPN_02841	SOG
STM2520	KPN_02842	SOG
STM2521	KPN_02843	SOG
STM2522	KPN_02844	SOG
STM2523	KPN_02845	SOG
STM2524	KPN_02846	SOG
STM2525	KPN_02847	SOG
STM2526	KPN_02848	SOG
STM2532	KPN_02850	SOG
STM2533	KPN_02851	SOG
STM2535	KPN_02854	SOG
STM2536	KPN_02855	SOG
STM2539	KPN_02858	SOG
STM2540	KPN_02859	SOG
STM2543	KPN_02862	SOG
STM2544	KPN_02863	SOG
STM2545	KPN_02864	SOG
STM2546	KPN_02865	SOG
STM2554	KPN_02870	SOG
STM2555	KPN_02876	SOG
STM2556	KPN_02877	SOG
STM2562	KPN_02879	SOG
STM2564	KPN_02881	SOG
STM2565	KPN_02882	SOG
STM2567	KPN_02883	SOG
STM2569	KPN_02885	SOG
STM2572	KPN_02886	SOG
STM2578	KPN_02889	SOG
STM2579	KPN_02890	SOG
STM2580	KPN_02891	SOG
STM2581	KPN_02892	SOG
STM2582	KPN_02893	SOG
STM2583	KPN_02894	SOG
STM2637	KPN_02895	SOG

b4461	KPN_02935	SOG
b2614	KPN_02936	SOG
b2615	KPN_02937	SOG
b2616	KPN_02938	SOG
b2619	KPN_02941	SOG
b2620	KPN_02942	SOG
b2675	KPN_03006	SOG
b2677	KPN_03008	SOG
b2678	KPN_03009	SOG
b2679	KPN_03010	SOG
b2685	KPN_03014	SOG
b2686	KPN_03015	SOG
b2687	KPN_03018	SOG
b2688	KPN_03019	SOG
b2689	KPN_03020	SOG
b2697	KPN_03029	SOG
b2698	KPN_03030	SOG
b2699	KPN_03031	SOG
b2700	KPN_03032	SOG
b2701	KPN_03036	SOG
b2702	KPN_03037	SOG
b2705	KPN_03040	SOG
b2707	KPN_03042	SOG
b2708	KPN_03043	SOG
b2709	KPN_03044	SOG
b2710	KPN_03045	SOG
b2713	KPN_03049	SOG
b2717	KPN_03054	SOG
b2719	KPN_03056	SOG
b2720	KPN_03057	SOG
b2721	KPN_03058	SOG
b2722	KPN_03059	SOG
b2723	KPN_03060	SOG
b2724	KPN_03061	SOG
b2725	KPN_03062	SOG
b2727	KPN_03064	SOG
b2729	KPN_03066	SOG
b2730	KPN_03067	SOG
b2731	KPN_03068	SOG
b2733	KPN_03094	SOG
b2741	KPN_03103	SOG
b2742	KPN_03104	SOG
b2743	KPN_03105	SOG
b2744	KPN_03106	SOG
b2745	KPN_03107	SOG
b2746	KPN_03108	SOG

b2260	STM2305	SOG
b2261	STM2306	SOG
b2262	STM2307	SOG
b2263	STM2308	SOG
b2264	STM2309	SOG
b2265	STM2310	SOG
b2268	STM2313	SOG
b2276	STM2316.S	SOG
b2277	STM2317	SOG
b2278	STM2318	SOG
b2280	STM2320	SOG
b2281	STM2321	SOG
b2282	STM2322	SOG
b2283	STM2323.S	SOG
b2284	STM2324	SOG
b2285	STM2325	SOG
b2286	STM2326	SOG
b2287	STM2327	SOG
b2288	STM2328	SOG
b2289	STM2330	SOG
b2290	STM2331	SOG
b2291	STM2332	SOG
b2292	STM2333	SOG
b2293	STM2334	SOG
b2294	STM2335	SOG
b2295	STM2336	SOG
b2296	STM2337	SOG
b2297	STM2338	SOG
b2299	STM2346	SOG
b2300	STM2347	SOG
b2301	STM2348	SOG
b2302	STM2349	SOG
b2304	STM2350	SOG
b2306	STM2351	SOG
b2308	STM2353	SOG
b2309	STM2354	SOG
b2310	STM2355	SOG
b2311	STM2356	SOG
b2312	STM2362	SOG
b2313	STM2363	SOG
b2315	STM2365	SOG
b2316	STM2366	SOG
b2317	STM2367	SOG
b2318	STM2368	SOG
b2319	STM2369	SOG
b2320	STM2370	SOG

STM2638	KPN_02896	SOG
STM2639	KPN_02897	SOG
STM2640	KPN_02898	SOG
STM2641	KPN_02899	SOG
STM2642	KPN_02900	SOG
STM2643	KPN_02901	SOG
STM2647	KPN_02903	SOG
STM2648	KPN_02904	SOG
STM2650	KPN_02906	SOG
STM2651	KPN_02907	SOG
STM2652	KPN_02908	SOG
STM2654	KPN_02910	SOG
STM2660	KPN_02915	SOG
STM2661	KPN_02916	SOG
STM2662	KPN_02917	SOG
STM2663	KPN_02918	SOG
STM2667	KPN_02920	SOG
STM2669	KPN_02922	SOG
STM2670	KPN_02923	SOG
STM2674	KPN_02930	SOG
STM2675	KPN_02931	SOG
STM2677	KPN_02933	SOG
STM2678	KPN_02934	SOG
STM2679	KPN_02935	SOG
STM2681	KPN_02936	SOG
STM2683	KPN_02937	SOG
STM2684	KPN_02938	SOG
STM2687	KPN_02941	SOG
STM2688	KPN_02942	SOG
STM3680	KPN_02991	SOG
STM2798	KPN_02996	SOG
STM2807	KPN_03006	SOG
STM2808	KPN_03007	SOG
STM2809	KPN_03008	SOG
STM2810	KPN_03009	SOG
STM2811	KPN_03010	SOG
STM2813	KPN_03013	SOG
STM2814	KPN_03014	SOG
STM2815	KPN_03015	SOG
STM2817	KPN_03018	SOG
STM2818	KPN_03019	SOG
STM2819	KPN_03020	SOG
STM2820	KPN_03021	SOG
STM2827	KPN_03029	SOG
STM2828	KPN_03030	SOG
STM2829	KPN_03031	SOG

b2747	KPN_03109	SOG
b2750	KPN_03112	SOG
b2751	KPN_03113	SOG
b2752	KPN_03114	SOG
b2762	KPN_03117	SOG
b2763	KPN_03118	SOG
b2764	KPN_03119	SOG
b2777	KPN_03121	SOG
b2779	KPN_03123	SOG
b2780	KPN_03124	SOG
b2781	KPN_03125	SOG
b2784	KPN_03126	SOG
b2785	KPN_03127	SOG
b2786	KPN_03128	SOG
b2787	KPN_03130	SOG
b2788	KPN_03131	SOG
b2789	KPN_03132	SOG
b2790	KPN_03133	SOG
b2791	KPN_03134	SOG
b2793	KPN_03136	SOG
b2794	KPN_03137	SOG
b2795	KPN_03138	SOG
b2796	KPN_03139	SOG
b2797	KPN_03140	SOG
b2800	KPN_03151	SOG
b2802	KPN_03153	SOG
b2803	KPN_03154	SOG
b2804	KPN_03155	SOG
b2805	KPN_03156	SOG
b2806	KPN_03157	SOG
b2808	KPN_03159	SOG
b2810	KPN_03161	SOG
b2811	KPN_03162	SOG
b2812	KPN_03163	SOG
b2813	KPN_03164	SOG
b1993	KPN_03183	SOG
b2817	KPN_03225	SOG
b2818	KPN_03226	SOG
b2820	KPN_03229	SOG
b2821	KPN_03230	SOG
b2822	KPN_03231	SOG
b2827	KPN_03236	SOG
b2828	KPN_03237	SOG
b2829	KPN_03238	SOG
b2830	KPN_03239	SOG
b2831	KPN_03240	SOG

b2322	STM2372	SOG
b2323	STM2378	SOG
b2324	STM2379	SOG
b2326	STM2381	SOG
b2327	STM2382	SOG
b2328	STM2383	SOG
b2329	STM2384	SOG
b2331	STM2386	SOG
b2340	STM2387	SOG
b2341	STM2388	SOG
b2342	STM2389	SOG
b2344	STM2391	SOG
b2346	STM2392	SOG
b2347	STM2393	SOG
b2364	STM3800	SOG
b2365	STM3801	SOG
b2366	STM3802	SOG
b2378	STM2401	SOG
b2379	STM2402	SOG
b2388	STM2403	SOG
b2389	STM2404	SOG
b2392	STM2408	SOG
b2393	STM2409	SOG
b2395	STM2410	SOG
b2400	STM2415	SOG
b2410	STM2425	SOG
b2411	STM2427	SOG
b2412	STM2428	SOG
b2413	STM2429	SOG
b2414	STM2430	SOG
b2416	STM2432	SOG
b2417	STM2433	SOG
b2421	STM2440	SOG
b2422	STM2441	SOG
b2423	STM2442	SOG
b2424	STM2443	SOG
b2425	STM2444	SOG
b2431	STM2446	SOG
b2432	STM2447	SOG
b2433	STM2448	SOG
b2434	STM2449.S	SOG
b2435	STM2450	SOG
b2436	STM2451	SOG
b2437	STM2454	SOG
b2438	STM2455	SOG
b2439	STM2456	SOG

STM2830	KPN_03032	SOG
STM2831	KPN_03036	SOG
STM2832	KPN_03037	SOG
STM2833	KPN_03038	SOG
STM2835	KPN_03040	SOG
STM2837	KPN_03042	SOG
STM2838.S	KPN_03043	SOG
STM2839	KPN_03044	SOG
STM2840	KPN_03045	SOG
STM2841	KPN_03046	SOG
STM2843	KPN_03049	SOG
STM2845	KPN_03054	SOG
STM2847	KPN_03056	SOG
STM2848	KPN_03057	SOG
STM2849	KPN_03058	SOG
STM2850	KPN_03059	SOG
STM2851	KPN_03060	SOG
STM2852	KPN_03061	SOG
STM2853	KPN_03062	SOG
STM2855	KPN_03064	SOG
STM2857	KPN_03066	SOG
STM2909	KPN_03094	SOG
STM2924	KPN_03103	SOG
STM2925	KPN_03104	SOG
STM2926	KPN_03105	SOG
STM2927	KPN_03106	SOG
STM2928	KPN_03107	SOG
STM2929	KPN_03108	SOG
STM2930	KPN_03109	SOG
STM2933	KPN_03112	SOG
STM2934	KPN_03113	SOG
STM2935	KPN_03114	SOG
STM2946	KPN_03117	SOG
STM2947	KPN_03118	SOG
STM2948	KPN_03119	SOG
STM2951	KPN_03121	SOG
STM2952	KPN_03123	SOG
STM2953	KPN_03124	SOG
STM2954	KPN_03125	SOG
STM2956	KPN_03126	SOG
STM2957	KPN_03127	SOG
STM2958	KPN_03128	SOG
STM2960	KPN_03130	SOG
STM2961	KPN_03131	SOG
STM2962	KPN_03132	SOG
STM2963	KPN_03133	SOG

b2832	KPN_03241	SOG
b2834	KPN_03243	SOG
b2835	KPN_03244	SOG
b2836	KPN_03245	SOG
b2837	KPN_03246	SOG
b2838	KPN_03252	SOG
b2840	KPN_03254	SOG
b2841	KPN_03264	SOG
b2842	KPN_03265	SOG
b2889	KPN_03317	SOG
b2890	KPN_03318	SOG
b2891	KPN_03319	SOG
b2892	KPN_03320	SOG
b2893	KPN_03321	SOG
b2894	KPN_03322	SOG
b2895	KPN_03323	SOG
b2898	KPN_03326	SOG
b2899	KPN_03327	SOG
b2901	KPN_03330	SOG
b2903	KPN_03339	SOG
b2905	KPN_03341	SOG
b2906	KPN_03342	SOG
b2908	KPN_03344	SOG
b2909	KPN_03345	SOG
b2913	KPN_03348	SOG
b2914	KPN_03349	SOG
b2916	KPN_03350	SOG
b2923	KPN_03352	SOG
b2924	KPN_03353	SOG
b2925	KPN_03354	SOG
b2926	KPN_03355	SOG
b2927	KPN_03356	SOG
b2935	KPN_03357	SOG
b2936	KPN_03358	SOG
b2937	KPN_03373	SOG
b2938	KPN_03374	SOG
b2942	KPN_03375	SOG
b2943	KPN_03376	SOG
b2944	KPN_03377	SOG
b2945	KPN_03378	SOG
b2946	KPN_03379	SOG
b2947	KPN_03380	SOG
b2948	KPN_03381	SOG
b2950	KPN_03384	SOG
b2951	KPN_03385	SOG
b2952	KPN_03386	SOG

b2440	STM2457	SOG
b2441	STM2458	SOG
b2451	STM2459	SOG
b2452	STM2460	SOG
b2453	STM2461	SOG
b2454	STM2462	SOG
b2455	STM2463	SOG
b2458	STM2466	SOG
b2459	STM2467	SOG
b2460	STM2468	SOG
b2461	STM2469	SOG
b2463	STM2472	SOG
b2464	STM2473	SOG
b2465	STM2474	SOG
b2466	STM2476	SOG
b2467	STM2477	SOG
b2468	STM2479	SOG
b2470	STM2481	SOG
b2472	STM2483	SOG
b2474	STM2485	SOG
b2475	STM2486	SOG
b2476	STM2487	SOG
b2477	STM2488	SOG
b2478	STM2489	SOG
b2479	STM2490	SOG
b2480	STM2491	SOG
b2493	STM2493	SOG
b2494	STM2494	SOG
b2496	STM2496	SOG
b2497	STM2497	SOG
b2498	STM2498	SOG
b2499	STM2499.S	SOG
b2500	STM2500	SOG
b2501	STM2501	SOG
b2502	STM2502	SOG
b2503	STM2503	SOG
b2507	STM2510	SOG
b2508	STM2511	SOG
b2509	STM2512	SOG
b2511	STM2519	SOG
b2512	STM2520	SOG
b2513	STM2521	SOG
b2514	STM2522	SOG
b2515	STM2523	SOG
b2516	STM2524	SOG
b2517	STM2525	SOG

STM2964	KPN_03134	SOG
STM2967	KPN_03136	SOG
STM2968	KPN_03137	SOG
STM2969	KPN_03138	SOG
STM2970	KPN_03139	SOG
STM2971	KPN_03140	SOG
STM2973	KPN_03150	SOG
STM2974	KPN_03151	SOG
STM2976	KPN_03153	SOG
STM2977	KPN_03154	SOG
STM2978	KPN_03155	SOG
STM2979	KPN_03156	SOG
STM2980	KPN_03157	SOG
STM2982	KPN_03159	SOG
STM2985	KPN_03162	SOG
STM2987	KPN_03163	SOG
STM2988	KPN_03164	SOG
STM2018	KPN_03183	SOG
STM2991	KPN_03225	SOG
STM2992	KPN_03226	SOG
STM2994	KPN_03229	SOG
STM2995	KPN_03230	SOG
STM2996	KPN_03231	SOG
STM3001	KPN_03236	SOG
STM3002	KPN_03237	SOG
STM3003	KPN_03238	SOG
STM3004	KPN_03239	SOG
STM3006	KPN_03241	SOG
STM3008	KPN_03243	SOG
STM3009	KPN_03244	SOG
STM3010	KPN_03245	SOG
STM3011	KPN_03246	SOG
STM3013	KPN_03252	SOG
STM3014	KPN_03253	SOG
STM3016	KPN_03264	SOG
STM3017	KPN_03265	SOG
STM3039	KPN_03317	SOG
STM3040	KPN_03318	SOG
STM3041c	KPN_03319	SOG
STM3042	KPN_03320	SOG
STM3043	KPN_03321	SOG
STM3044	KPN_03322	SOG
STM3045	KPN_03323	SOG
STM3048	KPN_03326	SOG
STM3049	KPN_03327	SOG
STM3051	KPN_03330	SOG

b2954	KPN_03388	SOG	b2518	STM2526	SOG	STM3053	KPN_03339	SOG
b2955	KPN_03389	SOG	b2519	STM2531	SOG	STM3055	KPN_03341	SOG
b2958	KPN_03390	SOG	b2520	STM2532	SOG	STM3056	KPN_03342	SOG
b2960	KPN_03392	SOG	b2521	STM2533	SOG	STM3058	KPN_03344	SOG
b2961	KPN_03393	SOG	b2522	STM2535	SOG	STM3059.S	KPN_03345	SOG
b2963	KPN_03395	SOG	b2523	STM2536	SOG	STM3062	KPN_03348	SOG
b2964	KPN_03396	SOG	b2526	STM2539	SOG	STM3063	KPN_03349	SOG
b2965	KPN_03397	SOG	b2527	STM2540	SOG	STM3064	KPN_03350	SOG
b2988	KPN_03416	SOG	b2530	STM2543	SOG	STM3065	KPN_03351	SOG
b2989	KPN_03419	SOG	b2531	STM2544	SOG	STM3066	KPN_03352	SOG
b3005	KPN_03425	SOG	b2532	STM2545	SOG	STM3067	KPN_03353	SOG
b3006	KPN_03426	SOG	b2533	STM2546	SOG	STM3068	KPN_03354	SOG
b3008	KPN_03428	SOG	b2535	STM2553	SOG	STM3069	KPN_03355	SOG
b3009	KPN_03429	SOG	b2536	STM2554	SOG	STM3070	KPN_03356	SOG
b3010	KPN_03430	SOG	b2551	STM2555	SOG	STM3076	KPN_03357	SOG
b3011	KPN_03431	SOG	b2552	STM2556	SOG	STM3078	KPN_03373	SOG
b3012	KPN_03432	SOG	b2554	STM2562	SOG	STM3086	KPN_03374	SOG
b4469	KPN_03434	SOG	b2556	STM2564	SOG	STM3090	KPN_03375	SOG
b3017	KPN_03435	SOG	b2557	STM2565	SOG	STM3091	KPN_03376	SOG
b3018	KPN_03436	SOG	b2558	STM2567	SOG	STM3093	KPN_03378	SOG
b3019	KPN_03437	SOG	b2559	STM2568	SOG	STM3094	KPN_03379	SOG
b3023	KPN_03438	SOG	b2560	STM2569	SOG	STM3095	KPN_03380	SOG
b3026	KPN_03441	SOG	b2561	STM2572	SOG	STM3096	KPN_03381	SOG
b3028	KPN_03442	SOG	b2564	STM2578	SOG	STM3100	KPN_03385	SOG
b3030	KPN_03444	SOG	b2565	STM2579	SOG	STM3101	KPN_03386	SOG
b3031	KPN_03445	SOG	b2566	STM2580	SOG	STM3103	KPN_03388	SOG
b3032	KPN_03446	SOG	b2567	STM2581	SOG	STM3104	KPN_03389	SOG
b3033	KPN_03447	SOG	b2568	STM2582	SOG	STM3107	KPN_03390	SOG
b3034	KPN_03448	SOG	b2569	STM2583	SOG	STM3109	KPN_03392	SOG
b3035	KPN_03449	SOG	b2570	STM2637	SOG	STM3110	KPN_03393	SOG
b3037	KPN_03450	SOG	b2571	STM2638	SOG	STM3112	KPN_03395	SOG
b3038	KPN_03451	SOG	b2572	STM2639	SOG	STM3113	KPN_03396	SOG
b3041	KPN_03454	SOG	b2573	STM2640	SOG	STM3114	KPN_03397	SOG
b3052	KPN_03456	SOG	b2574	STM2641	SOG	STM3115	KPN_03398	SOG
b3053	KPN_03457	SOG	b2575	STM2642	SOG	STM3139	KPN_03416	SOG
b3055	KPN_03459	SOG	b2576	STM2643	SOG	STM3140	KPN_03419	SOG
b3056	KPN_03460	SOG	b2578	STM2645	SOG	STM3153	KPN_03422	SOG
b3057	KPN_03461	SOG	b2580	STM2647	SOG	STM3158	KPN_03425	SOG
b3064	KPN_03471	SOG	b2581	STM2648	SOG	STM3159	KPN_03426	SOG
b3066	KPN_03473	SOG	b2583	STM2650	SOG	STM3162	KPN_03429	SOG
b3067	KPN_03474	SOG	b2584	STM2651	SOG	STM3163	KPN_03430	SOG
b3068	KPN_03475	SOG	b2585	STM2652	SOG	STM3164	KPN_03431	SOG
b3070	KPN_03499	SOG	b2592	STM2660	SOG	STM3165	KPN_03432	SOG
b3073	KPN_03501	SOG	b2593	STM2661	SOG	STM3168	KPN_03434	SOG
b1517	KPN_03503	COG	b2594	STM2662	SOG	STM3172	KPN_03435	SOG
b3081	KPN_03510	SOG	b2595	STM2663	SOG	STM3173	KPN_03436	SOG

b3084	KPN_03511	SOG
b3085	KPN_03514	SOG
b3087	KPN_03515	SOG
b3088	KPN_03516	SOG
b3089	KPN_03517	SOG
b3092	KPN_03520	SOG
b3102	KPN_03530	SOG
b3105	KPN_03532	SOG
b3106	KPN_03533	SOG
b3124	KPN_03536	SOG
b3125	KPN_03537	SOG
b3126	KPN_03538	SOG
b3128	KPN_03540	SOG
b2092	KPN_03550	COG
b3146	KPN_03554	SOG
b3147	KPN_03555	SOG
b3149	KPN_03557	SOG
b3150	KPN_03558	SOG
b3156	KPN_03564	SOG
b3157	KPN_03565	SOG
b3159	KPN_03567	SOG
b3160	KPN_03568	SOG
b3161	KPN_03569	SOG
b3162	KPN_03570	SOG
b3163	KPN_03571	SOG
b3164	KPN_03572	SOG
b3166	KPN_03574	SOG
b3168	KPN_03576	SOG
b3169	KPN_03577	SOG
b3170	KPN_03578	SOG
b3172	KPN_03580	SOG
b3176	KPN_03586	SOG
b3177	KPN_03587	SOG
b3178	KPN_03588	SOG
b3179	KPN_03589	SOG
b3181	KPN_03591	SOG
b3182	KPN_03592	SOG
b3183	KPN_03593	SOG
b3187	KPN_03597	SOG
b3189	KPN_03599	SOG
b3192	KPN_03602	SOG
b3193	KPN_03603	SOG
b3194	KPN_03604	SOG
b3195	KPN_03605	SOG
b3196	KPN_03606	SOG
b3197	KPN_03607	SOG

b2599	STM2667	SOG
b2600	STM2669	SOG
b2601	STM2670	SOG
b2603	STM2671	SOG
b2607	STM2674	SOG
b2608	STM2675	SOG
b2610	STM2677	SOG
b2611	STM2678	SOG
b2614	STM2681	SOG
b2615	STM2683	SOG
b2616	STM2684	SOG
b2620	STM2688	SOG
b2659	STM2789	SOG
b2660	STM2790	SOG
b2661	STM2791	SOG
b2662	STM2792	SOG
b2663	STM2793	SOG
b2664	STM2794	SOG
b2665	STM2795	SOG
b2670	STM2800	SOG
b2675	STM2807	SOG
b2676	STM2808	SOG
b2677	STM2809	SOG
b2678	STM2810	SOG
b2679	STM2811	SOG
b2684	STM2813	SOG
b2685	STM2814	SOG
b2686	STM2815	SOG
b2687	STM2817	SOG
b2688	STM2818	SOG
b2689	STM2819	SOG
b2690	STM2820	SOG
b2697	STM2827	SOG
b2698	STM2828	SOG
b2699	STM2829	SOG
b2700	STM2830	SOG
b2701	STM2831	SOG
b2702	STM2832	SOG
b2703	STM2833	SOG
b2705	STM2835	SOG
b2707	STM2837	SOG
b2708	STM2838.S	SOG
b2709	STM2839	SOG
b2710	STM2840	SOG
b2711	STM2841	SOG
b2712	STM2842	SOG

STM3174	KPN_03437	SOG
STM3175	KPN_03438	SOG
STM3178	KPN_03441	SOG
STM3179	KPN_03442	SOG
STM3181	KPN_03444	SOG
STM3182	KPN_03445	SOG
STM3183	KPN_03446	SOG
STM3184	KPN_03447	SOG
STM3185	KPN_03448	SOG
STM3186	KPN_03449	SOG
STM3187	KPN_03450	SOG
STM3188	KPN_03451	SOG
STM3190	KPN_03453	SOG
STM3195	KPN_03454	SOG
STM3200	KPN_03456	SOG
STM3201	KPN_03457	SOG
STM3203	KPN_03459	SOG
STM3204	KPN_03460	SOG
STM3208	KPN_03471	SOG
STM3210	KPN_03473	SOG
STM3211.S	KPN_03474	SOG
STM3212	KPN_03475	SOG
STM3218	KPN_03501	SOG
STM4077	KPN_03504	COG
STM3219	KPN_03510	SOG
STM3220	KPN_03511	SOG
STM3223	KPN_03515	SOG
STM3224	KPN_03516	SOG
STM3225	KPN_03517	SOG
STM3226	KPN_03523	SOG
STM3233	KPN_03530	SOG
STM3235	KPN_03532	SOG
STM3236	KPN_03533	SOG
STM3247	KPN_03536	SOG
STM3248	KPN_03537	SOG
STM3249	KPN_03538	SOG
STM3250	KPN_03540	SOG
STM3260	KPN_03550	SOG
STM3263	KPN_03554	SOG
STM3264	KPN_03555	SOG
STM3266	KPN_03557	SOG
STM3267	KPN_03558	SOG
STM3270	KPN_03562	SOG
STM3272	KPN_03564	SOG
STM3273	KPN_03565	SOG
STM3275.S	KPN_03567	SOG

b3198	KPN_03608	SOG
b3199	KPN_03609	SOG
b3200	KPN_03610	SOG
b3201	KPN_03611	SOG
b3202	KPN_03612	SOG
b3205	KPN_03615	SOG
b3208	KPN_03617	SOG
b3209	KPN_03618	SOG
b3210	KPN_03619	SOG
b3211	KPN_03623	SOG
b3213	KPN_03625	SOG
b3228	KPN_03627	SOG
b3229	KPN_03628	SOG
b3231	KPN_03630	SOG
b3232	KPN_03631	SOG
b3234	KPN_03633	SOG
b3236	KPN_03644	SOG
b3237	KPN_03645	SOG
b3240	KPN_03649	SOG
b3241	KPN_03650	SOG
b3243	KPN_03652	SOG
b3244	KPN_03653	SOG
b4472	KPN_03654	SOG
b3247	KPN_03655	SOG
b3248	KPN_03656	SOG
b3249	KPN_03657	SOG
b3250	KPN_03658	SOG
b3251	KPN_03659	SOG
b3252	KPN_03660	SOG
b1971	KPN_03661	SOG
b3255	KPN_03664	SOG
b3256	KPN_03665	SOG
b3258	KPN_03667	SOG
b3259	KPN_03668	SOG
b3260	KPN_03669	SOG
b3266	KPN_03673	SOG
b3283	KPN_03684	SOG
b3284	KPN_03685	SOG
b4473	KPN_03686	SOG
b3287	KPN_03687	SOG
b3288	KPN_03688	SOG
b3289	KPN_03689	SOG
b3290	KPN_03690	SOG
b3292	KPN_03692	SOG
b3295	KPN_03695	SOG
b3296	KPN_03696	SOG

b2713	STM2843	SOG
b2717	STM2845	SOG
b2719	STM2847	SOG
b2720	STM2848	SOG
b2721	STM2849	SOG
b2722	STM2850	SOG
b2723	STM2851	SOG
b2724	STM2852	SOG
b2725	STM2853	SOG
b2727	STM2855	SOG
b2729	STM2857	SOG
b2730	STM2858	SOG
b2731	STM2859	SOG
b2733	STM2909	SOG
b2735	STM2919	COG
b2737	STM2917	COG
b2738	STM2916	COG
b2741	STM2924	SOG
b2742	STM2925	SOG
b2743	STM2926	SOG
b2744	STM2927	SOG
b2745	STM2928	SOG
b2746	STM2929	SOG
b2747	STM2930	SOG
b2750	STM2933	SOG
b2751	STM2934	SOG
b2752	STM2935	SOG
b2753	STM2936	SOG
b2762	STM2946	SOG
b2763	STM2947	SOG
b2764	STM2948	SOG
b2777	STM2951	SOG
b2779	STM2952	SOG
b2780	STM2953	SOG
b2781	STM2954	SOG
b2784	STM2956	SOG
b2785	STM2957	SOG
b2786	STM2958	SOG
b2787	STM2960	SOG
b2788	STM2961	SOG
b2789	STM2962	SOG
b2790	STM2963	SOG
b2791	STM2964	SOG
b2793	STM2967	SOG
b2794	STM2968	SOG
b2795	STM2969	SOG

STM3276	KPN_03568	SOG
STM3279	KPN_03569	SOG
STM3280.S	KPN_03570	SOG
STM3281	KPN_03571	SOG
STM3282	KPN_03572	SOG
STM3284	KPN_03574	SOG
STM3286	KPN_03576	SOG
STM3287	KPN_03577	SOG
STM3288	KPN_03578	SOG
STM3290.S	KPN_03580	SOG
STM3294	KPN_03586	SOG
STM3295	KPN_03587	SOG
STM3296	KPN_03588	SOG
STM3297	KPN_03589	SOG
STM3299	KPN_03591	SOG
STM3300	KPN_03592	SOG
STM3301	KPN_03593	SOG
STM3305	KPN_03597	SOG
STM3307	KPN_03599	SOG
STM3310	KPN_03602	SOG
STM3311	KPN_03603	SOG
STM3312	KPN_03604	SOG
STM3313	KPN_03605	SOG
STM3314	KPN_03606	SOG
STM3315	KPN_03607	SOG
STM3316	KPN_03608	SOG
STM3317	KPN_03609	SOG
STM3318	KPN_03610	SOG
STM3319	KPN_03611	SOG
STM3320	KPN_03612	SOG
STM3322	KPN_03614	SOG
STM3323	KPN_03615	SOG
STM3326	KPN_03617	SOG
STM3327	KPN_03618	SOG
STM3328	KPN_03619	SOG
STM3329	KPN_03623	SOG
STM3331	KPN_03625	SOG
STM3337	KPN_03626	SOG
STM3341	KPN_03627	SOG
STM3342	KPN_03628	SOG
STM3345	KPN_03630	SOG
STM3346	KPN_03631	SOG
STM3348	KPN_03633	SOG
STM3349	KPN_03634	SOG
STM3359	KPN_03644	SOG
STM3360	KPN_03645	SOG

b3300	KPN_03699	SOG
b3301	KPN_03700	SOG
b3303	KPN_03702	SOG
b3305	KPN_03704	SOG
b3308	KPN_03707	SOG
b3314	KPN_03713	SOG
b3317	KPN_03716	SOG
b3319	KPN_03718	SOG
b3320	KPN_03719	SOG
b3340	KPN_03725	SOG
b3341	KPN_03726	SOG
b3346	KPN_03731	SOG
b3347	KPN_03732	SOG
b3349	KPN_03734	SOG
b3350	KPN_03736	SOG
b3351	KPN_03737	SOG
b3352	KPN_03738	SOG
b3353	KPN_03739	SOG
b3355	KPN_03741	SOG
b3357	KPN_03743	SOG
b3358	KPN_03744	SOG
b3360	KPN_03746	SOG
b3361	KPN_03747	SOG
b3363	KPN_03749	SOG
b3364	KPN_03750	SOG
b3384	KPN_03755	SOG
b3386	KPN_03757	SOG
b3387	KPN_03758	SOG
b3388	KPN_03759	SOG
b3389	KPN_03760	SOG
b3390	KPN_03761	SOG
b3396	KPN_03767	SOG
b3397	KPN_03768	SOG
b3398	KPN_03769	SOG
b3399	KPN_03770	SOG
b3401	KPN_03772	SOG
b3403	KPN_03773	SOG
b3404	KPN_03774	SOG
b3405	KPN_03775	SOG
b3407	KPN_03777	SOG
b3409	KPN_03779	SOG
b3413	KPN_03783	SOG
b3414	KPN_03784	SOG
b3416	KPN_03786	SOG
b3417	KPN_03787	SOG
b3418	KPN_03788	SOG

b2796	STM2970	SOG
b2797	STM2971	SOG
b2798	STM2972	SOG
b2799	STM2973	SOG
b2800	STM2974	SOG
b2802	STM2976	SOG
b2803	STM2977	SOG
b2804	STM2978	SOG
b2805	STM2979	SOG
b2806	STM2980	SOG
b2808	STM2982	SOG
b2810	STM2984	SOG
b2811	STM2985	SOG
b2812	STM2987	SOG
b2813	STM2988	SOG
b2817	STM2991	SOG
b2818	STM2992	SOG
b2819	STM2993	SOG
b2820	STM2994	SOG
b2821	STM2995	SOG
b2822	STM2996	SOG
b2825	STM2999	SOG
b2826	STM3000	SOG
b2827	STM3001	SOG
b2828	STM3002	SOG
b2829	STM3003	SOG
b2830	STM3004	SOG
b2831	STM3005	SOG
b2832	STM3006	SOG
b2834	STM3008	SOG
b2835	STM3009	SOG
b2836	STM3010	SOG
b2837	STM3011	SOG
b2838	STM3013	SOG
b2839	STM3014	SOG
b2841	STM3016	SOG
b2842	STM3017	SOG
b2844	STM3019	SOG
b2865	STM3038	SOG
b2871	STM1002	SOG
b2889	STM3039	SOG
b2890	STM3040	SOG
b2891	STM3041c	SOG
b2892	STM3042	SOG
b2893	STM3043	SOG
b2894	STM3044	SOG

STM3364	KPN_03649	SOG
STM3365	KPN_03650	SOG
STM3367	KPN_03652	SOG
STM3368	KPN_03653	SOG
STM3369	KPN_03654	SOG
STM3370	KPN_03655	SOG
STM3371	KPN_03656	SOG
STM3372	KPN_03657	SOG
STM3373	KPN_03658	SOG
STM3374	KPN_03659	SOG
STM3375	KPN_03660	SOG
STM3378	KPN_03662	SOG
STM3379	KPN_03664	SOG
STM3380	KPN_03665	SOG
STM3382	KPN_03667	SOG
STM3383	KPN_03668	SOG
STM3384	KPN_03669	SOG
STM3402	KPN_03683	SOG
STM3403	KPN_03684	SOG
STM3404	KPN_03685	SOG
STM3405	KPN_03686	SOG
STM3406	KPN_03687	SOG
STM3407	KPN_03688	SOG
STM3408	KPN_03689	SOG
STM3412	KPN_03692	SOG
STM3415	KPN_03695	SOG
STM3416	KPN_03696	SOG
STM3420	KPN_03699	SOG
STM3421	KPN_03700	SOG
STM3423	KPN_03702	SOG
STM3425	KPN_03704	SOG
STM3428	KPN_03707	SOG
STM3434	KPN_03713	SOG
STM3437	KPN_03716	SOG
STM3439	KPN_03718	SOG
STM3440	KPN_03719	SOG
STM3446	KPN_03725	SOG
STM3447	KPN_03726	SOG
STM3452	KPN_03731	SOG
STM3453	KPN_03732	SOG
STM3455	KPN_03734	SOG
STM3457	KPN_03736	SOG
STM3458	KPN_03737	SOG
STM3459	KPN_03738	SOG
STM3462	KPN_03739	SOG
STM3464	KPN_03741	SOG

b3423	KPN_03789	SOG
b3424	KPN_03790	SOG
b3428	KPN_03794	SOG
b3429	KPN_03795	SOG
b3430	KPN_03796	SOG
b3431	KPN_03797	SOG
b3432	KPN_03798	SOG
b3433	KPN_03799	SOG
b4476	KPN_03800	SOG
b3437	KPN_03801	SOG
b3438	KPN_03802	SOG
b3439	KPN_03803	SOG
b3440	KPN_03804	SOG
b3441	KPN_03806	SOG
b3447	KPN_03807	SOG
b3449	KPN_03809	SOG
b3450	KPN_03810	SOG
b3451	KPN_03811	SOG
b3452	KPN_03812	SOG
b3453	KPN_03813	SOG
b3454	KPN_03816	SOG
b3455	KPN_03817	SOG
b3456	KPN_03818	SOG
b3457	KPN_03819	SOG
b3458	KPN_03820	SOG
b3460	KPN_03822	SOG
b3461	KPN_03827	SOG
b3462	KPN_03828	SOG
b3463	KPN_03829	SOG
b3464	KPN_03830	SOG
b3468	KPN_03834	SOG
b3471	KPN_03837	SOG
b3472	KPN_03838	SOG
b3473	KPN_03839	SOG
b3474	KPN_03840	SOG
b2255	KPN_03845	COG
b2254	KPN_03846	COG
b3492	KPN_03857	SOG
b3493	KPN_03858	SOG
b3495	KPN_03860	SOG
b3496	KPN_03861	SOG
b3497	KPN_03862	SOG
b3498	KPN_03863	SOG
b3499	KPN_03865	SOG
b3500	KPN_03866	SOG
b3519	KPN_03869	SOG

b2895	STM3045	SOG
b2898	STM3048	SOG
b2899	STM3049	SOG
b2901	STM3051	SOG
b2903	STM3053	SOG
b2905	STM3055	SOG
b2906	STM3056	SOG
b2907	STM3057	SOG
b2908	STM3058	SOG
b2909	STM3059.S	SOG
b2913	STM3062	SOG
b2914	STM3063	SOG
b2916	STM3064	SOG
b2922	STM3065	SOG
b2923	STM3066	SOG
b2924	STM3067	SOG
b2925	STM3068	SOG
b2926	STM3069	SOG
b2927	STM3070	SOG
b2935	STM3076	SOG
b2936	STM3077	SOG
b2937	STM3078	SOG
b2938	STM3086	SOG
b2942	STM3090	SOG
b2943	STM3091	SOG
b2944	STM3092	SOG
b2945	STM3093	SOG
b2946	STM3094	SOG
b2947	STM3095	SOG
b2948	STM3096	SOG
b2950	STM3099	SOG
b2951	STM3100	SOG
b2952	STM3101	SOG
b2954	STM3103	SOG
b2955	STM3104	SOG
b2958	STM3107	SOG
b2960	STM3109	SOG
b2961	STM3110	SOG
b2963	STM3112	SOG
b2964	STM3113	SOG
b2965	STM3114	SOG
b2966	STM3115	SOG
b2988	STM3139	SOG
b2989	STM3140	SOG
b3002	STM3153	SOG
b3003	STM3157	SOG

STM3466	KPN_03743	SOG
STM3467	KPN_03744	SOG
STM3470	KPN_03747	SOG
STM3472	KPN_03749	SOG
STM3473	KPN_03750	SOG
STM3477	KPN_03754	SOG
STM3481	KPN_03755	SOG
STM3482	KPN_03756	SOG
STM3483	KPN_03757	SOG
STM3484	KPN_03758	SOG
STM3485	KPN_03759	SOG
STM3486	KPN_03760	SOG
STM3487	KPN_03761	SOG
STM3493	KPN_03767	SOG
STM3494.S	KPN_03768	SOG
STM3495	KPN_03769	SOG
STM3496	KPN_03770	SOG
STM3498	KPN_03772	SOG
STM3500	KPN_03773	SOG
STM3501	KPN_03774	SOG
STM3502	KPN_03775	SOG
STM3504	KPN_03777	SOG
STM3506	KPN_03779	SOG
STM3509	KPN_03782	SOG
STM3510	KPN_03783	SOG
STM3511	KPN_03784	SOG
STM3513	KPN_03786	SOG
STM3514	KPN_03787	SOG
STM3515	KPN_03788	SOG
STM3523	KPN_03789	SOG
STM3524	KPN_03790	SOG
STM3534	KPN_03794	SOG
STM3535	KPN_03795	SOG
STM3536	KPN_03796	SOG
STM3537	KPN_03797	SOG
STM3538	KPN_03798	SOG
STM3539	KPN_03799	SOG
STM3541	KPN_03800	SOG
STM3542	KPN_03801	SOG
STM3543	KPN_03802	SOG
STM3544	KPN_03803	SOG
STM3545	KPN_03804	SOG
STM3546	KPN_03806	SOG
STM3551	KPN_03807	SOG
STM3553	KPN_03809	SOG
STM3554	KPN_03810	SOG

b3521	KPN_03872	SOG
b3522	KPN_03873	SOG
b3523	KPN_03874	SOG
b3526	KPN_03876	SOG
b3527	KPN_03877	SOG
b3528	KPN_03878	SOG
b3529	KPN_03879	SOG
b3531	KPN_03881	SOG
b3532	KPN_03882	SOG
b3533	KPN_03883	SOG
b3536	KPN_03886	SOG
b3538	KPN_03888	SOG
b3540	KPN_03895	SOG
b3541	KPN_03896	SOG
b3542	KPN_03897	SOG
b3543	KPN_03898	SOG
b3544	KPN_03899	SOG
b3546	KPN_03904	SOG
b3549	KPN_03907	SOG
b3550	KPN_03908	SOG
b3551	KPN_03909	SOG
b3552	KPN_03910	SOG
b3553	KPN_03915	SOG
b3554	KPN_03916	SOG
b3559	KPN_03922	SOG
b3560	KPN_03923	SOG
b3561	KPN_03925	SOG
b3565	KPN_03929	SOG
b3569	KPN_03933	SOG
b3570	KPN_03934	SOG
b3571	KPN_03935	SOG
b3572	KPN_03936	SOG
b3590	KPN_03938	SOG
b3592	KPN_03940	SOG
b3599	KPN_03942	SOG
b3600	KPN_03943	SOG
b3601	KPN_03944	SOG
b3603	KPN_03947	SOG
b3604	KPN_03948	SOG
b3605	KPN_03949	SOG
b3606	KPN_03950	SOG
b3607	KPN_03951	SOG
b3608	KPN_03952	SOG
b3609	KPN_03953	SOG
b3611	KPN_03955	SOG
b3612	KPN_03956	SOG

b3005	STM3158	SOG
b3006	STM3159	SOG
b3009	STM3162	SOG
b3010	STM3163	SOG
b3011	STM3164	SOG
b3012	STM3165	SOG
b3017	STM3172	SOG
b3018	STM3173	SOG
b3019	STM3174	SOG
b3025	STM3177	SOG
b3026	STM3178	SOG
b3028	STM3179	SOG
b3030	STM3181	SOG
b3031	STM3182	SOG
b3032	STM3183	SOG
b3033	STM3184	SOG
b3034	STM3185	SOG
b3035	STM3186	SOG
b3037	STM3187	SOG
b3038	STM3188	SOG
b3039	STM3189	SOG
b3040	STM3190	SOG
b3041	STM3195	SOG
b3052	STM3200	SOG
b3053	STM3201	SOG
b3054	STM3202	SOG
b3055	STM3203	SOG
b3056	STM3204	SOG
b3057	STM3205	SOG
b3059	STM3207	SOG
b3061	STM3355	COG
b3062	STM3354	COG
b3064	STM3208	SOG
b3066	STM3210	SOG
b3067	STM3211.S	SOG
b3068	STM3212	SOG
b3070	STM3214	SOG
b3073	STM3218	SOG
b3081	STM3219	SOG
b3084	STM3220	SOG
b3085	STM3221	SOG
b3087	STM3223	SOG
b3088	STM3224	SOG
b3089	STM3225	SOG
b3092	STM3137	COG
b3095	STM3226	SOG

STM3555	KPN_03811	SOG
STM3556	KPN_03812	SOG
STM3557	KPN_03813	SOG
STM3560	KPN_03816	SOG
STM3561	KPN_03817	SOG
STM3562	KPN_03818	SOG
STM3563	KPN_03819	SOG
STM3564	KPN_03820	SOG
STM3567	KPN_03822	SOG
STM3568	KPN_03827	SOG
STM3569	KPN_03828	SOG
STM3570	KPN_03829	SOG
STM3571	KPN_03830	SOG
STM3572	KPN_03831	SOG
STM3575	KPN_03834	SOG
STM3579	KPN_03837	SOG
STM3580	KPN_03838	SOG
STM3581.S	KPN_03839	SOG
STM2298	KPN_03846	COG
STM3588	KPN_03857	SOG
STM3589	KPN_03858	SOG
STM3591	KPN_03860	SOG
STM3592	KPN_03861	SOG
STM3593	KPN_03862	SOG
STM3594	KPN_03863	SOG
STM3596	KPN_03865	SOG
STM3597	KPN_03866	SOG
STM3603	KPN_03869	SOG
STM3607	KPN_03872	SOG
STM3608	KPN_03873	SOG
STM3609	KPN_03874	SOG
STM3610	KPN_03875	SOG
STM3612	KPN_03876	SOG
STM3613	KPN_03877	SOG
STM3614	KPN_03878	SOG
STM3615	KPN_03879	SOG
STM3617	KPN_03881	SOG
STM3618	KPN_03882	SOG
STM3619	KPN_03883	SOG
STM3622	KPN_03886	SOG
STM3624	KPN_03888	SOG
STM3626	KPN_03895	SOG
STM3627	KPN_03896	SOG
STM3628	KPN_03897	SOG
STM3629	KPN_03898	SOG
STM3630	KPN_03899	SOG

b3613	KPN_03958	SOG
b3614	KPN_03959	SOG
b3616	KPN_03961	SOG
b3617	KPN_03962	SOG
b3619	KPN_03963	SOG
b3620	KPN_03964	SOG
b3633	KPN_03972	SOG
b3634	KPN_03974	SOG
b3635	KPN_03975	SOG
b3638	KPN_03978	SOG
b3639	KPN_03979	SOG
b3640	KPN_03980	SOG
b3641	KPN_03981	SOG
b3642	KPN_03983	SOG
b3643	KPN_03984	SOG
b3644	KPN_03990	SOG
b3646	KPN_03994	SOG
b3648	KPN_03996	SOG
b3650	KPN_03998	SOG
b3651	KPN_03999	SOG
b3652	KPN_04000	SOG
b3925	KPN_04001	COG
b3924	KPN_04002	COG
b3921	KPN_04003	COG
b3920	KPN_04004	COG
b3919	KPN_04005	COG
b3918	KPN_04007	COG
b3917	KPN_04008	COG
b3927	KPN_04010	COG
b3926	KPN_04011	COG
b3653	KPN_04012	SOG
b3654	KPN_04013	SOG
b3656	KPN_04018	SOG
b3657	KPN_04019	SOG
b3662	KPN_04065	SOG
b3663	KPN_04068	SOG
b3666	KPN_04069	SOG
b3667	KPN_04070	SOG
b3668	KPN_04071	SOG
b3669	KPN_04072	SOG
b3671	KPN_04074	SOG
b3673	KPN_04078	SOG
b3685	KPN_04088	SOG
b3686	KPN_04090	SOG
b3691	KPN_04094	SOG
b4478	KPN_04095	SOG

b3102	STM3233	SOG
b3105	STM3235	SOG
b3106	STM3236	SOG
b3114	STM3241	SOG
b3115	STM3242	SOG
b3116	STM3243	SOG
b3117	STM3244	SOG
b3118	STM3245	SOG
b3124	STM3247	SOG
b3125	STM3248	SOG
b3126	STM3249	SOG
b3128	STM3250	SOG
b3146	STM3263	SOG
b3147	STM3264	SOG
b3149	STM3266	SOG
b3150	STM3267	SOG
b3152	STM3268	SOG
b3153	STM3269	SOG
b3154	STM3270	SOG
b3156	STM3272	SOG
b3157	STM3273	SOG
b3159	STM3275.S	SOG
b3160	STM3276	SOG
b3161	STM3279	SOG
b3162	STM3280.S	SOG
b3163	STM3281	SOG
b3164	STM3282	SOG
b3166	STM3284	SOG
b3168	STM3286	SOG
b3169	STM3287	SOG
b3170	STM3288	SOG
b3172	STM3290.S	SOG
b3176	STM3294	SOG
b3177	STM3295	SOG
b3178	STM3296	SOG
b3179	STM3297	SOG
b3181	STM3299	SOG
b3182	STM3300	SOG
b3183	STM3301	SOG
b3187	STM3305	SOG
b3189	STM3307	SOG
b3192	STM3310	SOG
b3193	STM3311	SOG
b3194	STM3312	SOG
b3195	STM3313	SOG
b3196	STM3314	SOG

STM3635	KPN_03904	SOG
STM3642	KPN_03907	SOG
STM3643	KPN_03908	SOG
STM3644	KPN_03909	SOG
STM3645	KPN_03910	SOG
STM3646	KPN_03915	SOG
STM3647	KPN_03916	SOG
STM3655	KPN_03922	SOG
STM3656	KPN_03923	SOG
STM3661	KPN_03929	SOG
STM3662	KPN_03933	SOG
STM3663	KPN_03934	SOG
STM3664	KPN_03935	SOG
STM3665	KPN_03936	SOG
STM3682	KPN_03938	SOG
STM3683	KPN_03939	SOG
STM3685	KPN_03942	SOG
STM3686	KPN_03943	SOG
STM3687	KPN_03944	SOG
STM3692	KPN_03947	SOG
STM3693	KPN_03948	SOG
STM3694	KPN_03949	SOG
STM3695	KPN_03950	SOG
STM3699	KPN_03951	SOG
STM3700	KPN_03952	SOG
STM3701	KPN_03953	SOG
STM3703	KPN_03955	SOG
STM3704	KPN_03956	SOG
STM3705	KPN_03958	SOG
STM3706	KPN_03959	SOG
STM3708	KPN_03961	SOG
STM3709	KPN_03962	SOG
STM3710	KPN_03963	SOG
STM3711	KPN_03964	SOG
STM3724	KPN_03972	SOG
STM3725	KPN_03974	SOG
STM3726	KPN_03975	SOG
STM3730	KPN_03979	SOG
STM3731	KPN_03980	SOG
STM3732	KPN_03981	SOG
STM3733	KPN_03983	SOG
STM3734	KPN_03984	SOG
STM3735	KPN_03990	SOG
STM3740	KPN_03996	SOG
STM3742	KPN_03998	SOG
STM3743	KPN_03999	SOG

b3693	KPN_04097	SOG
b4479	KPN_04098	SOG
b3697	KPN_04101	SOG
b3699	KPN_04102	SOG
b3700	KPN_04103	SOG
b3701	KPN_04104	SOG
b3702	KPN_04105	SOG
b3705	KPN_04109	SOG
b3706	KPN_04110	SOG
b3713	KPN_04123	SOG
b3714	KPN_04124	SOG
b3724	KPN_04129	SOG
b3725	KPN_04130	SOG
b3726	KPN_04131	SOG
b3727	KPN_04132	SOG
b3728	KPN_04133	SOG
b3729	KPN_04134	SOG
b3730	KPN_04135	SOG
b3732	KPN_04137	SOG
b3733	KPN_04138	SOG
b3734	KPN_04139	SOG
b3735	KPN_04140	SOG
b3736	KPN_04141	SOG
b3738	KPN_04143	SOG
b3741	KPN_04146	SOG
b3742	KPN_04147	SOG
b3743	KPN_04148	SOG
b3744	KPN_04149	SOG
b3745	KPN_04150	SOG
b3746	KPN_04151	SOG
b3747	KPN_04152	SOG
b3749	KPN_04154	SOG
b3750	KPN_04155	SOG
b3751	KPN_04156	SOG
b3752	KPN_04157	SOG
b3753	KPN_04158	SOG
b3754	KPN_04159	SOG
b3755	KPN_04160	SOG
b3857	KPN_04167	SOG
b3859	KPN_04169	SOG
b3860	KPN_04170	SOG
b3862	KPN_04171	SOG
b3863	KPN_04172	SOG
b3865	KPN_04173	SOG
b3866	KPN_04174	SOG
b3867	KPN_04175	SOG

b3197	STM3315	SOG
b3198	STM3316	SOG
b3199	STM3317	SOG
b3200	STM3318	SOG
b3201	STM3319	SOG
b3202	STM3320	SOG
b3204	STM3322	SOG
b3205	STM3323	SOG
b3208	STM3326	SOG
b3210	STM3328	SOG
b3211	STM3329	SOG
b3213	STM3331	SOG
b3223	STM3337	SOG
b3228	STM3341	SOG
b3229	STM3342	SOG
b3231	STM3345	SOG
b3232	STM3346	SOG
b3234	STM3348	SOG
b3235	STM3349	SOG
b3236	STM3359	SOG
b3237	STM3360	SOG
b3240	STM3364	SOG
b3241	STM3365	SOG
b3243	STM3367	SOG
b3244	STM3368	SOG
b3247	STM3370	SOG
b3248	STM3371	SOG
b3249	STM3372	SOG
b3250	STM3373	SOG
b3251	STM3374	SOG
b3252	STM3375	SOG
b3253	STM3376	SOG
b3255	STM3379	SOG
b3256	STM3380	SOG
b3258	STM3382	SOG
b3259	STM3383	SOG
b3260	STM3384	SOG
b3266	STM3391	SOG
b3279	STM3399	SOG
b3282	STM3402	SOG
b3283	STM3403	SOG
b3284	STM3404	SOG
b3287	STM3406	SOG
b3288	STM3407	SOG
b3289	STM3408	SOG
b3290	STM3409	SOG

STM3744	KPN_04000	SOG
STM4085	KPN_04001	COG
STM4084	KPN_04002	COG
STM4083	KPN_04003	COG
STM4082	KPN_04004	COG
STM4081	KPN_04005	COG
STM4064	KPN_04007	COG
STM4063	KPN_04008	COG
STM4087	KPN_04010	COG
STM4086	KPN_04011	COG
STM3746	KPN_04012	SOG
STM3747	KPN_04013	SOG
STM3749	KPN_04018	SOG
STM3750	KPN_04019	SOG
STM3765	KPN_04045	SOG
STM3776	KPN_04065	SOG
STM3786	KPN_04068	SOG
STM3787	KPN_04069	SOG
STM3788	KPN_04070	SOG
STM3789	KPN_04071	SOG
STM3790	KPN_04072	SOG
STM3796	KPN_04074	SOG
STM3798	KPN_04078	SOG
STM3800	KPN_04079	SOG
STM3801	KPN_04080	SOG
STM3807	KPN_04088	SOG
STM3808.S	KPN_04090	SOG
STM3827	KPN_04094	SOG
STM3828	KPN_04095	SOG
STM3829	KPN_04097	SOG
STM3830	KPN_04098	SOG
STM3831	KPN_04101	SOG
STM3835	KPN_04102	SOG
STM3836	KPN_04103	SOG
STM3837	KPN_04104	SOG
STM3838	KPN_04105	SOG
STM3842	KPN_04109	SOG
STM3843	KPN_04110	SOG
STM3850	KPN_04123	SOG
STM3851.S	KPN_04124	SOG
STM3852	KPN_04125	SOG
STM3853	KPN_04129	SOG
STM3854	KPN_04130	SOG
STM3855	KPN_04131	SOG
STM3856	KPN_04132	SOG
STM3857	KPN_04133	SOG

b3868	KPN_04176	SOG
b3869	KPN_04177	SOG
b3870	KPN_04178	SOG
b3871	KPN_04179	SOG
b3885	KPN_04180	SOG
b3886	KPN_04181	SOG
b3887	KPN_04182	SOG
b3888	KPN_04183	SOG
b3891	KPN_04187	SOG
b3892	KPN_04188	SOG
b3893	KPN_04189	SOG
b3894	KPN_04190	SOG
b3895	KPN_04191	SOG
b3902	KPN_04211	SOG
b3903	KPN_04212	SOG
b3904	KPN_04213	SOG
b3905	KPN_04214	SOG
b3906	KPN_04215	SOG
b3907	KPN_04216	SOG
b3908	KPN_04217	SOG
b3911	KPN_04219	SOG
b3912	KPN_04220	SOG
b4484	KPN_04221	SOG
b3915	KPN_04222	SOG
b3916	KPN_04223	SOG
b3929	KPN_04224	SOG
b3931	KPN_04226	SOG
b3932	KPN_04227	SOG
b3933	KPN_04228	SOG
b3934	KPN_04229	SOG
b3935	KPN_04230	SOG
b3939	KPN_04233	SOG
b3940	KPN_04234	SOG
b3941	KPN_04239	SOG
b3945	KPN_04241	SOG
b3946	KPN_04242	SOG
b3956	KPN_04245	SOG
b3957	KPN_04246	SOG
b3958	KPN_04247	SOG
b3959	KPN_04248	SOG
b3960	KPN_04249	SOG
b3961	KPN_04250	SOG
b3962	KPN_04251	SOG
b3963	KPN_04252	SOG
b3965	KPN_04254	SOG
b3966	KPN_04255	SOG

b3292	STM3412	SOG
b3295	STM3415	SOG
b3296	STM3416	SOG
b3300	STM3420	SOG
b3301	STM3421	SOG
b3303	STM3423	SOG
b3305	STM3425	SOG
b3308	STM3428	SOG
b3314	STM3434	SOG
b3317	STM3437	SOG
b3319	STM3439	SOG
b3320	STM3440	SOG
b3339	STM4146	COG
b3340	STM3446	SOG
b3341	STM3447	SOG
b3346	STM3452	SOG
b3347	STM3453	SOG
b3349	STM3455	SOG
b3350	STM3457	SOG
b3351	STM3458	SOG
b3352	STM3459	SOG
b3353	STM3462	SOG
b3355	STM3464	SOG
b3357	STM3466	SOG
b3358	STM3467	SOG
b3360	STM3469	SOG
b3361	STM3470	SOG
b3363	STM3472	SOG
b3364	STM3473	SOG
b3368	STM3477	SOG
b3384	STM3481	SOG
b3385	STM3482	SOG
b3386	STM3483	SOG
b3387	STM3484	SOG
b3388	STM3485	SOG
b3389	STM3486	SOG
b3390	STM3487	SOG
b3391	STM3488	SOG
b3396	STM3493	SOG
b3397	STM3494.S	SOG
b3398	STM3495	SOG
b3399	STM3496	SOG
b3401	STM3498	SOG
b3403	STM3500	SOG
b3404	STM3501	SOG
b3405	STM3502	SOG

STM3861	KPN_04134	SOG
STM3862	KPN_04135	SOG
STM3865	KPN_04137	SOG
STM3866	KPN_04138	SOG
STM3867	KPN_04139	SOG
STM3868	KPN_04140	SOG
STM3869	KPN_04141	SOG
STM3871	KPN_04143	SOG
STM3873.S	KPN_04145	SOG
STM3874	KPN_04146	SOG
STM3875	KPN_04147	SOG
STM3876	KPN_04148	SOG
STM3877	KPN_04149	SOG
STM3878.S	KPN_04150	SOG
STM3879	KPN_04151	SOG
STM3880	KPN_04152	SOG
STM3882	KPN_04154	SOG
STM3883	KPN_04155	SOG
STM3884	KPN_04156	SOG
STM3885	KPN_04157	SOG
STM3886	KPN_04158	SOG
STM3888	KPN_04160	SOG
STM3996	KPN_04169	SOG
STM3997	KPN_04170	SOG
STM3999	KPN_04172	SOG
STM4001	KPN_04173	SOG
STM4003	KPN_04174	SOG
STM4004	KPN_04175	SOG
STM4005	KPN_04176	SOG
STM4006	KPN_04177	SOG
STM4007	KPN_04178	SOG
STM4009	KPN_04179	SOG
STM4026	KPN_04180	SOG
STM4027	KPN_04181	SOG
STM4028	KPN_04182	SOG
STM4029	KPN_04183	SOG
STM4034	KPN_04187	SOG
STM4035	KPN_04188	SOG
STM4036	KPN_04189	SOG
STM4037	KPN_04190	SOG
STM4038	KPN_04191	SOG
STM4045	KPN_04211	SOG
STM4046	KPN_04212	SOG
STM4047	KPN_04213	SOG
STM4048	KPN_04214	SOG
STM4049	KPN_04215	SOG

b3967	KPN_04256	SOG
b3770	KPN_04269	SOG
b3771	KPN_04270	SOG
b3772	KPN_04271	SOG
b3773	KPN_04272	SOG
b3774	KPN_04273	SOG
b3778	KPN_04276	SOG
b3779	KPN_04280	SOG
b3780	KPN_04281	SOG
b3783	KPN_04283	SOG
b3784	KPN_04284	SOG
b3786	KPN_04286	SOG
b3787	KPN_04287	SOG
b3788	KPN_04288	SOG
b3790	KPN_04290	SOG
b3791	KPN_04291	SOG
b3792	KPN_04292	SOG
b4481	KPN_04293	SOG
b3793	KPN_04294	SOG
b3794	KPN_04295	SOG
b3795	KPN_04296	SOG
b3802	KPN_04301	SOG
b3803	KPN_04302	SOG
b3804	KPN_04303	SOG
b3805	KPN_04304	SOG
b3806	KPN_04305	SOG
b3809	KPN_04308	SOG
b3810	KPN_04309	SOG
b3812	KPN_04311	SOG
b3813	KPN_04312	SOG
b3816	KPN_04313	SOG
b3819	KPN_04314	SOG
b3820	KPN_04315	SOG
b3821	KPN_04316	SOG
b3822	KPN_04317	SOG
b3823	KPN_04318	SOG
b3824	KPN_04320	SOG
b3825	KPN_04321	SOG
b3826	KPN_04322	SOG
b3827	KPN_04323	SOG
b3829	KPN_04325	SOG
b3830	KPN_04326	SOG
b3831	KPN_04327	SOG
b3832	KPN_04328	SOG
b3833	KPN_04329	SOG
b3834	KPN_04330	SOG

b3406	STM3503	SOG
b3407	STM3504	SOG
b3409	STM3506	SOG
b3412	STM3509	SOG
b3413	STM3510	SOG
b3414	STM3511	SOG
b3416	STM3513	SOG
b3417	STM3514	SOG
b3418	STM3515	SOG
b3423	STM3523	SOG
b3424	STM3524	SOG
b3428	STM3534	SOG
b3429	STM3535	SOG
b3430	STM3536	SOG
b3431	STM3537	SOG
b3432	STM3538	SOG
b3433	STM3539	SOG
b3437	STM3542	SOG
b3438	STM3543	SOG
b3439	STM3544	SOG
b3440	STM3545	SOG
b3441	STM3546	SOG
b3447	STM3551	SOG
b3449	STM3553	SOG
b3450	STM3554	SOG
b3451	STM3555	SOG
b3452	STM3556	SOG
b3453	STM3557	SOG
b3454	STM3560	SOG
b3455	STM3561	SOG
b3456	STM3562	SOG
b3457	STM3563	SOG
b3458	STM3564	SOG
b3460	STM3567	SOG
b3461	STM3568	SOG
b3462	STM3569	SOG
b3463	STM3570	SOG
b3464	STM3571	SOG
b3465	STM3572	SOG
b3468	STM3575	SOG
b3469	STM3576	SOG
b3471	STM3579	SOG
b3472	STM3580	SOG
b3473	STM3581.S	SOG
b3474	STM3582	SOG
b3485	STM3585	SOG

STM4050	KPN_04216	SOG
STM4055	KPN_04217	SOG
STM4058	KPN_04219	SOG
STM4059	KPN_04220	SOG
STM4060	KPN_04221	SOG
STM4061	KPN_04222	SOG
STM4062	KPN_04223	SOG
STM4089	KPN_04224	SOG
STM4090	KPN_04225	SOG
STM4091	KPN_04226	SOG
STM4092	KPN_04227	SOG
STM4093	KPN_04228	SOG
STM4094	KPN_04229	SOG
STM4095	KPN_04230	SOG
STM4100	KPN_04233	SOG
STM4101	KPN_04234	SOG
STM4105	KPN_04239	SOG
STM4108	KPN_04241	SOG
STM4109	KPN_04242	SOG
STM4117	KPN_04243	SOG
STM4119	KPN_04245	SOG
STM4120	KPN_04246	SOG
STM4121	KPN_04247	SOG
STM4122	KPN_04248	SOG
STM4123	KPN_04249	SOG
STM4125	KPN_04250	SOG
STM4126	KPN_04251	SOG
STM4127	KPN_04252	SOG
STM4129	KPN_04254	SOG
STM4130	KPN_04255	SOG
STM4131	KPN_04256	SOG
STM3903	KPN_04269	SOG
STM3904	KPN_04270	SOG
STM3905	KPN_04271	SOG
STM3908	KPN_04272	SOG
STM3909	KPN_04273	SOG
STM3912	KPN_04276	SOG
STM3913	KPN_04280	SOG
STM3914	KPN_04281	SOG
STM3917	KPN_04283	SOG
STM3919	KPN_04285	SOG
STM3920	KPN_04286	SOG
STM3921	KPN_04287	SOG
STM3922	KPN_04288	SOG
STM3925	KPN_04291	SOG
STM3927	KPN_04293	SOG

b3835	KPN_04331	SOG
b3838	KPN_04333	SOG
b3839	KPN_04334	SOG
b3842	KPN_04336	SOG
b3843	KPN_04337	SOG
b3844	KPN_04338	SOG
b3845	KPN_04339	SOG
b3846	KPN_04340	SOG
b3847	KPN_04341	SOG
b3848	KPN_04342	SOG
b3849	KPN_04343	SOG
b3850	KPN_04344	SOG
b3974	KPN_04352	SOG
b3339	KPN_04357	COG
b3982	KPN_04359	SOG
b3983	KPN_04360	SOG
b3984	KPN_04361	SOG
b3985	KPN_04363	SOG
b3987	KPN_04365	SOG
b3988	KPN_04366	SOG
b4123	KPN_04367	COG
b3990	KPN_04371	SOG
b3991	KPN_04372	SOG
b3993	KPN_04375	SOG
b3994	KPN_04376	SOG
b3995	KPN_04377	SOG
b3996	KPN_04378	SOG
b3997	KPN_04379	SOG
b3998	KPN_04380	SOG
b3999	KPN_04381	SOG
b4001	KPN_04383	SOG
b4003	KPN_04385	SOG
b4004	KPN_04386	SOG
b4005	KPN_04387	SOG
b4006	KPN_04388	SOG
b4012	KPN_04393	SOG
b4013	KPN_04394	SOG
b4014	KPN_04395	SOG
b4015	KPN_04396	SOG
b4016	KPN_04397	SOG
b4019	KPN_04399	SOG
b4020	KPN_04400	SOG
b4022	KPN_04409	SOG
b4024	KPN_04412	SOG
b4025	KPN_04413	SOG
b4032	KPN_04421	SOG

b3486	STM3586.S	SOG
b3487	STM3587	SOG
b3492	STM3588	SOG
b3493	STM3589	SOG
b3495	STM3591	SOG
b3496	STM3592	SOG
b3497	STM3593	SOG
b3498	STM3594	SOG
b3499	STM3596	SOG
b3500	STM3597	SOG
b3519	STM3603	SOG
b3521	STM3607	SOG
b3522	STM3608	SOG
b3523	STM3609	SOG
b3524	STM3610	SOG
b3526	STM3612	SOG
b3527	STM3613	SOG
b3528	STM3614	SOG
b3529	STM3615	SOG
b3530	STM3616	SOG
b3531	STM3617	SOG
b3532	STM3618	SOG
b3533	STM3619	SOG
b3536	STM3622	SOG
b3538	STM3624	SOG
b3540	STM3626	SOG
b3541	STM3627	SOG
b3542	STM3628	SOG
b3543	STM3629	SOG
b3544	STM3630	SOG
b3546	STM3635	SOG
b3550	STM3643	SOG
b3551	STM3644	SOG
b3552	STM3645	SOG
b3553	STM3646	SOG
b3554	STM3647	SOG
b3559	STM3655	SOG
b3560	STM3656	SOG
b3561	STM3658	SOG
b3564	STM3660	SOG
b3565	STM3661	SOG
b3569	STM3662	SOG
b3570	STM3663	SOG
b3571	STM3664	SOG
b3572	STM3665	SOG
b3588	STM3680	SOG

STM3928	KPN_04294	SOG
STM3929	KPN_04295	SOG
STM3930	KPN_04296	SOG
STM3935	KPN_04301	SOG
STM3936	KPN_04302	SOG
STM3937	KPN_04303	SOG
STM3938	KPN_04304	SOG
STM3939	KPN_04305	SOG
STM3947	KPN_04308	SOG
STM3948	KPN_04309	SOG
STM3950	KPN_04311	SOG
STM3951	KPN_04312	SOG
STM3952	KPN_04313	SOG
STM3955	KPN_04314	SOG
STM3956	KPN_04315	SOG
STM3957	KPN_04316	SOG
STM3958	KPN_04317	SOG
STM3959	KPN_04318	SOG
STM3960	KPN_04320	SOG
STM3962	KPN_04322	SOG
STM3963	KPN_04323	SOG
STM3965	KPN_04325	SOG
STM3967	KPN_04326	SOG
STM3968	KPN_04327	SOG
STM3970	KPN_04329	SOG
STM3971	KPN_04330	SOG
STM3972	KPN_04331	SOG
STM3974	KPN_04333	SOG
STM3975	KPN_04334	SOG
STM3977	KPN_04336	SOG
STM3978	KPN_04337	SOG
STM3979	KPN_04338	SOG
STM3982	KPN_04339	SOG
STM3983	KPN_04340	SOG
STM3984	KPN_04341	SOG
STM3985	KPN_04342	SOG
STM3986	KPN_04343	SOG
STM3987	KPN_04344	SOG
STM4139	KPN_04352	SOG
STM4146	KPN_04357	SOG
STM4148	KPN_04359	SOG
STM4149	KPN_04360	SOG
STM4150	KPN_04361	SOG
STM4151	KPN_04363	SOG
STM4153	KPN_04365	SOG
STM4154	KPN_04366	SOG

b4033	KPN_04422	SOG
b4034	KPN_04423	SOG
b4035	KPN_04424	SOG
b4036	KPN_04425	SOG
b4037	KPN_04426	SOG
b4039	KPN_04427	SOG
b4040	KPN_04428	SOG
b4041	KPN_04429	SOG
b4043	KPN_04431	SOG
b4044	KPN_04432	SOG
b4046	KPN_04434	SOG
b4049	KPN_04436	SOG
b4051	KPN_04438	SOG
b4052	KPN_04439	SOG
b4053	KPN_04440	SOG
b4054	KPN_04441	SOG
b4055	KPN_04442	SOG
b4058	KPN_04445	SOG
b4059	KPN_04446	SOG
b4063	KPN_04463	SOG
b4064	KPN_04465	SOG
b4065	KPN_04466	SOG
b4067	KPN_04476	SOG
b4069	KPN_04478	SOG
b4077	KPN_04480	SOG
b4079	KPN_04482	SOG
b4111	KPN_04503	SOG
b4118	KPN_04504	SOG
b4119	KPN_04505	SOG
b1000	KPN_04509	SOG
b4136	KPN_04526	SOG
b4139	KPN_04529	SOG
b4140	KPN_04530	SOG
b4141	KPN_04531	SOG
b4143	KPN_04533	SOG
b4146	KPN_04544	SOG
b4147	KPN_04545	SOG
b4149	KPN_04549	SOG
b4153	KPN_04552	SOG
b4154	KPN_04553	SOG
b4155	KPN_04554	SOG
b4159	KPN_04556	SOG
b4160	KPN_04557	SOG
b4161	KPN_04558	SOG
b4162	KPN_04559	SOG
b4166	KPN_04564	SOG

b3590	STM3682	SOG
b3591	STM3683	SOG
b3592	STM3684	SOG
b3599	STM3685	SOG
b3600	STM3686	SOG
b3601	STM3687	SOG
b3603	STM3692	SOG
b3604	STM3693	SOG
b3605	STM3694	SOG
b3606	STM3695	SOG
b3607	STM3699	SOG
b3608	STM3700	SOG
b3609	STM3701	SOG
b3611	STM3703	SOG
b3612	STM3704	SOG
b3613	STM3705	SOG
b3614	STM3706	SOG
b3616	STM3708	SOG
b3617	STM3709	SOG
b3619	STM3710	SOG
b3620	STM3711	SOG
b3621	STM3712	SOG
b3633	STM3724	SOG
b3634	STM3725	SOG
b3635	STM3726	SOG
b3638	STM3729	SOG
b3639	STM3730	SOG
b3640	STM3731	SOG
b3641	STM3732	SOG
b3642	STM3733	SOG
b3643	STM3734	SOG
b3644	STM3735	SOG
b3646	STM3738	SOG
b3647	STM3739	SOG
b3648	STM3740	SOG
b3650	STM3742	SOG
b3651	STM3743	SOG
b3652	STM3744	SOG
b3653	STM3746	SOG
b3654	STM3747	SOG
b3655	STM3748	SOG
b3656	STM3749	SOG
b3657	STM3750	SOG
b3660	STM3765	SOG
b3662	STM3776	SOG
b3663	STM3786	SOG

STM4301	KPN_04367	COG
STM4159	KPN_04371	SOG
STM4160	KPN_04372	SOG
STM4162	KPN_04374	SOG
STM4163	KPN_04375	SOG
STM4164	KPN_04376	SOG
STM4165	KPN_04377	SOG
STM4166	KPN_04378	SOG
STM4167	KPN_04379	SOG
STM4168	KPN_04380	SOG
STM4169	KPN_04381	SOG
STM4171	KPN_04383	SOG
STM4172	KPN_04384	SOG
STM4174	KPN_04386	SOG
STM4175	KPN_04387	SOG
STM4176	KPN_04388	SOG
STM4181	KPN_04393	SOG
STM4182	KPN_04394	SOG
STM4183	KPN_04395	SOG
STM4184	KPN_04396	SOG
STM4185	KPN_04397	SOG
STM4188.S	KPN_04399	SOG
STM4189	KPN_04400	SOG
STM4193	KPN_04409	SOG
STM4220	KPN_04412	SOG
STM4221	KPN_04413	SOG
STM4227	KPN_04421	SOG
STM4228	KPN_04422	SOG
STM4229	KPN_04423	SOG
STM4230	KPN_04424	SOG
STM4231	KPN_04425	SOG
STM4232	KPN_04426	SOG
STM4233	KPN_04427	SOG
STM4235	KPN_04429	SOG
STM4237	KPN_04431	SOG
STM4238	KPN_04432	SOG
STM4241	KPN_04434	SOG
STM4243	KPN_04436	SOG
STM4245	KPN_04438	SOG
STM4246	KPN_04439	SOG
STM4247	KPN_04440	SOG
STM4248	KPN_04441	SOG
STM4254	KPN_04445	SOG
STM4256	KPN_04446	SOG
STM4266	KPN_04463	SOG
STM4268	KPN_04465	SOG

b4167	KPN_04565	SOG
b4168	KPN_04566	SOG
b4169	KPN_04567	SOG
b4170	KPN_04568	SOG
b4171	KPN_04569	SOG
b4173	KPN_04571	SOG
b4174	KPN_04572	SOG
b4175	KPN_04573	SOG
b4177	KPN_04576	SOG
b4179	KPN_04578	SOG
b4180	KPN_04579	SOG
b4187	KPN_04580	SOG
b4191	KPN_04584	SOG
b4192	KPN_04585	SOG
b4193	KPN_04586	SOG
b4195	KPN_04588	SOG
b4196	KPN_04589	SOG
b4197	KPN_04590	SOG
b4198	KPN_04591	SOG
b4203	KPN_04597	SOG
b4207	KPN_04600	SOG
b4208	KPN_04601	SOG
b4209	KPN_04602	SOG
b4210	KPN_04603	SOG
b4211	KPN_04605	SOG
b4213	KPN_04607	SOG
b4214	KPN_04608	SOG
b4216	KPN_04609	SOG
b4218	KPN_04614	SOG
b4219	KPN_04615	SOG
b4220	KPN_04616	SOG
b4221	KPN_04617	SOG
b4226	KPN_04621	SOG
b4232	KPN_04626	SOG
b4233	KPN_04627	SOG
b4234	KPN_04630	SOG
b4235	KPN_04631	SOG
b4237	KPN_04645	SOG
b4238	KPN_04646	SOG
b4239	KPN_04651	SOG
b4241	KPN_04653	SOG
b4242	KPN_04654	SOG
b4244	KPN_04656	SOG
b4254	KPN_04659	SOG
b4256	KPN_04662	SOG
b4259	KPN_04664	SOG

b3666	STM3787	SOG
b3667	STM3788	SOG
b3668	STM3789	SOG
b3669	STM3790	SOG
b3671	STM3796	SOG
b3673	STM3798	SOG
b3685	STM3807	SOG
b3686	STM3808.S	SOG
b3691	STM3827	SOG
b3693	STM3829	SOG
b3697	STM3831	SOG
b3699	STM3835	SOG
b3700	STM3836	SOG
b3701	STM3837	SOG
b3702	STM3838	SOG
b3705	STM3842	SOG
b3706	STM3843	SOG
b3710	STM3847	SOG
b3711	STM3848	SOG
b3712	STM3849	SOG
b3713	STM3850	SOG
b3714	STM3851.S	SOG
b3715	STM3852	SOG
b3724	STM3853	SOG
b3725	STM3854	SOG
b3726	STM3855	SOG
b3727	STM3856	SOG
b3728	STM3857	SOG
b3729	STM3861	SOG
b3730	STM3862	SOG
b3732	STM3865	SOG
b3733	STM3866	SOG
b3734	STM3867	SOG
b3735	STM3868	SOG
b3736	STM3869	SOG
b3738	STM3871	SOG
b3740	STM3873.S	SOG
b3741	STM3874	SOG
b3742	STM3875	SOG
b3743	STM3876	SOG
b3744	STM3877	SOG
b3745	STM3878.S	SOG
b3746	STM3879	SOG
b3747	STM3880	SOG
b3749	STM3882	SOG
b3750	STM3883	SOG

STM4269	KPN_04466	SOG
STM4273	KPN_04476	SOG
STM4275	KPN_04478	SOG
STM4283	KPN_04480	SOG
STM4285	KPN_04482	SOG
STM4290	KPN_04503	SOG
STM4297	KPN_04504	SOG
STM4298	KPN_04505	SOG
STM4299	KPN_04506	SOG
STM4323	KPN_04526	SOG
STM4326	KPN_04529	SOG
STM4327	KPN_04530	SOG
STM4330	KPN_04533	SOG
STM4333	KPN_04544	SOG
STM4334	KPN_04545	SOG
STM4339	KPN_04549	SOG
STM4342	KPN_04552	SOG
STM4343	KPN_04553	SOG
STM4344	KPN_04554	SOG
STM4347	KPN_04556	SOG
STM4348	KPN_04557	SOG
STM4349	KPN_04558	SOG
STM4350	KPN_04559	SOG
STM4355.S	KPN_04564	SOG
STM4356	KPN_04565	SOG
STM4357	KPN_04566	SOG
STM4358	KPN_04567	SOG
STM4359	KPN_04568	SOG
STM4360	KPN_04569	SOG
STM4362	KPN_04571	SOG
STM4363	KPN_04572	SOG
STM4364	KPN_04573	SOG
STM4366	KPN_04576	SOG
STM4368	KPN_04578	SOG
STM4369	KPN_04579	SOG
STM4381	KPN_04584	SOG
STM4382	KPN_04585	SOG
STM4383.S	KPN_04586	SOG
STM4385	KPN_04588	SOG
STM4386	KPN_04589	SOG
STM4387	KPN_04590	SOG
STM4388	KPN_04591	SOG
STM4394	KPN_04597	SOG
STM4397	KPN_04600	SOG
STM4398	KPN_04601	SOG
STM4399	KPN_04602	SOG

b4260	KPN_04665	SOG
b4261	KPN_04666	SOG
b4262	KPN_04667	SOG
b4269	KPN_04683	SOG
b0570	KPN_04741	COG
b3485	KPN_04749	SOG
b3486	KPN_04750	SOG
b3487	KPN_04751	SOG
b4336	KPN_04768	SOG
b4352	KPN_04774	SOG
b4354	KPN_04776	SOG
b4359	KPN_04809	SOG
b4360	KPN_04810	SOG
b4361	KPN_04811	SOG
b4362	KPN_04812	SOG
b4364	KPN_04814	SOG
b4367	KPN_04821	SOG
b4371	KPN_04827	SOG
b4373	KPN_04829	SOG
b4374	KPN_04830	SOG
b4375	KPN_04831	SOG
b4381	KPN_04837	SOG
b4382	KPN_04838	SOG
b4383	KPN_04839	SOG
b4384	KPN_04840	SOG
b4386	KPN_04841	SOG
b4387	KPN_04842	SOG
b4388	KPN_04843	SOG
b4389	KPN_04844	SOG
b4390	KPN_04845	SOG
b4391	KPN_04846	SOG
b4392	KPN_04847	SOG
b4394	KPN_04849	SOG
b4395	KPN_04850	SOG
b4396	KPN_04851	SOG
b4397	KPN_04852	SOG
b4398	KPN_04853	SOG
b4400	KPN_04855	SOG
b4401	KPN_04856	SOG

b3751	STM3884	SOG
b3752	STM3885	SOG
b3753	STM3886	SOG
b3754	STM3887	SOG
b3755	STM3888	SOG
b3770	STM3903	SOG
b3771	STM3904	SOG
b3772	STM3905	SOG
b3773	STM3908	SOG
b3774	STM3909	SOG
b3778	STM3912	SOG
b3779	STM3913	SOG
b3780	STM3914	SOG
b3783	STM3917	SOG
b3784	STM3918	SOG
b3785	STM3919	SOG
b3786	STM3920	SOG
b3787	STM3921	SOG
b3788	STM3922	SOG
b3790	STM3924	SOG
b3791	STM3925	SOG
b3792	STM3926	SOG
b3793	STM3928	SOG
b3794	STM3929	SOG
b3795	STM3930	SOG
b3802	STM3935	SOG
b3803	STM3936	SOG
b3804	STM3937	SOG
b3805	STM3938	SOG
b3806	STM3939	SOG
b3809	STM3947	SOG
b3810	STM3948	SOG
b3812	STM3950	SOG
b3813	STM3951	SOG
b3816	STM3952	SOG
b3819	STM3955	SOG
b3820	STM3956	SOG
b3821	STM3957	SOG
b3822	STM3958	SOG
b3823	STM3959	SOG
b3825	STM3961	SOG
b3826	STM3962	SOG
b3827	STM3963	SOG
b3829	STM3965	SOG
b3830	STM3967	SOG
b3831	STM3968	SOG

STM4401	KPN_04605	SOG
STM4403	KPN_04607	SOG
STM4404	KPN_04608	SOG
STM4405	KPN_04609	SOG
STM4407	KPN_04614	SOG
STM4408	KPN_04615	SOG
STM4409	KPN_04616	SOG
STM4410	KPN_04617	SOG
STM4414	KPN_04621	SOG
STM4415	KPN_04626	SOG
STM4416	KPN_04627	SOG
STM4437	KPN_04630	SOG
STM4438	KPN_04631	SOG
STM4451	KPN_04645	SOG
STM4452	KPN_04646	SOG
STM4453	KPN_04651	SOG
STM4455	KPN_04653	SOG
STM4456	KPN_04654	SOG
STM4459	KPN_04656	SOG
STM4468	KPN_04658	SOG
STM4469	KPN_04659	SOG
STM4476.S	KPN_04664	SOG
STM4477	KPN_04665	SOG
STM4479	KPN_04666	SOG
STM4480	KPN_04667	SOG
STM4486	KPN_04683	SOG
STM3585	KPN_04749	SOG
STM3586.S	KPN_04750	SOG
STM3587	KPN_04751	SOG
STM4517	KPN_04767	SOG
STM4516	KPN_04768	SOG
STM3508	KPN_04771	SOG
STM4530	KPN_04774	SOG
STM4532	KPN_04776	SOG
STM4541	KPN_04809	SOG
STM4542	KPN_04810	SOG
STM4543	KPN_04811	SOG
STM4545	KPN_04813	SOG
STM4546	KPN_04814	SOG
STM4550	KPN_04821	SOG
STM4556	KPN_04827	SOG
STM4558	KPN_04829	SOG
STM4559.S	KPN_04830	SOG
STM4560	KPN_04831	SOG
STM4563	KPN_04834	SOG
STM4567	KPN_04837	SOG

b3832	STM3969	SOG
b3833	STM3970	SOG
b3834	STM3971	SOG
b3835	STM3972	SOG
b3838	STM3974	SOG
b3839	STM3975	SOG
b3842	STM3977	SOG
b3843	STM3978	SOG
b3844	STM3979	SOG
b3845	STM3982	SOG
b3846	STM3983	SOG
b3847	STM3984	SOG
b3848	STM3985	SOG
b3849	STM3986	SOG
b3850	STM3987	SOG
b3857	STM3994	SOG
b3859	STM3996	SOG
b3860	STM3997	SOG
b3862	STM3998	SOG
b3863	STM3999	SOG
b3865	STM4001	SOG
b3866	STM4003	SOG
b3867	STM4004	SOG
b3868	STM4005	SOG
b3869	STM4006	SOG
b3870	STM4007	SOG
b3871	STM4009	SOG
b3885	STM4026	SOG
b3886	STM4027	SOG
b3887	STM4028	SOG
b3888	STM4029	SOG
b3891	STM4034	SOG
b3892	STM4035	SOG
b3893	STM4036	SOG
b3902	STM4045	SOG
b3903	STM4046	SOG
b3904	STM4047	SOG
b3905	STM4048	SOG
b3906	STM4049	SOG
b3907	STM4050	SOG
b3908	STM4055	SOG
b3911	STM4058	SOG
b3912	STM4059	SOG
b3915	STM4061	SOG
b3916	STM4062	SOG
b3917	STM4063	SOG

STM4568	KPN_04838	SOG
STM4569	KPN_04839	SOG
STM4570	KPN_04840	SOG
STM4576	KPN_04841	SOG
STM4577	KPN_04842	SOG
STM4578	KPN_04843	SOG
STM4579	KPN_04844	SOG
STM4580.S	KPN_04845	SOG
STM4581	KPN_04846	SOG
STM4582	KPN_04847	SOG
STM4584	KPN_04849	SOG
STM4585	KPN_04850	SOG
STM4586	KPN_04851	SOG
STM4587	KPN_04852	SOG
STM4588	KPN_04853	SOG
STM4589	KPN_04854	SOG
STM4590	KPN_04855	SOG
STM4598	KPN_04856	SOG

b3918	STM4064	SOG
b3919	STM4081	SOG
b3920	STM4082	SOG
b3921	STM4083	SOG
b3924	STM4084	SOG
b3925	STM4085	SOG
b3926	STM4086	SOG
b3927	STM4087	SOG
b3929	STM4089	SOG
b3930	STM4090	SOG
b3931	STM4091	SOG
b3932	STM4092	SOG
b3933	STM4093	SOG
b3934	STM4094	SOG
b3935	STM4095	SOG
b3939	STM4100	SOG
b3940	STM4101	SOG
b3941	STM4105	SOG
b3942	STM4106	SOG
b3945	STM4108	SOG
b3946	STM4109	SOG
b3954	STM4117	SOG
b3956	STM4119	SOG
b3957	STM4120	SOG
b3958	STM4121	SOG
b3959	STM4122	SOG
b3960	STM4123	SOG
b3961	STM4125	SOG
b3962	STM4126	SOG
b3963	STM4127	SOG
b3965	STM4129	SOG
b3966	STM4130	SOG
b3967	STM4131	SOG
b3972	STM4137	SOG
b3973	STM4138	SOG
b3974	STM4139	SOG
b3982	STM4148	SOG
b3983	STM4149	SOG
b3984	STM4150	SOG
b3985	STM4151	SOG
b3987	STM4153	SOG
b3988	STM4154	SOG
b3990	STM4159	SOG
b3991	STM4160	SOG
b3992	STM4162	SOG
b3993	STM4163	SOG

b3994	STM4164	SOG
b3995	STM4165	SOG
b3996	STM4166	SOG
b3997	STM4167	SOG
b3998	STM4168	SOG
b3999	STM4169	SOG
b4001	STM4171	SOG
b4002	STM4172	SOG
b4003	STM4173	SOG
b4004	STM4174	SOG
b4005	STM4175	SOG
b4006	STM4176	SOG
b4012	STM4181	SOG
b4013	STM4182	SOG
b4014	STM4183	SOG
b4015	STM4184	SOG
b4016	STM4185	SOG
b4019	STM4188.S	SOG
b4020	STM4189	SOG
b4022	STM4193	SOG
b4024	STM4220	SOG
b4025	STM4221	SOG
b4032	STM4227	SOG
b4033	STM4228	SOG
b4034	STM4229	SOG
b4035	STM4230	SOG
b4036	STM4231	SOG
b4037	STM4232	SOG
b4039	STM4233	SOG
b4040	STM4234	SOG
b4041	STM4235	SOG
b4043	STM4237	SOG
b4044	STM4238	SOG
b4046	STM4241	SOG
b4049	STM4243	SOG
b4051	STM4245	SOG
b4052	STM4246	SOG
b4053	STM4247	SOG
b4054	STM4248	SOG
b4055	STM4249	SOG
b4058	STM4254	SOG
b4059	STM4256	SOG
b4061	STM4264	SOG
b4063	STM4266	SOG
b4064	STM4268	SOG
b4065	STM4269	SOG

b4067	STM4273	SOG
b4069	STM4275	SOG
b4077	STM4283	SOG
b4078	STM4284	SOG
b4107	STM4288	SOG
b4111	STM4290	SOG
b4112	STM4291	SOG
b4113	STM4292	SOG
b4114	STM4293	SOG
b4118	STM4297	SOG
b4119	STM4298	SOG
b4123	STM4301	SOG
b4124	STM4303	SOG
b4125	STM4304	SOG
b4130	STM2560	COG
b4131	STM2559	COG
b4132	STM2558	COG
b4133	STM2557	COG
b4135	STM4322	SOG
b4136	STM4323	SOG
b4138	STM4325	SOG
b4139	STM4326	SOG
b4140	STM4327	SOG
b4143	STM4330	SOG
b4146	STM4333	SOG
b4147	STM4334	SOG
b4149	STM4339	SOG
b4153	STM4342	SOG
b4154	STM4343	SOG
b4155	STM4344	SOG
b4156	STM4345	SOG
b4159	STM4347	SOG
b4160	STM4348	SOG
b4161	STM4349	SOG
b4162	STM4350	SOG
b4166	STM4355.S	SOG
b4167	STM4356	SOG
b4168	STM4357	SOG
b4169	STM4358	SOG
b4170	STM4359	SOG
b4171	STM4360	SOG
b4173	STM4362	SOG
b4174	STM4363	SOG
b4175	STM4364	SOG
b4177	STM4366	SOG
b4179	STM4368	SOG

b4180	STM4369	SOG
b4187	STM4377	SOG
b4190	STM4380	SOG
b4191	STM4381	SOG
b4192	STM4382	SOG
b4193	STM4383.S	SOG
b4195	STM4385	SOG
b4196	STM4386	SOG
b4197	STM4387	SOG
b4198	STM4388	SOG
b4203	STM4394	SOG
b4206	STM4396	SOG
b4207	STM4397	SOG
b4208	STM4398	SOG
b4209	STM4399	SOG
b4210	STM4400	SOG
b4211	STM4401	SOG
b4213	STM4403	SOG
b4214	STM4404	SOG
b4216	STM4405	SOG
b4218	STM4407	SOG
b4219	STM4408	SOG
b4220	STM4409	SOG
b4221	STM4410	SOG
b4226	STM4414	SOG
b4232	STM4415	SOG
b4233	STM4416	SOG
b4234	STM4437	SOG
b4235	STM4438	SOG
b4237	STM4451	SOG
b4238	STM4452	SOG
b4239	STM4453	SOG
b4241	STM4455	SOG
b4242	STM4456	SOG
b4244	STM4459	SOG
b4249	STM1675	COG
b4252	STM4468	SOG
b4254	STM4469	SOG
b4259	STM4476.S	SOG
b4260	STM4477	SOG
b4261	STM4479	SOG
b4262	STM4480	SOG
b4269	STM4486	SOG
b4306	STM4500	SOG
b4322	STM3135	SOG
b4336	STM4516	SOG

b4337	STM4517	SOG
b4352	STM4530	SOG
b4354	STM4532	SOG
b4359	STM4541	SOG
b4360	STM4542	SOG
b4361	STM4543	SOG
b4362	STM4544	SOG
b4363	STM4545	SOG
b4364	STM4546	SOG
b4365	STM4547	SOG
b4367	STM4550	SOG
b4371	STM4556	SOG
b4373	STM4558	SOG
b4374	STM4559.S	SOG
b4375	STM4560	SOG
b4376	STM4561	SOG
b4377	STM4563	SOG
b4378	STM4564	SOG
b4381	STM4567	SOG
b4382	STM4568	SOG
b4383	STM4569	SOG
b4384	STM4570	SOG
b4386	STM4576	SOG
b4387	STM4577	SOG
b4388	STM4578	SOG
b4389	STM4579	SOG
b4390	STM4580.S	SOG
b4391	STM4581	SOG
b4392	STM4582	SOG
b4394	STM4584	SOG
b4395	STM4585	SOG
b4396	STM4586	SOG
b4397	STM4587	SOG
b4398	STM4588	SOG
b4399	STM4589	SOG
b4400	STM4590	SOG
b4401	STM4598	SOG
b4461	STM2679	SOG
b4469	STM3168	SOG
b4472	STM3369	SOG
b4473	STM3405	SOG
b4476	STM3541	SOG
b4478	STM3828	SOG
b4479	STM3830	SOG
b4480	STM3897	SOG
b4481	STM3927	SOG

b4483	STM3976.S	SOG
b4484	STM4060	SOG
