# Supplementary material for: Changes in transcriptional orientation are associated with increases in evolutionary rates of enterobacterial genes
Source: BMC Bioinformatics. 2011 Oct 5;12(Suppl 9):S19. doi: 10.1186/1471-2105-12-S9-S19 (PMC3283321; doi:10.1186/1471-2105-12-S9-S19)
Supplement: Additional file 2 — The numbers of ECO-KPN, ECO-STM, and STM-KPN orthologous gene pairs identified by reciprocal BLASTP and the OMA database [file 1471-2105-12-S9-S19-S2.pdf]

method	number	predicted only by one method
Blastp	2582	51
OMA	2607	76
overlapping	2531	

comparison	ortholgous gene pairs identified by both methods	COG	SOG	% COG
ECO-KPN	1768	125	1643	7.07%
ECO-STM	2048	248	1800	12.11%
STM-KPN	1796	104	1692	5.79%
