# Supplementary figures and images for: Changes in transcriptional orientation are associated with increases in evolutionary rates of enterobacterial genes
Source: BMC Bioinformatics. 2011 Oct 5;12(Suppl 9):S19. doi: 10.1186/1471-2105-12-S9-S19 (PMC3283321; doi:10.1186/1471-2105-12-S9-S19)

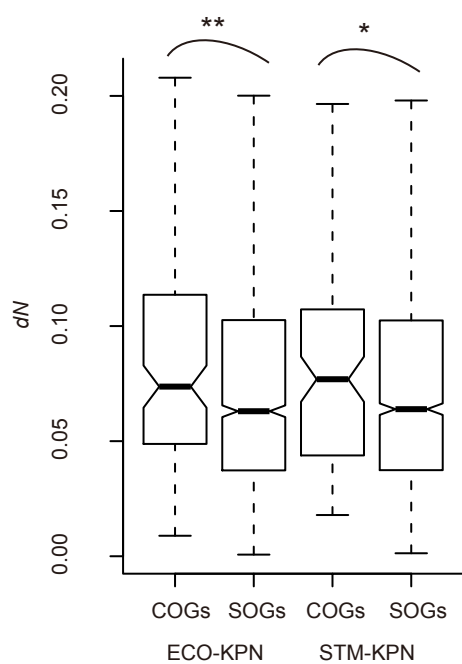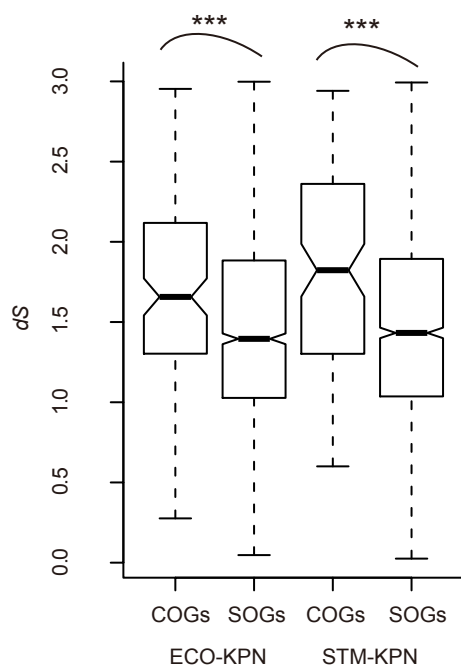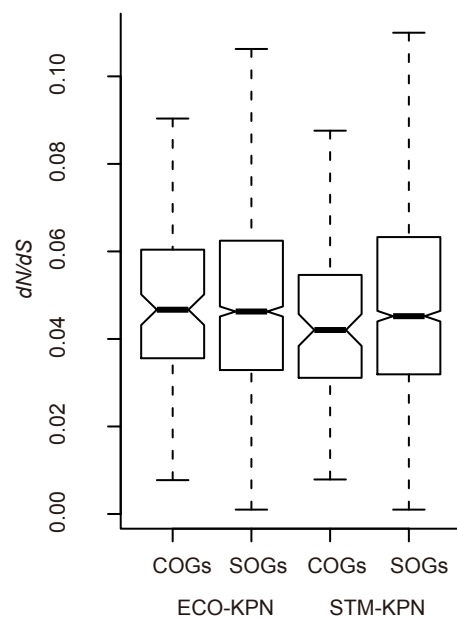

Supplement: Additional file 3 — The evolutionary rates of COGs and SOGs in the ECO-KPN and STM-KPN comparisons. The genes included in this analysis were identified by both reciprocal BLASTP and OMA database as orthologous genes. *: p-value < 0.05; **: p-value < 0.01; ***: p-value < 0.0001 [file 1471-2105-12-S9-S19-S3.pdf]

(a)

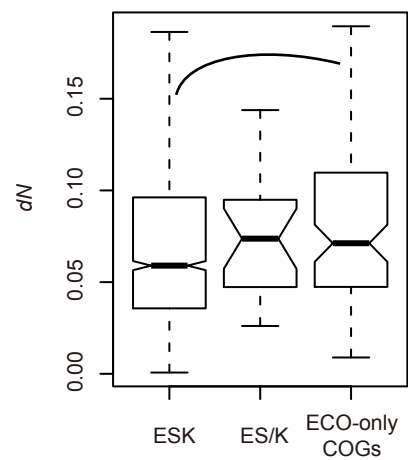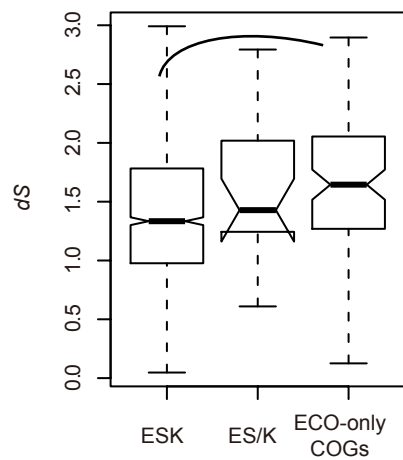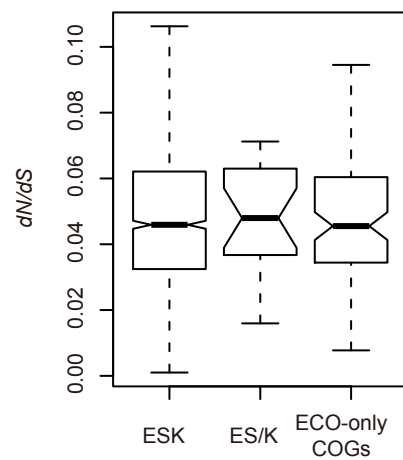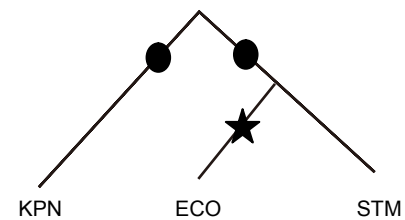

Star: ECO-only COGs  
Solid circle: ES/K

(b)

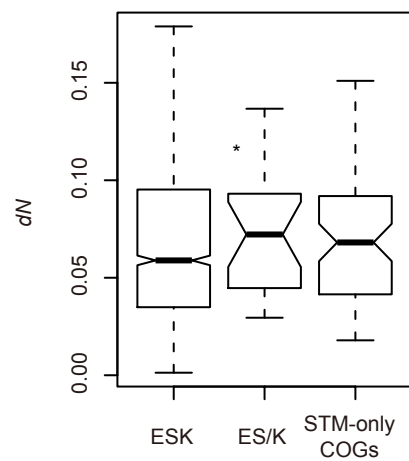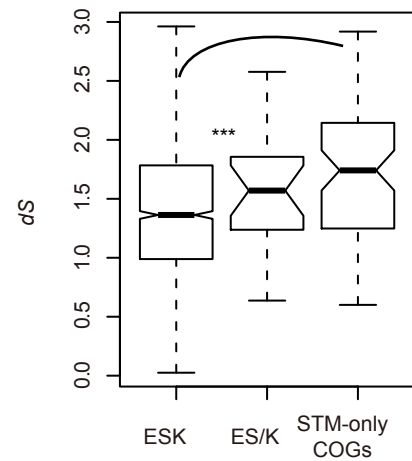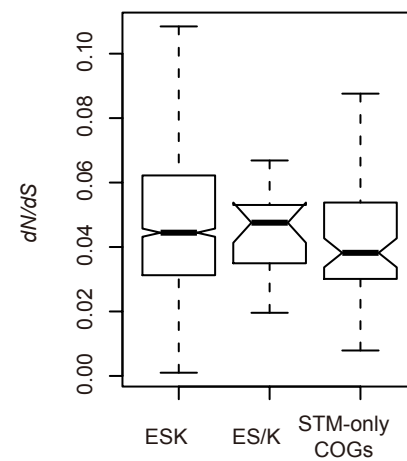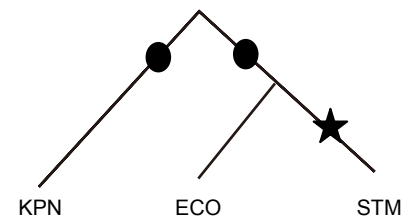

Star: STM-only COGs  
Solid circle: ES/K

Supplement: Additional file 5 — The evolutionary rates in (a) ECO-KPN comparison; and (b) STM-KPN comparison. Here we compare the evolutionary rates of the genes that never changed transcriptional orientation in ECO, STM, and KPN, (“ESK”), the genes that changed orientation in either the ECO-STM lineage or in the KPN lineage (“ES/K”), and the genes that changed orientation in only one species (“ECO-only” or “STM-only”). [file 1471-2105-12-S9-S19-S5.pdf]

(a)

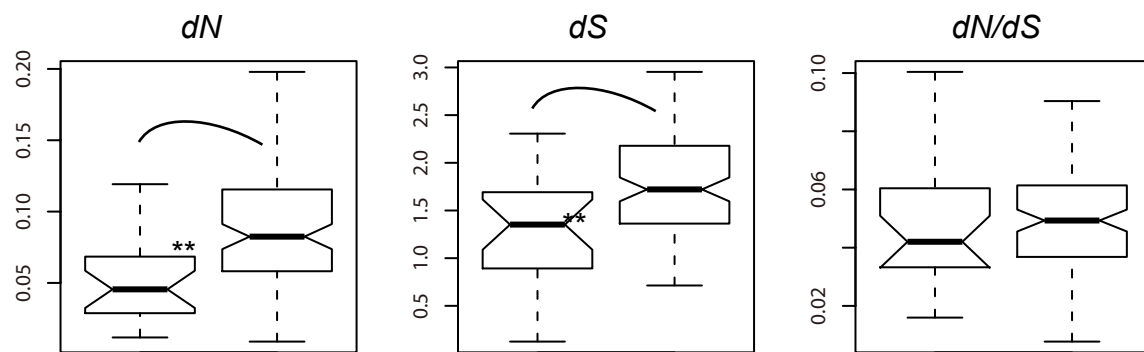

(b)

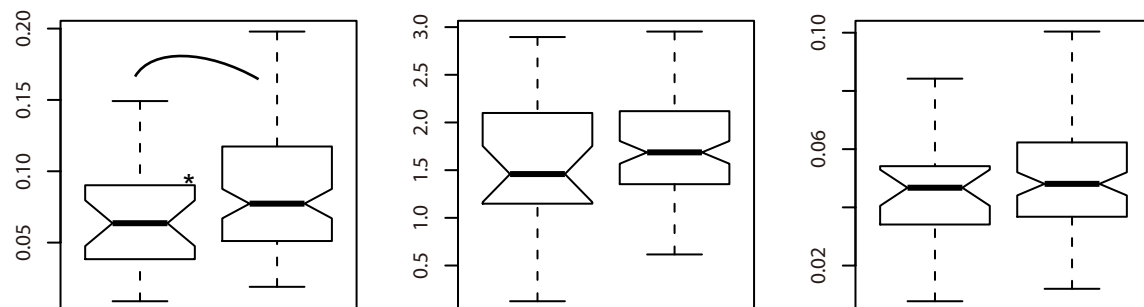

(c)

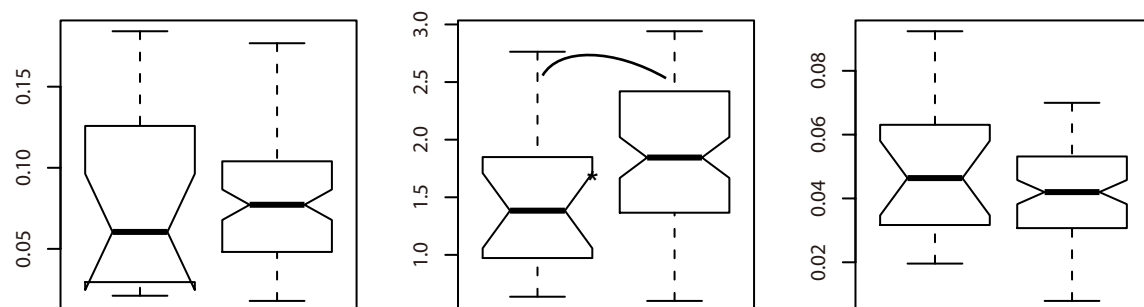

(d)

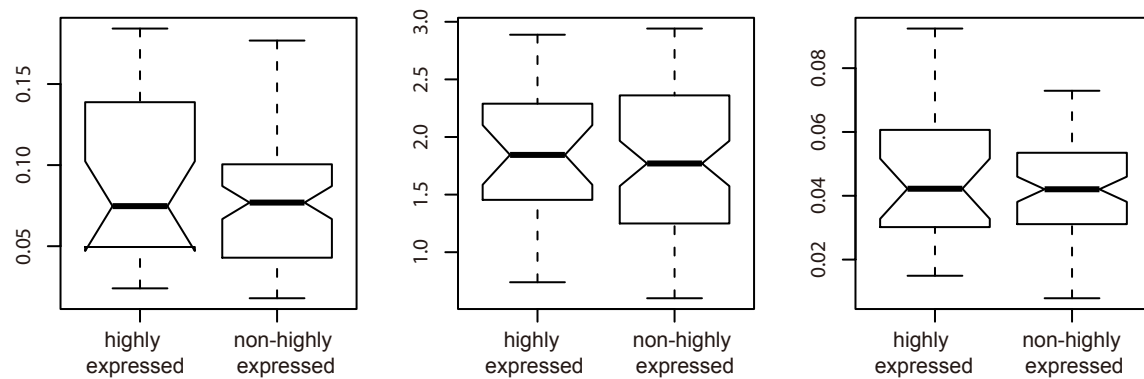

Supplement: Additional file 6 — Comparison of the evolutionary rates of highly and non-highly expressed COGs. (a) ECO-KPN comparison at the log phase; (b) ECO-KPN comparison at the stationary phase; (c) STM-KPN comparison at the log phase; (d) STM-KPN comparison at the stationary phase. *: p-value < 0.05; **: p-value < 0.01 [file 1471-2105-12-S9-S19-S6.pdf]

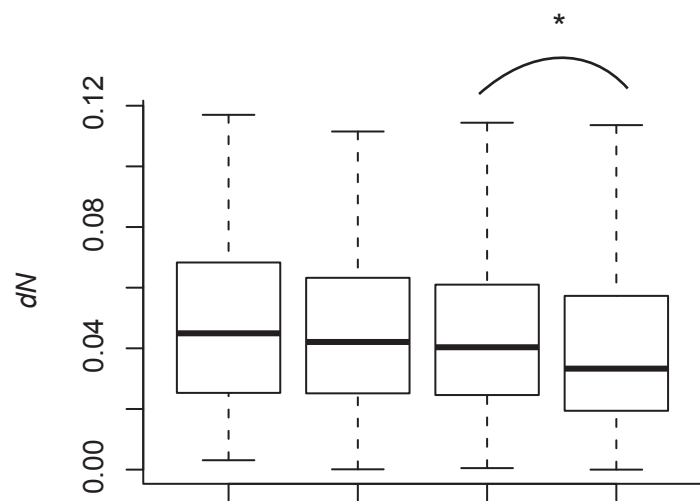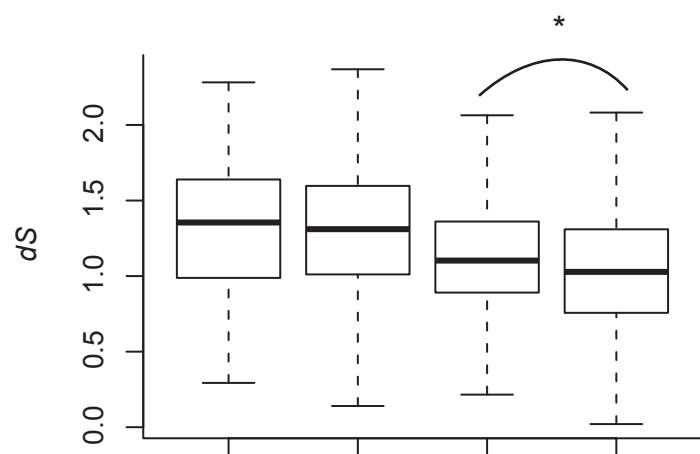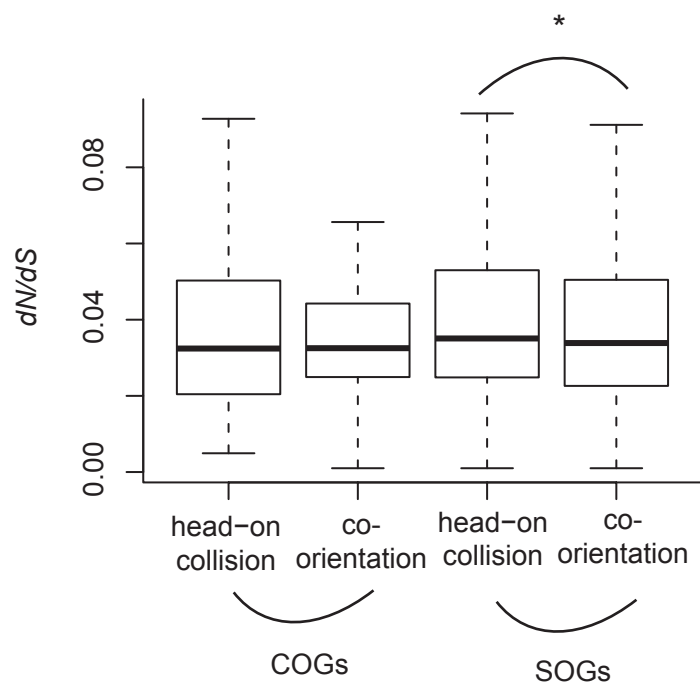

Supplement: Additional file 8 — The evolutionary rates of COGs and SOGs that are subject to head-on collision or co-orientation between DNA replication and transcription in the ECO-STM comparison. * represents p value < 0.05. [file 1471-2105-12-S9-S19-S8.pdf]

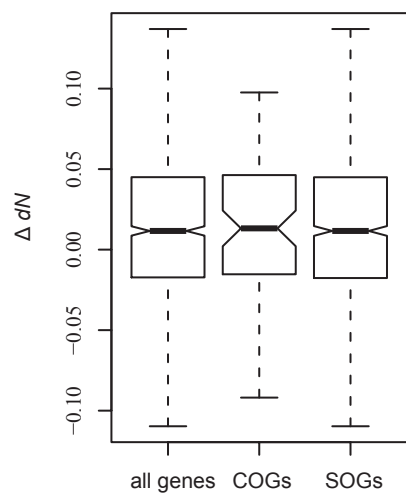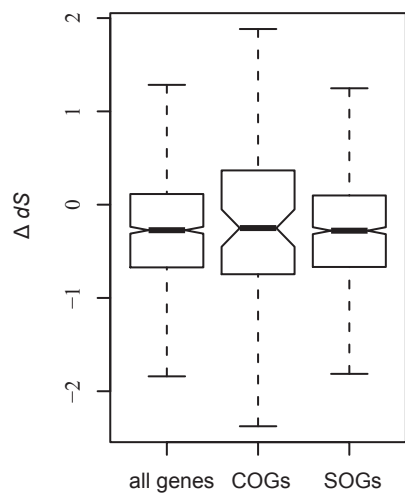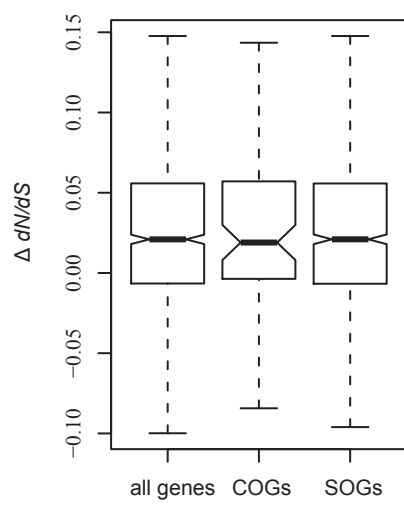

Supplement: Additional file 9 — Comparison of ΔdN, ΔdS and ΔdN/dS between COGs and SOGs in the STM-KPN comparison. ΔdN, ΔdS and ΔdN/dS were calculated by subtracting the evolutionary rates of the middle region from those of the terminal regions of each gene. Note that none of the pair-wise comparisons in any of the three panels is statistically significant. [file 1471-2105-12-S9-S19-S9.pdf]
