# Supplementary material for: Changes in transcriptional orientation are associated with increases in evolutionary rates of enterobacterial genes
Source: BMC Bioinformatics. 2011 Oct 5;12(Suppl 9):S19. doi: 10.1186/1471-2105-12-S9-S19 (PMC3283321; doi:10.1186/1471-2105-12-S9-S19)
Supplement: Additional file 4 — The numbers of COGs with dS > 3 in the ECO-KPN, ECO-STM, and STM-KPN comparisons. [file 1471-2105-12-S9-S19-S4.pdf]

comparison	# genes with *dS* > 3	# COGs with *dS* >3	% COGs with *dS* >3	% COGs in all of the analyed genes
ECO-KPN	385	84	21.82%	7.23%
ECO-STM	103	36	34.95%	12.42%
STM-KPN	358	53	14.80%	5.86%
