# Supplementary material for: Changes in transcriptional orientation are associated with increases in evolutionary rates of enterobacterial genes
Source: BMC Bioinformatics. 2011 Oct 5;12(Suppl 9):S19. doi: 10.1186/1471-2105-12-S9-S19 (PMC3283321; doi:10.1186/1471-2105-12-S9-S19)
Supplement: Additional file 7 — Partial correlations between evolutionary rates and the COG/SOG gene type while the physical distance from Ter is controlled. [file 1471-2105-12-S9-S19-S7.pdf]

	dN		*dS*		*dN/dS*	
	ρ	*p*-value	ρ	*p*-value	ρ	*p*-value
ECO-KPN comparison						
gene type | distance from *Ter*	0.0371	0.117	0.0520	0.028	0.0178	0.452
STM-KPN comparison						
gene type | distance from *Ter*	0.0087	0.713	0.0647	0.006	-0.0408	0.083
