# Supplementary material for: Changes in transcriptional orientation are associated with increases in evolutionary rates of enterobacterial genes
Source: BMC Bioinformatics. 2011 Oct 5;12(Suppl 9):S19. doi: 10.1186/1471-2105-12-S9-S19 (PMC3283321; doi:10.1186/1471-2105-12-S9-S19)
Supplement: Additional file 10 — Functional enrichment analyses for COGs in the (a) ECO-KPN and (b) ECO-STM comparisons. Note that the functional assignments were based on the gene annotations for ECO. [file 1471-2105-12-S9-S19-S10.pdf]

(a)

Annotation Cluster[a]	Gene Ontology (GO) term	Fold Enrichment	p-vaue[b]
1(14.48)	organelle inner membrane	7.07	2.77E-16
	organelle envelope	7.07	2.77E-16
	organelle membrane	6.90	5.44E-16
	organelle part	6.46	4.88E-15
	intracellular organelle part	6.46	4.88E-15
	membrane-bounded organelle	6.25	9.77E-15
	organelle	4.31	6.17E-11
	intracellular organelle	4.31	6.17E-11
	envelope	2.63	2.19E-06
2(13.23)	peptidoglycan-based cell wall	5.32	7.81E-19
	cell wall	5.24	1.34E-18
	external encapsulating structure	2.85	2.39E-10
	envelope	2.63	4.98E-08
3(6.85)	anaerobic respiration	14.07	8.58E-10
	energy derivation by oxidation of organic compounds	6.24	3.46E-07
	cellular respiration	6.53	6.68E-07
	generation of precursor metabolites and	4.25	2.04E-06
4(5.95)	nitrate assimilation	54.12	3.50E-12
	nitrate metabolic process	35.70	1.30E-10
	nitrate reductase activity	25.53	3.00E-07
	nitrate reductase complex	21.50	7.22E-07
	oxidoreductase activity, acting on other nitrogenous compounds as donors	17.02	3.38E-06
5(5.21)	metal ion binding	2.31	3.89E-06
	cation binding	2.24	7.33E-06
	ion binding	2.23	8.19E-06

(b)

Annotation Cluster[a]	Gene Ontology (GO) term	Fold Enrichment	p-vaue[b]
1(17.56)	peptidoglycan-based cell wall	4.66	3.94E-27
	cell wall	4.59	9.59E-27
	external encapsulating structure	2.43	2.12E-12
	envelope	2.02	7.35E-07
2(17.46)	organelle membrane	5.50	6.80E-22
	organelle envelope	5.51	1.89E-21
	organelle inner membrane	5.51	1.89E-21
	organelle part	5.15	9.79E-21
	intracellular organelle part	5.15	9.79E-21
	membrane-bounded organelle	4.98	3.78E-20
	organelle	3.54	2.56E-15

	intracellular organelle	3.54	2.56E-15
	envelope	2.02	7.35E-07
3(8.52)	anaerobic respiration	10.06	1.37E-11
	energy derivation by oxidation of organic compounds	4.95	4.08E-09
	cellular respiration	4.56	4.81E-07
4(7.89)	amine metabolic process	3.04	8.44E-11
	cellular amino acid metabolic process	3.09	4.37E-08
	cellular amine metabolic process	2.92	5.66E-08
	cellular amino acid and derivative metabolic process	2.81	1.30E-07
5(7.74)	carboxylic acid metabolic process	2.74	8.75E-09
	oxoacid metabolic process	2.74	8.75E-09
	organic acid metabolic process	2.66	1.90E-08
	cellular ketone metabolic process	2.61	3.16E-08
	cellular amino acid metabolic process	3.09	4.37E-08
6(6.23)	metal ion binding	2.00	3.96E-07
	ion binding	1.97	5.13E-07
	cation binding	1.95	1.01E-06
7(5.54)	nitrate reductase activity	15.38	8.05E-07
	nitrate reductase complex	12.92	2.26E-06
	oxidoreductase activity, acting on other nitrogenous compounds as donors	10.26	1.28E-05
8(4.31)	tryptophan biosynthetic process	23.52	4.57E-06
	indolalkylamine biosynthetic process	23.52	4.57E-06
	indole derivative biosynthetic process	23.52	4.57E-06
	indole and derivative metabolic process	21.71	6.95E-06
	indole derivative metabolic process	21.71	6.95E-06
	tryptophan metabolic process	21.71	6.95E-06
	indolalkylamine metabolic process	21.71	6.95E-06
	aromatic amino acid family biosynthetic	9.90	1.59E-05
	biogenic amine biosynthetic process	9.90	1.59E-05
	chorismate metabolic process	9.77	1.73E-05
	aromatic amino acid family metabolic	9.29	2.42E-05
	biogenic amine metabolic process	5.72	1.83E-04
	cellular amino acid derivative biosynthetic process	6.60	2.13E-04
	cellular amino acid derivative metabolic	3.90	2.24E-03
	aromatic compound biosynthetic process	3.67	6.28E-03
	cellular aromatic compound metabolic	1.92	7.81E-02
9(4.05)	plasma membrane	1.94	2.61E-11
	membrane part	1.18	3.40E-02
	membrane	0.95	8.08E-01
10(3.93)	peptidoglycan metabolic process	4.77	1.10E-04
	glycosaminoglycan metabolic process	4.77	1.10E-04
	aminoglycan metabolic process	4.66	1.33E-04
11(3.59)	peptide transport	11.76	1.07E-09
	establishment of protein localization	2.47	4.59E-03
	protein transport	2.47	4.59E-03
	protein localization	2.43	5.27E-03
	macromolecule localization	2.25	9.57E-03

12(3.46)	tRNA aminoacylation for protein translation	9.53	2.05E-05
	tRNA aminoacylation	9.53	2.05E-05
	amino acid activation	9.53	2.05E-05
	translation	3.72	1.55E-03
	aminoacyl-tRNA ligase activity	6.82	1.87E-03
	ligase activity, forming aminoacyl-tRNA and related compounds	6.74	1.97E-03
	ligase activity, forming carbon-oxygen	6.74	1.97E-03
	tRNA metabolic process	3.52	2.23E-03
13(3.25)	nucleotide binding	1.67	1.58E-04
	purine ribonucleotide binding	1.80	1.71E-04
	ribonucleotide binding	1.80	1.71E-04
	ATP binding	1.80	3.35E-04
	adenyl ribonucleotide binding	1.79	3.53E-04
	purine nucleotide binding	1.64	9.93E-04
	adenyl nucleotide binding	1.62	1.95E-03
	purine nucleoside binding	1.62	1.95E-03
	nucleoside binding	1.60	2.63E-03

[a]The annotation clusterings are shown with enrichment score > 3.00.

[b]The GO terms are list with p-value < 0.05.
